# Tea intake among adults in 185 countries between 1990 and 2018: population based study

**DOI:** 10.1038/s41538-026-00817-4

**Published:** 2026-04-10

**Authors:** Xunliang Li, Jingjing Zhang, Zhuqing Jin, Guangyan Nie, Lu Xu, Yamei Cheng, Haifeng Pan, Deguang Wang

**Affiliations:** 1 Department of Nephrology, the Second Affiliated Hospital of Anhui Medical University, Hefei, China; 2 Department of Epidemiology and Biostatistics, School of Public Health, Anhui Medical University, Hefei, China

**Keywords:** Diseases, Health care, Medical research

## Abstract

Tea is the world’s second most consumed beverage, yet comprehensive global data on intake patterns remain limited. Using individual-level dietary data from the Global Dietary Database 2018, encompassing 1224 surveys across 185 countries, we assessed tea intake among adults aged ≥20 years from 1990 to 2018, stratified by age, sex, education, and urbanicity. In 2018, estimated global mean intake was 6.18 cups/week (95% uncertainty interval: 5.66–6.82), with threefold regional variation ranging from 3.85 cups/week in Latin America and the Caribbean to 8.95 in the Middle East and North Africa. The highest national intakes were in Iran (17.46 cups/week), Japan (14.95), and Afghanistan (13.74). Intake increased modestly with age but showed minimal differences by sex, education, or urbanicity. Globally, estimated intake rose from 1990 to 2018 (estimated annual percentage change [EAPC]: 0.94%), with the largest regional increase in Southeast and East Asia (EAPC: 1.68%), driven primarily by China (EAPC: 3.04%), while traditionally high-consuming countries such as Georgia showed declining trends (EAPC: −5.91%). These findings highlight substantial global heterogeneity in tea consumption and provide a foundation for nutritional surveillance worldwide.

## Introduction

Tea, the world’s second most consumed beverage after water, represents a significant component of global dietary patterns with important public health implications^1,2^. Extensive research has demonstrated tea’s potential health benefits, including antioxidant properties, cardiovascular protection, and reduced risk of certain cancers and neurodegenerative diseases^3–5^. Green and black teas contain bioactive compounds such as catechins, theaflavins, and flavonoids that contribute to these protective effects^6,7^. However, tea intake patterns vary dramatically across populations, influenced by cultural traditions, economic factors, and geographical availability^8^. Understanding global tea intake patterns is essential for assessing population exposure to these bioactive compounds and their potential health impacts.

Despite tea’s global significance, comprehensive data on tea intake across countries and population subgroups remain limited. Most studies have focused on specific regions or countries, and systematic global assessments with standardized methodologies are scarce^9,10^. Previous studies have not evaluated tea intake subnationally across important sociodemographic factors such as education, urbanicity, age, and sex in diverse global populations. This evidence gap limits our ability to understand global nutrition transitions, assess population-level exposure to tea’s bioactive compounds, and identify populations where tea intake patterns may influence health outcomes.

Available evidence suggests substantial variation in tea intake globally, with traditionally high intake in Asian countries, parts of the Middle East and North Africa, and certain European regions^11^. However, intake patterns may be changing due to globalization, urbanization, and evolving dietary preferences^12^. Some high-income countries have experienced shifts toward other beverages, while tea intake may be increasing in regions undergoing nutrition transition^13,14^. Harmonized, subnationally stratified data on tea intake are needed to understand these evolving patterns and their implications for population health.

To address these critical knowledge gaps, we analyzed tea intake patterns using data from the Global Dietary Database (GDD) 2018, which provides comprehensive individual-level dietary data across 185 countries^15^. We examined tea intakes among adults aged ≥20 years at global, regional, and national levels and trends over time from 1990 to 2018, jointly stratified at subnational level by age, sex, education level, and area of residence.

## Results

### Global, regional, and national tea intakes in 2018

**Fig. 1:**
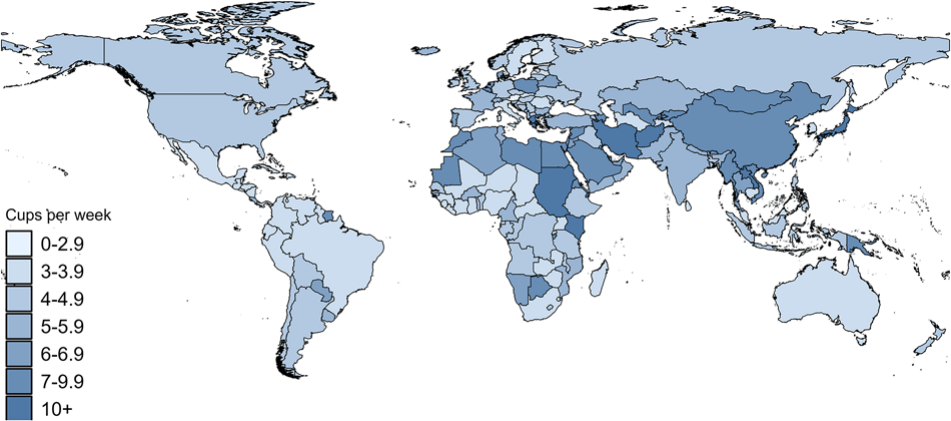
National mean intakes of tea (cup (8oz) per week) in adults aged ≥ 20 years across 185 countries in 2018. The standardized serving size used for this analysis was a cup (8oz). Total green or black tea intake, including caffeinated, decaffeinated, sweetened, or unsweetened tea. This definition excludes herbal tea.

In 2018, the estimated mean global intake of tea among adults aged ≥20 years was 6.18 cups (8oz) per week (95% uncertainty interval [UI] 5.66–6.82), with substantial (threefold) variation across world regions, from 3.85 cups/week (3.52–4.25) in Latin America and the Caribbean to 8.95 cups/week (7.94–10.29) in the Middle East and north Africa (Table 1). Among the 25 countries with the largest adult population worldwide, the highest estimated mean intakes were in Iran (17.46 cups/week (16.55–18.50)), followed by Japan (14.95 (12.74–17.54)), Afghanistan (13.74 (9.40–20.41)), Kenya (13.09 (11.66–14.72)), and Sudan (13.18 (10.06–17.68)); while the lowest estimated intakes were in Mexico (3.53 cups/week (3.18–3.92)) and South Africa (3.38 (3.12–3.67)) (Fig. 1, Supplementary Tables 1 and 2). Of the 185 countries included in the analysis, 41 (22.2%) had estimated mean tea intakes of ≥7 cups/week, representing approximately 1.6 billion adults aged ≥20 years, or 32.7% of the global population for this age group.

**Table 1:** Global and regional mean tea intake (cup (8oz) serving/week) in adults aged ≥ 20 years, by sex, age, education, and area of residence across 185 countries in 2018

Mean (95% UI)								
	**Worldwide**	**Central and eastern Europe and central Asia**^**a**^	**High income countries**	**Latin America and the Caribbean**	**Middle East and North Africa**	**South Asia**^**a**^	**Southeast and East Asia**	**Sub-Saharan Africa**
**Overall**	6.18 (5.66–6.82)	5.45 (4.66–6.45)	4.88 (4.53–5.31)	3.85 (3.52–4.25)	8.95 (7.94–10.29)	5.46 (4.93–6.07)	7.65 (6.32–9.38)	4.94 (4.58–5.37)
Sex								
Female	6.24 (5.64–7.03)	5.47 (4.59–6.68)	5.01 (4.61–5.52)	3.90 (3.54–4.33)	8.73 (7.68–10.10)	5.51 (4.96–6.16)	7.81 (6.24–10.05)	4.99 (4.60–5.46)
Male	6.10 (5.52–6.84)	5.42 (4.60–6.50)	4.74 (4.37–5.20)	3.80 (3.45–4.22)	9.16 (8.11–10.59)	5.41 (4.85–6.06)	7.43 (5.93–9.44)	4.89 (4.50–5.35)
Age (years)								
20–39	5.87 (5.42–6.43)	5.68 (4.91–6.64)	4.47 (4.17–4.85)	3.86 (3.56–4.23)	8.35 (7.43–9.59)	5.14 (4.75–5.57)	7.37 (6.11–9.03)	4.86 (4.54–5.24)
40–59	6.41 (5.83–7.14)	5.47 (4.66–6.50)	5.02 (4.66–5.47)	3.94 (3.61–4.34)	9.51 (8.42–10.95)	5.78 (5.21–6.41)	7.67 (6.29–9.47)	5.10 (4.72–5.54)
≥60	6.36 (5.72–7.19)	5.13 (4.35–6.19)	5.00 (4.59–5.49)	3.60 (3.18–4.10)	10.46 (9.23–12.05)	5.87 (4.87–7.06)	7.79 (6.29–9.82)	4.94 (4.35–5.63)
Education (years)								
0–6	6.05 (5.44–6.87)	5.88 (4.88–7.25)	4.58 (3.99–5.37)	3.82 (3.47–4.24)	8.82 (7.76–10.29)	5.44 (4.87–6.11)	7.22 (5.55–9.60)	4.87 (4.49–5.33)
>6–12	6.24 (5.54–7.20)	5.52 (4.76–6.53)	4.90 (4.38–5.57)	3.86 (3.53–4.24)	8.46 (7.39–9.89)	5.50 (4.97–6.10)	7.73 (6.00–10.20)	5.19 (4.80–5.65)
>12	6.25 (5.65–7.06)	5.28 (4.36–6.56)	4.93 (4.65–5.26)	3.91 (3.47–4.47)	9.90 (8.88–11.23)	5.40 (4.75–6.22)	8.01 (6.42–10.30)	4.75 (4.36–5.18)
Area of residence								
Rural	6.13 (5.52–6.91)	5.65 (4.92–6.60)	4.73 (4.37–5.15)	3.89 (3.47–4.40)	9.25 (8.20–10.61)	5.47 (4.92–6.10)	7.26 (5.72–9.40)	5.04 (4.65–5.51)
Urban	6.20 (5.63–6.96)	5.33 (4.43–6.52)	4.92 (4.55–5.38)	3.84 (3.52–4.23)	8.80 (7.76–10.22)	5.45 (4.89–6.10)	7.89 (6.38–9.98)	4.79 (4.41–5.24)

*UI* uncertainty interval.

The standardized serving size used for this analysis was a cup (8oz). Total green or black tea intake, including caffeinated, decaffeinated, sweetened, or unsweetened tea. This definition excludes herbal tea.

^a^In previous Global Dietary Database reports, the region central or eastern Europe and central Asia was referred to as the former Soviet Union, and southeast and east Asia was referred to as Asia.

### Tea intake by sex and age in 2018

**Fig. 2:**
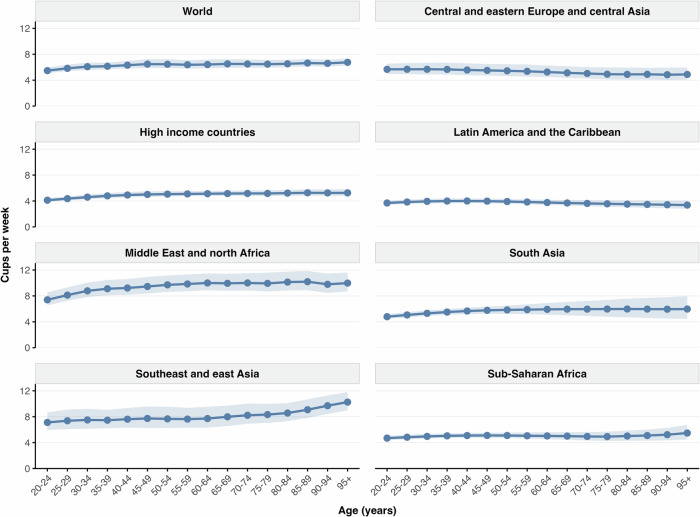
Global and regional intakes of tea (cup (8oz) per week) by age in adults aged ≥ 20 years in 2018. The standardized serving size used for this analysis was a cup (8oz). Total green or black tea intake, including caffeinated, decaffeinated, sweetened, or unsweetened tea. This definition excludes herbal tea. The filled circles represent the mean tea intake (cup (8oz) per week) and the shaded areas the 95% UIs. In previous Global Dietary Database reports, the region central and eastern Europe and central Asia was referred to as the former Soviet Union, and southeast and east Asia was referred to as Asia. UI uncertainty interval.

Globally, estimated tea intakes between male and female adults did not differ substantially (females: 6.24 cups/week, 5.64 to 7.03; males: 6.10, 5.52 to 6.84), as observed by the overlapping 95% UIs (Table 1, Supplementary Tables 3 and 4, and Supplementary Fig. 2). This pattern of minimal sex differences was consistent across regions and individual countries. Estimated intake of tea in adults generally increased with age globally and regionally, although with varying magnitude of these differences by region (Table 1 and Supplementary Fig. 2). For instance, estimated intakes of tea exceeded 10 cups/week among adults aged ≥60 years in the Middle East and north Africa but were approximately 5 cups/week among adults of the same age in South Asia. Regionally, patterns of estimated intake by age were similar between sexes (Supplementary Fig. 2). Considering both age and region, the highest estimated weekly intakes of tea were in the Middle East and north Africa in adults aged ≥60 years (10.46 cups/week) and lowest in Latin America and the Caribbean in adults aged 20-39 years (3.86 cups/week) (Table 1 and Fig. 2). Among the 25 most populous countries, the highest estimated intakes of tea were in Iran among adults aged ≥60 years (19.33 cups/week) and lowest in Mexico among adults aged ≥60 years (3.33 cups/week) (Supplementary Table 2).

### Tea intake by education and urbanicity in 2018

**Fig. 3:**
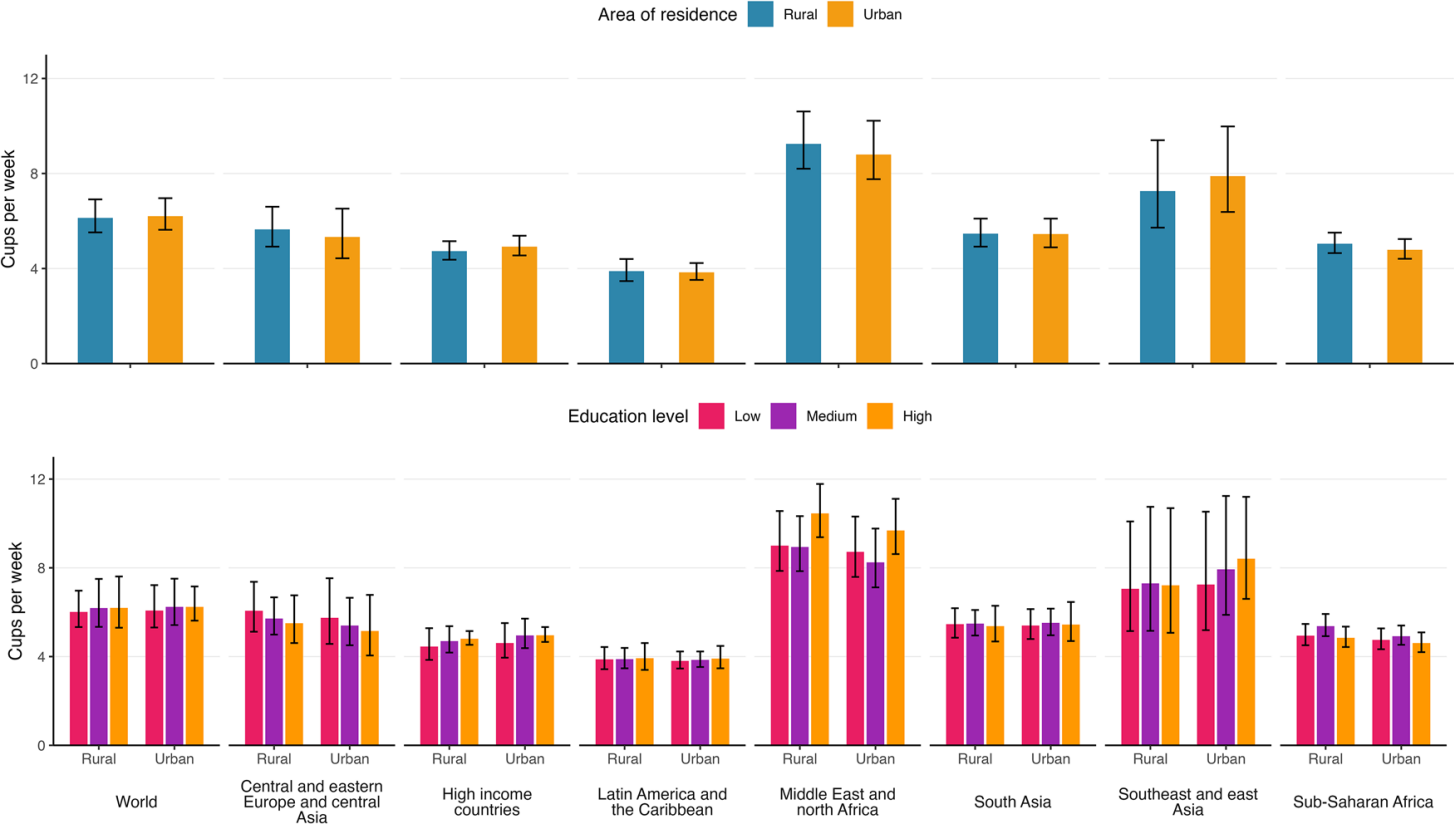
Global and regional mean tea intakes (cup (8oz) per week) in adults aged ≥ 20 years by area of residence and education level in 2018. The standardized serving size used for this analysis was a cup (8oz). Total green or black tea intake, including caffeinated, decaffeinated, sweetened, or unsweetened tea. This definition excludes herbal tea. Error bars represent 95% UIs. In previous Global Dietary Database reports, the region central and eastern Europe and central Asia was referred to as the former Soviet Union, and southeast and east Asia was referred to as Asia. UI uncertainty interval.

Estimated tea intakes showed minimal association with educational attainment globally, with values that were nearly identical across education levels. Adults with higher education (>12 years) had estimated consumption of 6.25 cups/week (5.65–7.06), compared to 6.24 (5.54–7.20) for those with 6–12 years of education and 6.05 (5.44–6.87) for those with ≤6 years of education (Table 1). Estimated tea intakes were slightly higher in urban areas compared to rural areas globally (6.20 cups/week, 5.63–6.96 vs 6.13, 5.52–6.91), though differences were modest (Table 1). When education and urbanicity were assessed jointly, the pattern of minimal educational gradient was consistently observed (Fig. 3). Regional patterns varied considerably, with the Middle East and North Africa showing the highest estimated intakes across all education-urbanicity strata (≥8 cups/week each), while other regions demonstrated more heterogeneous patterns. Within regions, the relationship between education level and estimated tea intake was inconsistent, with some regions showing higher estimated intake among more educated populations, others showing minimal educational gradients, and some showing inverse patterns. See Supplementary Tables 5–8 for further details on estimated tea intakes by education and area of residence.

### Trends in tea intake during 1990–2005, 2005–2018, and 1990–2018

**Fig. 4:**
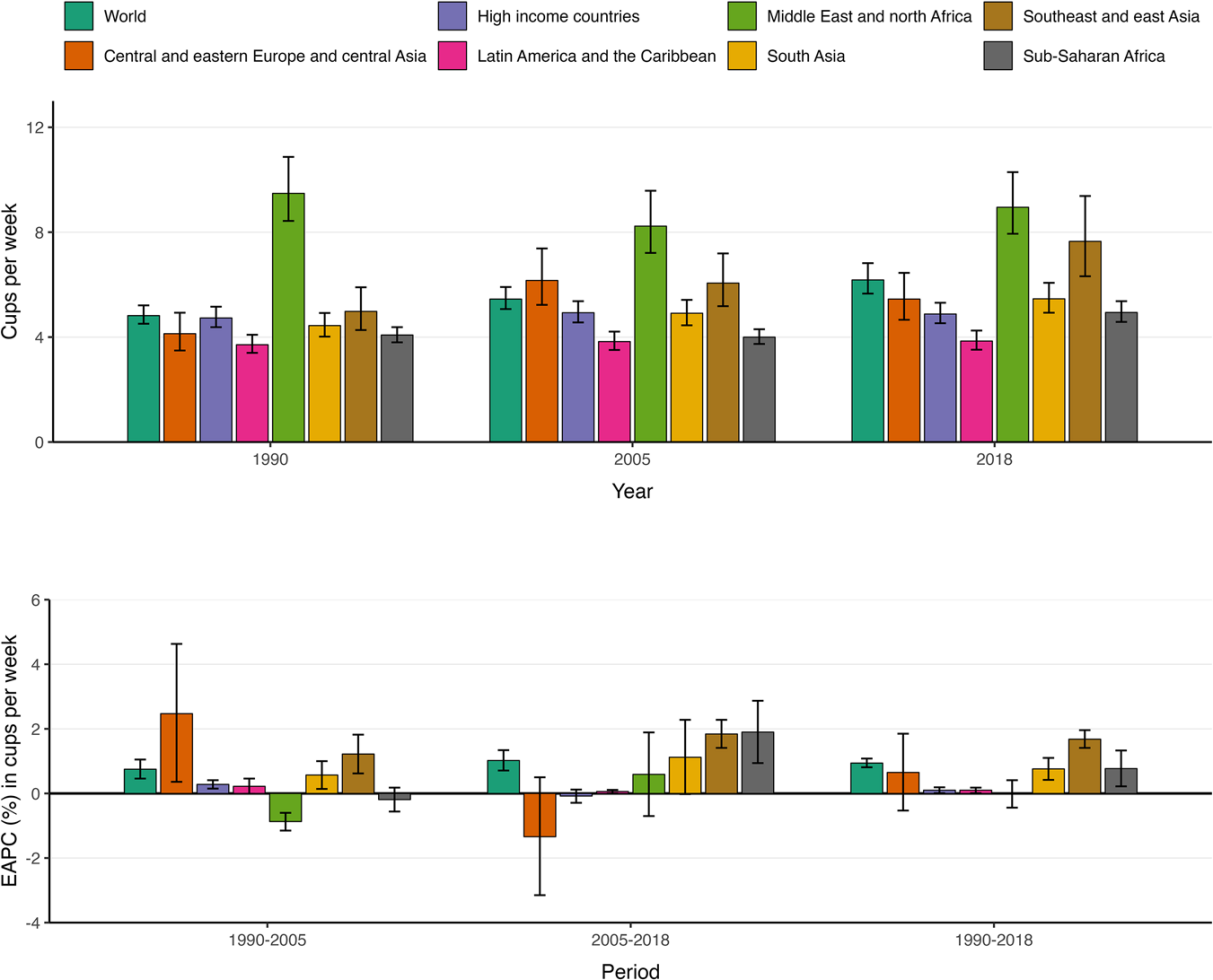
Mean tea intakes (cup (8oz) per week) by world region in 1990, 2005, and 2018, and EAPCs from 1990 to 2005, 2005 to 2018, and 1990 to 2018 in adults aged ≥ 20 years. (Top panel) Mean tea intakes by world region in 1990, 2005, and 2018. (Bottom panel) EAPCs in tea intakes from 1990 to 2005, 2005 to 2018, and 1990 to 2018. The standardized serving size used for this analysis was a cup (8oz). Total green or black tea intake, including caffeinated, decaffeinated, sweetened, or unsweetened tea. This definition excludes herbal tea. Error bars represent 95% UIs or 95% CIs. In previous Global Dietary Database reports, the region central and eastern Europe and central Asia was referred to as the former Soviet Union, and southeast and east Asia was referred to as Asia. CI confidence interval, EAPC estimated annual percentage change, UI uncertainty interval.

Supplementary Tables 9–12 and Supplementary Figs. 3–6 show absolute global, regional, and national estimated intakes of tea for 1990 and 2005. Globally, from 1990 to 2018, estimated intakes among adults increased with an estimated annual percentage change (EAPC) of 0.94% (95% confidence interval [CI] 0.81–1.08) (Fig. 4 and Supplementary Table 1). The global increase showed variation across time periods: 1990–2005 (EAPC 0.75% (0.46–1.05)) and 2005 to 2018 (EAPC 1.02% (0.71–1.34)). However, regionally, changes did not follow the same global pattern. Between 1990 and 2005, increases in estimated intakes of tea were observed in most regions, with the largest increase in central and eastern Europe and central Asia (EAPC 2.47% (0.36–4.63)), little change in south Asia (EAPC 0.57% (0.14–1.00)), and a decrease in the Middle East and north Africa (EAPC −0.87% (−1.15 to −0.60)). More recently, from 2005 to 2018, increases continued in most regions, with the largest in southeast and east Asia (EAPC 1.84% (1.41–2.28)), except for central and eastern Europe and central Asia where estimated intakes decreased (EAPC −1.34% (−3.15 to 0.50)) and high income countries where little change was evident (EAPC −0.08% (−0.29 to 0.12)). In the overall period from 1990 to 2018, the largest regional increase in estimated intake was in Southeast and East Asia (EAPC 1.68% (1.41–1.96)), with other world regions showing more modest increases over time. Exceptions were central and eastern Europe, central Asia, the Middle East, and north Africa, where estimated intakes showed minimal change overall (Supplementary Table 13).

Among the 25 most populous countries, the largest estimated increase from 1990 to 2005 was in Uzbekistan (EAPC 4.67% (3.25–6.10)) and the largest estimated decrease was in Georgia (EAPC −3.47% (−5.86 to −1.01)) (see Supplementary Table 1). From 2005 to 2018, the largest estimated increase was in Afghanistan (EAPC 8.27% (4.64–12.02)), and the largest estimated decrease was in Georgia (EAPC −6.95% (−14.96 to 1.81)). Overall, between 1990 and 2018, the largest estimated increase was in China (EAPC 3.04% (2.33 to 3.76)) and the largest estimated decrease was in Georgia (EAPC −5.91% (−8.03 to −3.75)) (Supplementary Table 1).

### Tea intakes and trends by sociodemographic development index

**Fig. 5:**
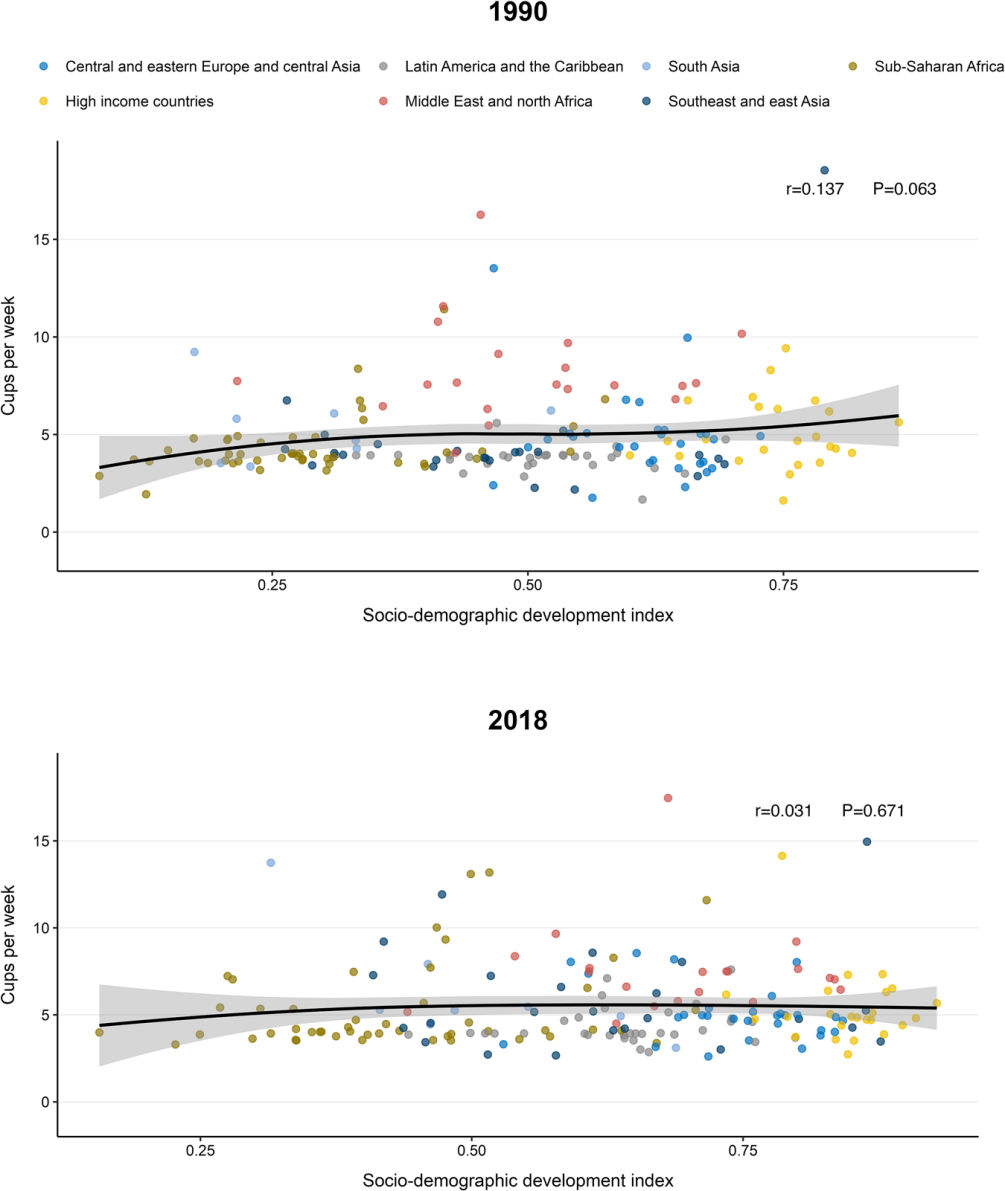
National correlation of tea intake (cup (8oz) per week) in adults aged ≥ 20 years and socio-demographic development index by world region in 1990 and 2018 for 185 countries. Spearman correlation was assessed between SDI and tea intakes among a total of 185 countries that were included in this analysis. The shaded areas the 95% CIs. Total green or black tea intake, including caffeinated, decaffeinated, sweetened, or unsweetened tea. This definition excludes herbal tea. SDI was obtained from the Global Burden of Disease study 2021. CI confidence interval.

In 1990 and 2018, no clear correlation was evident between national estimated intakes of tea and sociodemographic development index (SDI) (*r* = 0.137, *P* = 0.063; *r* = 0.031, *P* = 0.671; respectively) (Fig. 5). However, in 2005, a correlation was present between estimated tea intake and SDI (*r* = 0.300, *P* < 0.001) (Supplementary Fig. 7).

### Equally-weighted regional estimates

Equally weighted regional estimates showed generally consistent patterns with population-weighted estimates but revealed some important differences (Supplementary Tables 1 and 14). In Southeast and East Asia, the population-weighted estimate (7.65 cups/week in 2018) substantially exceeded the equally weighted estimate (5.91 cups/week). Similarly, the population-weighted EAPC from 1990 to 2018 (1.68%) was higher than the equally weighted EAPC (0.88%). Conversely, in South Asia, the equally weighted estimate (6.27 cups/week) exceeded the population-weighted estimate (5.46 cups/week).

## Discussion

Global estimated tea intake among adults aged ≥20 years increased modestly from 1990 to 2018, with an EAPC of 0.94%, reaching an estimated mean intake of 6.18 cups per week in 2018. We observed substantial heterogeneity across regions, countries, and time periods, with a threefold variation between the lowest consuming region (Latin America and the Caribbean) and highest (Middle East and North Africa). These findings provide the first comprehensive global assessment of tea intake patterns using individual-level dietary data within a unified analytical framework, revealing important insights for nutritional surveillance and dietary pattern characterization worldwide.

The regional variations in estimated tea intake reflect complex interactions between cultural traditions, economic development, and dietary transitions^12^. The Middle East and North Africa maintained the highest estimated regional intakes throughout the study period, consistent with the deep cultural significance of tea in these societies, where tea intake is integral to social customs and hospitality practices^16^. Iran’s estimated high intake (17.46 cups/week) in GDD model aligns with the country’s strong tea culture and domestic production capacity^17^. Similarly, estimated high intakes in Kenya (13.09 cups/week) and Afghanistan (13.74 cups/week) in our analysis are consistent with both cultural preferences and local tea production^18^. In contrast, Latin America and the Caribbean showed persistently lower estimated tea intake relative to other regions (3.85 cups/week), likely due to the regional preference for coffee and other traditional beverages like mate and chocolate-based drinks^19,20^.

The most striking temporal change in GDD modeled estimates occurred in southeast and east Asia, where estimated tea intake nearly doubled from 1990 to 2018 (EAPC 1.68%), driven primarily by China’s dramatic estimated increase (EAPC 3.04%). This resurgence in China, the historical birthplace of tea culture, may reflect economic prosperity enabling greater purchasing power, renewed interest in traditional practices, and growing awareness of tea’s health benefits^21,22^. The increase coincides with China’s rapid economic development and may represent a return to traditional intake patterns that were disrupted during periods of economic hardship^23^. Conversely, several traditionally high-consuming countries showed declining trends in our estimates, including Georgia (EAPC −5.91%) and New Zealand (EAPC −2.63%), potentially reflecting westernization of dietary patterns and competition from coffee and other beverages ^24,25^.

Our finding of minimal sex differences in estimated tea intake contrasts with patterns observed for many other dietary components, suggesting that tea intake is less influenced by gender-specific dietary preferences or social norms^26^. The increase in estimated tea intake with age (from 5.87 cups/week in adults aged 20–39 to 6.36 cups/week in those ≥60 years) may reflect both cohort effects and age-related changes in beverage preferences^27,28^. The minimal association with education level in our estimates suggests that tea intake transcends socioeconomic boundaries, unlike many other dietary behaviors that show strong educational gradients^29^.

The slight urban-rural difference in estimated tea intake (6.20 vs 6.13 cups/week) was smaller than might be expected given typical urban-rural dietary disparities^30^. This finding suggests that tea availability and cultural practices around tea intake are relatively consistent across urban and rural settings, unlike processed foods and beverages that typically show stronger urban concentration^31^. However, regional heterogeneity in urban-rural patterns warrants further investigation, particularly in rapidly urbanizing regions where traditional dietary patterns may be under pressure^32^.

The changing relationship between estimated tea intake and SDI—from no correlation in 1990 to a positive correlation in 2005 and back to no correlation in 2018—suggests a complex, potentially non-linear relationship between economic development and tea intake patterns. This may reflect tea’s dual identity as both a traditional beverage in many lower-income settings and a beverage choice in higher-income countries^33,34^. However, these associations describe population-level patterns and should not be interpreted as individual-level relationships between socioeconomic status and tea consumption.

Our analysis has several important strengths. We utilized the most comprehensive GDD available, incorporating 1224 dietary surveys representing approximately 99.0% of the world’s population, with most surveys nationally or subnationally representative^15^. Unlike previous global estimates that relied primarily on production data or sales figures, our estimates derive from individual-level dietary assessments that can capture actual intake patterns and demographic variations^35^. The Bayesian hierarchical modeling approach appropriately accounts for survey heterogeneity and uncertainty, providing robust estimates even for countries with limited primary data^36^. The joint stratification by age, sex, education, and urbanicity provides unprecedented detail on within-country variations in tea intake patterns. The comparison between population-weighted and equally weighted regional estimates revealed that large-population countries, particularly China and India, substantially influence regional averages. However, the consistency of overall patterns between the two approaches—including regional rankings and temporal trends—supports the robustness of our findings. The availability of both estimates enhances transparency and allows readers to assess whether conclusions depend on the weighting approach, which is particularly important for regions with heterogeneous country-level patterns.

Several important limitations should be acknowledged. First, our findings represent modeled estimates derived from hierarchical Bayesian modeling of heterogeneous data sources, rather than direct observations of individual intake. While this approach enables global comparisons under a unified framework and appropriately accounts for survey heterogeneity and data gaps, inter-country differences may partially reflect modeling assumptions, data coverage, and survey methodologies rather than solely true behavioral variation. Therefore, statements regarding relative rankings (e.g., highest or lowest consuming countries or regions) should be interpreted as modeled estimates of mean intake levels under our analytical framework. The study’s primary contribution lies in depicting global and regional trends and patterns using standardized methods, rather than providing definitive rankings of actual consumption behavior across populations. Second, our operational definition of tea excluded herbal teas and other culturally dominant tea-like beverages such as yerba mate, rooibos, and various tisanes, which may constitute important beverages in some cultures^37^. This operational definition likely underestimates actual tea-like beverage consumption in Latin America (where yerba mate is prevalent), parts of Europe, and North Africa, potentially introducing bias in cross-regional comparisons. Furthermore, standardizing serving size to 8oz per cup does not capture substantial cultural variations in brewing strength, serving volume, and additions such as milk or sugar, which limit cross-cultural comparability of the reported intake levels. Future research incorporating broader definitions of tea-like beverages and culture-specific preparation methods would provide more comprehensive assessments. Third, despite the comprehensive data collection efforts, survey availability varied across countries and regions, with some nations having limited individual-level dietary data, particularly in earlier time periods and in lower-income regions. This resulted in higher uncertainty for estimates in data-sparse countries, as reflected in the wide UIs. The standardized harmonization process, while enabling global comparisons, required broad categorizations of age groups, education levels, and urban-rural classifications that may not capture more nuanced demographic differences. Fourth, all dietary assessment methods included in the GDD are subject to measurement errors, including potential recall bias in 24 h dietary recalls and food frequency questionnaires, and systematic differences in reporting accuracy across populations. Finally, this study characterizes tea intake patterns at the population level but does not measure individual-level health outcomes. Therefore, we cannot make causal inferences about the health implications of observed consumption differences across populations or regions. While extensive prior research from individual-level epidemiological studies has demonstrated tea’s potential health benefits—including antioxidant properties, cardiovascular protection, and reduced risk of certain cancers and neurodegenerative diseases^3–5^—these established benefits should not be interpreted as findings from our population-level consumption analysis. Our study provides descriptive data on intake patterns that may inform nutritional surveillance strategies and identify populations with varying exposure to tea consumption, but the relationship between population-level intake patterns and health outcomes requires individual-level longitudinal studies with health endpoints.

Estimated tea intakes among adults aged ≥20 years in 185 countries increased modestly from 1990 to 2018, with substantial heterogeneity showing threefold regional variation and notable increases in Southeast and East Asia. These modeled estimates under a unified analytical framework provide important insights into global consumption patterns and temporal trends in tea intake. Our findings on tea intake patterns may inform nutritional surveillance strategies and dietary assessment programs. However, these patterns represent population-level descriptive data and do not themselves support causal claims about health effects of observed consumption differences. Future research should examine relationships between tea intake and health outcomes at the individual level through appropriately designed epidemiological studies.

## Methods

### Study design

This investigation analyzed tea intake across 185 countries using a serial cross-sectional approach based on data from the GDD 2018^15^. The GDD is a comprehensive repository of dietary survey data with standardized collection protocols, incorporating 1224 dietary surveys from 185 countries, with 89% representative at national or subnational level, covering approximately 99.0% of the global population in 2018. We focused our analysis on adults aged ≥20 years. Supplementary Fig. 1 presents the flow chart illustrating our data extraction and analysis process from the GDD. This investigation was exempt from ethical review board approval because it was based on published de-identified nationally representative data, without personally identifiable information.

### Data sources

The GDD was constructed through systematic online searches for individual level dietary surveys in global and regional databases: PubMed, Embase, Web of science, LILACS, African Index Medicus, and the South-east Asia Index Medicus, using search terms “nutrition” or “diet” or “food habits” or “nutrition surveys” or “diet surveys” or “food habits”[mesh] or “diet”[mesh] or “nutrition surveys”[mesh] or “diet surveys”[mesh] and (“country of interest”). Additionally, surveys were identified through extensive personal communications with researchers and government authorities throughout the world. The search included surveys that collected data on at least one of 54 foods, beverages, nutrients, or dietary indices, including tea. Studies were screened by title and abstract, with a subset screened by a second reviewer to ensure consistency and accuracy, and a third reviewer screened studies to ensure that survey inclusion criteria were met. Surveys were prioritized if they were performed at national or subnational level and used individual-level dietary assessments with standardized 24 h recalls, food frequency questionnaires, or short standardized questionnaires (e.g., Demographic Health Survey questionnaires). When national or subnational surveys at individual level were not identified for a country, individual.level surveys from large cohorts, the World Health Organization (WHO) Global Infobase, and the WHO Stepwise Approach to Surveillance database were included. When individual level dietary surveys were not identified for a particular country, household budget surveys were considered. Surveys focused on special populations (e.g., exclusively pregnant or nursing mothers, individuals with a specific disease) or specific cohorts (e.g., specific occupations or dietary patterns) were excluded. For the present analysis, we utilized tea intake data from this database. Details on the methods and standardized data collection protocol are described in detail elsewhere^15,38,39^.

### Data modeling

The GDD estimates intakes for years for which survey data are available using advanced statistical modeling approaches. To incorporate and deal with differences in data comparability and sampling uncertainty, the database employs a bayesian model with a nested hierarchical structure (with random effects by country and region) to estimate the mean intake and statistical uncertainty for each of 264 population strata across 185 countries for 1990, 1995, 2000, 2005, 2010, 2015, and 2018. The GDD employed standardized definitions and harmonization protocols across all time periods. For surveys conducted in or around 1990, the same inclusion criteria, dietary assessment standardization procedures, and tea definition were applied as for later periods. Quality control procedures ensured comparability of data sources across countries and years. The model incorporates seven world regions: central and eastern Europe and central Asia, high income countries, Latin America and the Caribbean, the Middle East and North Africa, South Asia, Southeast and East Asia, and sub-Saharan Africa. This classification aims to group nations by general similarities in risk profiles and disease outcomes and has been used by other studies (e.g., the Global Burden of Disease study). Although our analysis focuses on adults aged ≥20 years, the database model used all age data to generate the strata predictions, allowing the use of the full set of available data and covariates to inform estimates, including age patterns, relationships between predictors and intakes, and influence of covariates (e.g., dietary assessment methods).

The model’s primary inputs were survey level quantitative data on intakes (by country, time, age, sex, education level, and urban or rural residence), survey characteristics (dietary assessment method, type of dietary metric), and country-year specific covariates. The model included overdispersion of survey level variance for surveys that were not nationally representative or not stratified by smaller age groups (≤10 years), sex, education level, or urbanicity. Survey level covariates addressed potential survey bias, and the overdispersion parameter non-sampling variation due to survey level error (from imperfect study design and quality). The model estimated intakes jointly stratified by age (<1, 1–2, 3–4, 5–9, 10–14, 15–19, 20–24, 25–29, 30–34, 35–39, 40–44, 45–49, 50–54, 55–59, 60–64, 65–69, 70–74, 75–79, 80–84, 85–89, 90–94, ≥95 years), sex, education (≤6 years, >6–12 years, >12 years), and urbanicity (urban, rural). For adults (age ≥20 years) the stratification by education refers to individual education.

The uncertainty of each stratum specific estimate was quantified using 4000 Monte Carlo iterations to determine posterior predictive distributions of mean intake jointly by country, year, and sociodemographic subgroup. The median intake and the 95% UI for each stratum were computed as the 50th, 2.5th, and 97.5th percentiles of the 4000 draws, respectively. For model selection and validation, the database developers compared results from fivefold cross validation (randomly omitting 20% of the survey data at the stratum level and using that to evaluate predictive ability, run five times), compared predicted country intakes with survey observed intakes, assessed implausible estimates, and visually assessed global and national mean intakes using heat maps.

A second Bayesian model was used to strengthen time trend estimates for dietary factors with corresponding available data on food or nutrients from the Food and Agriculture Organization’s food balance sheets or the Global Expanded Nutrient Supply dataset^40^. No time component was formally included in the model; rather, time was captured by the underlying time variation in the model covariates. This second model incorporated country level intercepts and slopes, along with their correlation estimated across countries. The model is commonly referred to as a varying slopes model structure, and it leverages two dimensional partial pooling between intercepts and slopes to regularize all parameters and minimize the risk of overfitting. The final results are a combination of these two Bayesian models.

To address missing data, the GDD employed a Bayesian hierarchical framework with several integrated mechanisms. First, through partial pooling, countries with sparse or no survey data borrowed statistical strength from other countries within their world region, with estimates shrinking toward regional means proportionally to data availability—less informative data resulted in greater pooling toward regional estimates. Second, for country-year covariates with intermittent missing values, linear interpolation was used; covariates ending before 2018 were imputed using a three-year moving average; and region-level means were assigned when entire covariates were missing for a country. Third, for countries without survey data, posterior distributions of mean intake were generated by sampling from the corresponding world region-level parameters (intercepts, sex effects, age patterns, education effects, and urban-rural differences) combined with their respective between-country variance distributions.

In the GDD 2018, global, regional, national, and within country population subgroup estimated intakes of tea and their uncertainty were calculated as population weighted averages using all 4000 posterior predictions for each of the 264 demographic strata in each country-year^41^. Population weights for each year were derived from the United Nations Population Division, supplemented with data for education and urban or rural status from Barro and Lee and the United Nations^41^. For the GDD estimates, UIs are derived from a Bayesian model and can be interpreted as at least 95% probability that the true mean is contained within the interval.

### Tea intake definition and data extraction

Tea intake was defined as total green or black tea consumption, including caffeinated, decaffeinated, sweetened, or unsweetened tea, excluding herbal teas. All intakes were standardized as 8-oz cups per week. Using standardized methods, we extracted tea intake data stratified by age, sex, education level (≤6 years, >6-12 years, >12 years), and urbanicity (urban vs rural).

### Statistical analysis

A descriptive analysis was performed to characterise the patterns of estimated tea intake among adults aged ≥20 years on a global scale. We compared estimated tea intake (cup (8oz) per week) across different age groups, sexes, education levels, areas of residence, regions, and countries.

To quantitatively evaluate the temporal trends in estimated tea intake, we calculated EAPC for the periods 1990–2005, 2005–2018, and 1990–2018. Specifically, to calculate EAPCs and obtain 95% CIs, the regression line was fitted to the natural logarithm of the tea intake rate, i.e., *y* = *α* + *β*x + *ε*, where *y* = ln(tea intake) and *x* = calendar year, and EAPC = 100 × (*e*^β^ – 1)^42,43^. EAPC values > 0 indicate an increase in tea intake over time, and EAPC values < 0 indicate a decrease over time. An EAPC value with a 95% CI that includes 0 indicates stability, or no significant change in estimated tea intake during the indicated time period.

We assessed national estimated intakes of tea and trends by SDI, including trends over time between 1990 and 2005, 2005 and 2018, and 1990 and 2018. Spearman correlation coefficients were calculated to evaluate associations between estimated tea intake and SDI across the 185 countries for each time point, with 95% CIs estimated using bootstrap methods. The SDI is a measure of the development of a country or region, ranging from 0 to 1, with 0 representing the minimum level and 1 the maximum level of development of a given nation, and it is based on income per capita, average educational attainment, and fertility rates ^44^.

To assess the potential influence of population size on regional estimates, we also calculated equally-weighted regional averages as a sensitivity analysis. Equally-weighted estimates were calculated using the simple arithmetic mean of country-level estimates within each region.

All statistical analyses were conducted using R (version 4.2.3).

## Supplementary information

Supplementary information

## Data Availability

The data used for analyses are available freely online at the GDD (https://www.globaldietarydatabase.org/data-download).
