# Supplementary material for: Tea intake among adults in 185 countries between 1990 and 2018: population based study
Source: NPJ Sci Food. 2026 Apr 10;10:185. doi: 10.1038/s41538-026-00817-4 (PMC13254342; doi:10.1038/s41538-026-00817-4)
Supplement: Supplementary file 1 — Supplementary information [file 41538_2026_817_MOESM1_ESM.docx]

**Supplementary Information | Tea intake among adults in 185 countries between 1990 and 2018: population based study**

**Table of Contents**

**Supplementary Table 1.** Tea intakes (cup (8 oz) per week) in 1990, 2005, and 2018 and EAPC from 1990-2005, 2005-2018, and 1990-2018 in adults aged ≥20 years globally, regionally, and nationally.

**Supplementary Table 2.** National mean tea intake (cup (8 oz) per week) in adults aged ≥20 years by sex, age, parental education, and area of residence in the 25 most populous countries in 2018.

**Supplementary Table 3.** Mean tea intake (cup (8 oz) per week) in adults aged ≥20 years by age, education, and area of residence, presented by global and regional estimates stratified by sex across 185 countries in 2018.

**Supplementary Table 4.** National mean tea intake (cup (8 oz) per week) in adults aged ≥20 years by age, education, and area of residence, presented by global and regional estimates stratified by sex across in the 25 most populous countries in 2018.

**Supplementary Table 5.** Mean tea intake (cup (8 oz) per week) in adults aged ≥20 years by sex, age, and area of residence, presented by global and regional estimates stratified by education level across 185 countries in 2018.

**Supplementary Table 6.** National mean tea intake (cup (8 oz) per week) in adults aged ≥20 years by sex, age, and area of residence, presented by global and regional estimates stratified by education level across in the 25 most populous countries in 2018.

**Supplementary Table 7.** Mean tea intake (cup (8 oz) per week) in adults aged ≥20 years by sex, age, and education, presented by global and regional estimates stratified by area of residence across 185 countries in 2018.

**Supplementary Table 8.** National mean tea intake (cup (8 oz) per week) in adults aged ≥20 years by sex, age, and education, presented by global and regional estimates stratified by area of residence across in the 25 most populous countries in 2018.

**Supplementary Table 9.** Global and regional mean tea intake (cup (8 oz) per week) in adults aged ≥20 years, by age, sex, education, and area of residence across 185 countries in 1990.

**Supplementary Table 10.** Global and regional mean tea intake (cup (8 oz) per week) in adults aged ≥20 years, by age, sex, education, and area of residence across 185 countries in 2005.

**Supplementary Table 11.** National mean tea intake (cup (8 oz) per week) in adults aged ≥20 years by sex, age, parental education, and area of residence in the 25 most populous countries in 1990.

**Supplementary Table 12.** National mean tea intake (cup (8 oz) per week) in adults aged ≥20 years by sex, age, parental education, and area of residence in the 25 most populous countries in 2005.

**Supplementary Table 13.** Global and regional EAPC in mean tea intake (cup (8 oz) per week) from 1990-2005, 2005-2018, and 1990-2018 in adults aged ≥20 years, by sex, age, education, and area of residence across 185 countries.

**Supplementary Table 14.** Equally weighted regional tea intakes (cup (8 oz) per week) in 1990, 2005, and 2018 and EAPC from 1990-2005, 2005-2018, and 1990-2018 in adults aged ≥20 years.

**Supplementary Figure 1.** Flow chart of data extraction and analysis process from the Global Dietary Database 2018.

**Supplementary Figure 2.** Global and regional intakes of tea (cup (8 oz) per week) by age among adults aged ≥20 years in females and males in 2018.

**Supplementary Figure 3.** National mean intakes of tea (cup (8 oz) per week) in adults aged ≥20 years across 185 countries in 1990 and 2005.

**Supplementary Figure 4.** Global and regional intakes of tea (cup (8 oz) per week) by age in adults aged ≥20 years in 1990 and 2005.

**Supplementary Figure 5.** Global and regional mean tea intakes (cup (8 oz) per week) in adults aged ≥20 years by area of residence in 1990 and 2005.

**Supplementary Figure 6.** Global and regional mean tea intakes (cup (8 oz) per week) in adults aged ≥20 years by area of residence and education level in 1990 and 2005.

**Supplementary Figure 7.** National correlation of tea intake (cup (8 oz) per week) in adults aged ≥20 years and socio-demographic development index by world region in 2005 for 185 countries.

**Supplementary Table 1. Tea intakes (cup (8 oz) per week) in 1990, 2005, and 2018 and EAPC from 1990-2005, 2005-2018, and 1990-2018 in adults aged ≥20 years globally, regionally, and nationally.**

|  | **Mean intake (95% UI)** | | | **EAPC (95% CI)** | | |
| --- | --- | --- | --- | --- | --- | --- |
|  | **1990** | **2005** | **2018** | **1990-2005** | **2005-2018** | **1990-2018** |
| **Worldwide** | 4.82 (4.51 to 5.21) | 6.06 (5.18 to 7.19) | 6.18 (5.66 to 6.82) | 0.75 (0.46 to 1.05) | 1.02 (0.71 to 1.34) | 0.94 (0.81 to 1.08) |
| **Region** |  |  |  |  |  |  |
| Central and eastern Europe and central Asia* | 4.13 (3.49 to 4.93) | 6.16 (5.23 to 7.38) | 5.45 (4.66 to 6.45) | 2.47 (0.36 to 4.63) | -1.34 (-3.15 to 0.50) | 0.65 (-0.53 to 1.85) |
| High income countries | 4.73 (4.38 to 5.16) | 4.93 (4.56 to 5.37) | 4.88 (4.53 to 5.31) | 0.28 (0.15 to 0.41) | -0.08 (-0.29 to 0.12) | 0.10 (0.01 to 0.19) |
| Latin America and the Caribbean | 3.71 (3.40 to 4.09) | 3.83 (3.51 to 4.21) | 3.85 (3.52 to 4.25) | 0.22 (-0.01 to 0.46) | 0.06 (0.00 to 0.11) | 0.10 (0.01 to 0.18) |
| Middle East and north Africa | 9.48 (8.43 to 10.87) | 8.23 (7.21 to 9.58) | 8.95 (7.94 to 10.29) | -0.87 (-1.15 to -0.60) | 0.59 (-0.70 to 1.89) | -0.02 (-0.44 to 0.41) |
| South Asia* | 4.44 (4.02 to 4.92) | 4.91 (4.45 to 5.42) | 5.46 (4.93 to 6.07) | 0.57 (0.14 to 1.00) | 1.12 (-0.02 to 2.28) | 0.76 (0.42 to 1.10) |
| Southeast and east Asia | 4.98 (4.27 to 5.90) | 4.00 (3.74 to 4.30) | 7.65 (6.32 to 9.38) | 1.22 (0.62 to 1.82) | 1.84 (1.41 to 2.28) | 1.68 (1.41 to 1.96) |
| Sub-Saharan Africa | 4.08 (3.80 to 4.38) | 5.45 (5.07 to 5.91) | 4.94 (4.58 to 5.37) | -0.19 (-0.56 to 0.18) | 1.90 (0.94 to 2.87) | 0.77 (0.22 to 1.33) |
| **Country** |  |  |  |  |  |  |
| Afghanistan | 9.23 (6.27 to 13.82) | 5.17 (3.66 to 7.39) | 13.74 (9.40 to 20.41) | -3.53 (-6.35 to -0.63) | 8.27 (4.64 to 12.02) | 1.66 (-0.86 to 4.25) |
| Angola | 4.04 (3.14 to 5.26) | 4.09 (3.24 to 5.25) | 4.08 (3.19 to 5.35) | 0.08 (0.07 to 0.10) | -0.03 (-0.07 to 0.01) | 0.03 (0.01 to 0.06) |
| Albania | 5.07 (3.77 to 6.83) | 4.96 (3.65 to 6.75) | 5.00 (3.74 to 6.71) | -0.17 (-0.32 to -0.02) | 0.37 (-0.73 to 1.47) | 0.07 (-0.23 to 0.37) |
| United Arab Emirates | 6.81 (4.76 to 9.92) | 7.54 (5.25 to 10.94) | 6.45 (4.50 to 9.49) | 0.95 (-2.46 to 4.49) | -0.94 (-3.15 to 1.31) | -0.98 (-2.35 to 0.41) |
| Argentina | 4.05 (3.68 to 4.48) | 4.15 (3.76 to 4.59) | 4.11 (3.73 to 4.55) | 0.16 (-0.09 to 0.42) | -0.07 (-0.17 to 0.03) | -0.01 (-0.11 to 0.09) |
| Armenia | 4.89 (3.50 to 6.84) | 4.84 (3.44 to 6.85) | 4.86 (3.50 to 6.80) | -0.07 (-0.08 to -0.05) | 0.04 (0.01 to 0.06) | -0.02 (-0.04 to 0.00) |
| Antigua and Barbuda | 1.67 (1.23 to 2.29) | 4.59 (3.39 to 6.30) | 4.62 (3.39 to 6.38) | 7.40 (3.84 to 11.09) | 0.04 (-5.57 to 5.99) | 3.36 (1.26 to 5.51) |
| Australia | 6.42 (4.54 to 9.38) | 5.32 (3.73 to 7.83) | 3.59 (2.54 to 5.22) | -1.02 (-2.25 to 0.22) | -2.41 (-4.79 to 0.03) | -1.47 (-2.24 to -0.70) |
| Austria | 1.62 (1.22 to 2.18) | 5.80 (4.32 to 7.85) | 2.73 (2.07 to 3.64) | 8.33 (0.94 to 16.26) | -4.29 (-14.33 to 6.92) | 1.83 (-1.77 to 5.56) |
| Azerbaijan | 6.78 (6.16 to 7.47) | 5.37 (4.90 to 5.86) | 8.19 (7.35 to 9.14) | -1.96 (-3.73 to -0.16) | 2.54 (-3.97 to 9.49) | -0.47 (-2.51 to 1.60) |
| Burundi | 4.72 (3.32 to 6.99) | 4.02 (2.96 to 5.61) | 7.04 (5.05 to 10.14) | -0.78 (-2.69 to 1.17) | 3.89 (1.01 to 6.86) | 2.00 (0.62 to 3.40) |
| Belgium | 8.30 (5.89 to 11.66) | 5.42 (3.85 to 7.63) | 7.30 (5.18 to 10.26) | -3.56 (-6.80 to -0.21) | 3.01 (-0.03 to 6.13) | -0.48 (-2.46 to 1.54) |
| Benin | 3.99 (3.00 to 5.40) | 4.04 (3.15 to 5.27) | 4.01 (3.04 to 5.38) | 0.08 (0.07 to 0.10) | -0.08 (-0.18 to 0.02) | 0.02 (-0.02 to 0.07) |
| Burkina Faso | 3.63 (3.32 to 3.98) | 3.65 (3.35 to 3.99) | 5.42 (4.95 to 5.94) | 0.04 (0.02 to 0.06) | 3.55 (1.54 to 5.61) | 1.41 (0.46 to 2.38) |
| Bangladesh | 3.36 (3.09 to 3.66) | 3.97 (3.66 to 4.32) | 4.45 (4.08 to 4.86) | 1.13 (1.06 to 1.20) | 0.99 (0.38 to 1.60) | 1.13 (0.96 to 1.29) |
| Bulgaria | 5.23 (4.38 to 6.26) | 5.13 (4.30 to 6.13) | 5.18 (4.34 to 6.20) | -0.12 (-0.16 to -0.09) | 0.11 (-0.05 to 0.27) | -0.04 (-0.11 to 0.03) |
| Bahrain | 7.52 (5.25 to 10.88) | 7.46 (5.23 to 10.84) | 7.50 (5.18 to 10.96) | 0.01 (-0.34 to 0.36) | -0.15 (-1.39 to 1.11) | 0.15 (-0.20 to 0.50) |
| The Bahamas | 4.75 (3.49 to 6.60) | 4.46 (3.26 to 6.21) | 3.69 (2.69 to 5.16) | 0.53 (-7.31 to 9.03) | 0.26 (-6.69 to 7.73) | 1.74 (-1.25 to 4.83) |
| Bosnia and Herzegovina | 5.05 (3.75 to 6.79) | 4.92 (3.60 to 6.70) | 4.98 (3.71 to 6.68) | -0.17 (-0.20 to -0.14) | 0.14 (-0.03 to 0.30) | -0.04 (-0.12 to 0.04) |
| Belarus | 3.68 (2.72 to 4.99) | 5.09 (3.63 to 7.19) | 6.08 (4.43 to 8.37) | 2.06 (0.19 to 3.97) | 1.09 (-0.84 to 3.05) | 2.28 (1.43 to 3.13) |
| Belize | 3.72 (2.69 to 5.23) | 4.02 (2.93 to 5.61) | 4.85 (3.50 to 6.80) | -0.08 (-2.54 to 2.44) | 0.29 (-3.62 to 4.37) | 0.41 (-0.81 to 1.65) |
| Bolivia | 3.94 (2.96 to 5.33) | 3.93 (2.92 to 5.36) | 4.66 (3.46 to 6.34) | 0.07 (-0.40 to 0.55) | 1.28 (0.90 to 1.67) | 0.62 (0.35 to 0.89) |
| Brazil | 3.41 (3.15 to 3.68) | 3.65 (3.40 to 3.93) | 3.66 (3.40 to 3.94) | 0.52 (0.02 to 1.02) | 0.04 (-0.03 to 0.11) | 0.19 (-0.01 to 0.39) |
| Barbados | 3.00 (2.20 to 4.16) | 4.49 (3.24 to 6.33) | 4.79 (3.48 to 6.67) | 1.61 (-5.86 to 9.68) | 1.04 (-1.76 to 3.92) | 2.44 (0.22 to 4.70) |
| Brunei | 2.87 (2.19 to 3.78) | 5.18 (3.92 to 6.88) | 4.77 (3.60 to 6.38) | 4.81 (-0.62 to 10.54) | -0.70 (-1.07 to -0.32) | 1.20 (-0.94 to 3.38) |
| Bhutan | 5.81 (4.10 to 8.40) | 5.41 (3.95 to 7.42) | 7.92 (5.70 to 11.09) | -0.45 (-0.62 to -0.27) | 3.36 (2.01 to 4.72) | 1.27 (0.38 to 2.16) |
| Botswana | 11.42 (9.08 to 14.50) | 4.16 (3.29 to 5.33) | 8.28 (6.57 to 10.51) | -5.53 (-11.80 to 1.18) | 5.04 (-2.66 to 13.36) | -0.79 (-3.65 to 2.15) |
| Central African Republic | 3.63 (3.36 to 3.93) | 3.66 (3.39 to 3.95) | 3.63 (3.36 to 3.93) | 0.05 (0.05 to 0.05) | -0.07 (-0.10 to -0.03) | 0.00 (-0.03 to 0.03) |
| Canada | 4.89 (4.66 to 5.12) | 4.92 (4.69 to 5.16) | 4.70 (4.48 to 4.93) | 0.30 (-0.78 to 1.39) | -0.33 (-0.61 to -0.06) | -0.14 (-0.50 to 0.22) |
| Switzerland | 5.63 (4.83 to 6.58) | 5.72 (4.91 to 6.69) | 5.67 (4.87 to 6.64) | 0.06 (-0.26 to 0.38) | -0.07 (-0.15 to 0.00) | 0.05 (-0.05 to 0.14) |
| Chile | 4.39 (3.22 to 6.10) | 4.62 (3.33 to 6.55) | 4.60 (3.34 to 6.39) | 0.04 (-1.36 to 1.47) | 0.02 (-0.78 to 0.82) | 0.37 (-0.12 to 0.86) |
| China | 3.79 (2.87 to 5.03) | 4.90 (3.74 to 6.47) | 8.04 (6.11 to 10.55) | 1.58 (0.67 to 2.50) | 3.95 (2.67 to 5.25) | 3.04 (2.33 to 3.76) |
| Cote d'Ivoire | 3.68 (3.44 to 3.95) | 3.74 (3.50 to 4.01) | 3.86 (3.61 to 4.15) | 0.22 (-0.32 to 0.76) | 0.22 (-0.00 to 0.44) | 0.27 (0.10 to 0.43) |
| Cameroon | 3.16 (2.95 to 3.40) | 3.50 (3.27 to 3.75) | 5.69 (5.29 to 6.14) | 0.62 (-0.74 to 1.98) | 3.89 (1.22 to 6.63) | 1.50 (0.38 to 2.63) |
| Democratic Republic of the Congo | 4.00 (3.04 to 5.37) | 4.02 (3.10 to 5.33) | 4.02 (3.10 to 5.32) | 0.04 (0.01 to 0.07) | -0.01 (-0.07 to 0.05) | 0.02 (0.00 to 0.04) |
| Congo | 4.08 (3.23 to 5.22) | 4.09 (3.21 to 5.27) | 4.11 (3.23 to 5.31) | 0.01 (-0.00 to 0.03) | 0.04 (0.03 to 0.05) | 0.03 (0.02 to 0.03) |
| Colombia | 3.83 (3.46 to 4.26) | 3.84 (3.46 to 4.28) | 3.83 (3.44 to 4.27) | 0.02 (-0.00 to 0.03) | -0.02 (-0.04 to -0.00) | -0.01 (-0.02 to 0.00) |
| Comoros | 4.86 (4.42 to 5.33) | 3.97 (3.61 to 4.36) | 7.72 (6.98 to 8.50) | -1.00 (-3.27 to 1.31) | 4.65 (1.26 to 8.16) | 2.34 (0.68 to 4.02) |
| Cape Verde | 4.02 (3.07 to 5.34) | 4.07 (3.16 to 5.31) | 4.06 (3.11 to 5.46) | 0.08 (0.07 to 0.09) | -0.04 (-0.13 to 0.05) | 0.03 (-0.00 to 0.07) |
| Costa Rica | 3.95 (2.95 to 5.35) | 3.94 (2.93 to 5.38) | 5.15 (3.79 to 7.13) | -0.02 (-0.03 to 0.00) | 1.65 (-0.71 to 4.06) | 0.54 (-0.18 to 1.26) |
| Cuba | 3.92 (2.91 to 5.43) | 3.93 (2.88 to 5.45) | 3.91 (2.86 to 5.42) | 0.02 (-0.01 to 0.05) | -0.04 (-0.05 to -0.03) | -0.02 (-0.04 to 0.00) |
| Cyprus | 3.90 (2.95 to 5.23) | 4.84 (3.66 to 6.46) | 6.38 (4.81 to 8.55) | 1.35 (0.14 to 2.58) | 1.97 (-0.07 to 4.05) | 2.22 (1.49 to 2.95) |
| Czech Republic | 4.76 (3.50 to 6.48) | 5.00 (3.51 to 7.15) | 4.11 (3.03 to 5.60) | 0.09 (-1.10 to 1.30) | -1.73 (-2.77 to -0.68) | -0.88 (-1.47 to -0.30) |
| Germany | 4.06 (3.81 to 4.31) | 4.61 (4.31 to 4.93) | 4.41 (4.15 to 4.68) | 0.77 (0.14 to 1.40) | -0.32 (-0.66 to 0.02) | 0.32 (0.06 to 0.58) |
| Djibouti | 6.35 (4.79 to 8.59) | 4.40 (3.39 to 5.82) | 10.02 (7.50 to 13.69) | -0.89 (-7.52 to 6.22) | 2.14 (-12.27 to 18.93) | 1.78 (-2.47 to 6.22) |
| Dominica | 3.44 (2.54 to 4.68) | 4.01 (2.98 to 5.49) | 7.60 (5.62 to 10.41) | 0.93 (-0.09 to 1.96) | 3.83 (-3.67 to 11.92) | 1.66 (-0.39 to 3.75) |
| Denmark | 4.28 (3.13 to 5.91) | 5.31 (3.82 to 7.48) | 6.52 (4.74 to 9.08) | 1.36 (-0.15 to 2.91) | 1.04 (-4.64 to 7.07) | 0.73 (-0.71 to 2.20) |
| Dominican Republic | 3.85 (3.48 to 4.28) | 3.86 (3.47 to 4.29) | 3.85 (3.46 to 4.30) | 0.02 (-0.00 to 0.03) | -0.02 (-0.04 to -0.01) | -0.00 (-0.01 to 0.01) |
| Algeria | 6.31 (4.50 to 8.99) | 6.80 (4.88 to 9.60) | 6.62 (4.69 to 9.51) | 0.50 (-2.49 to 3.58) | -0.08 (-0.96 to 0.80) | 0.88 (-0.06 to 1.83) |
| Ecuador | 3.95 (2.96 to 5.33) | 3.94 (2.95 to 5.34) | 3.94 (2.95 to 5.35) | -0.02 (-0.03 to 0.00) | 0.01 (-0.02 to 0.04) | -0.01 (-0.02 to 0.00) |
| Egypt | 11.57 (10.42 to 12.82) | 4.68 (4.32 to 5.07) | 9.66 (8.77 to 10.63) | -5.50 (-10.32 to -0.43) | 5.53 (-1.32 to 12.86) | -0.43 (-3.12 to 2.32) |
| Eritrea | 4.92 (3.69 to 6.71) | 4.24 (3.31 to 5.52) | 7.47 (5.74 to 9.88) | -0.66 (-2.50 to 1.21) | 3.97 (1.19 to 6.83) | 2.03 (0.70 to 3.38) |
| Spain | 4.67 (3.52 to 6.28) | 4.78 (3.57 to 6.50) | 4.76 (3.57 to 6.47) | 0.15 (0.12 to 0.18) | -0.06 (-0.17 to 0.05) | 0.07 (0.01 to 0.13) |
| Estonia | 3.07 (2.24 to 4.22) | 5.22 (3.75 to 7.33) | 4.02 (2.94 to 5.51) | 3.70 (0.98 to 6.50) | -1.85 (-3.27 to -0.41) | 0.74 (-0.86 to 2.36) |
| Ethiopia | 4.19 (3.95 to 4.45) | 4.22 (3.98 to 4.48) | 4.19 (3.95 to 4.46) | 0.05 (0.05 to 0.05) | -0.06 (-0.12 to -0.00) | 0.00 (-0.03 to 0.03) |
| Finland | 2.96 (2.19 to 4.08) | 5.26 (3.86 to 7.30) | 3.52 (2.57 to 4.88) | 3.52 (-0.57 to 7.77) | -2.93 (-5.49 to -0.30) | 0.81 (-0.79 to 2.44) |
| Fiji | 5.20 (3.96 to 6.85) | 5.06 (3.85 to 6.63) | 4.81 (3.68 to 6.30) | -0.06 (-0.58 to 0.46) | -0.38 (-0.64 to -0.11) | -0.28 (-0.46 to -0.10) |
| France | 4.22 (3.18 to 5.65) | 4.97 (3.66 to 6.84) | 5.04 (3.78 to 6.78) | 1.34 (0.32 to 2.37) | 0.07 (-0.14 to 0.29) | 0.66 (0.24 to 1.09) |
| Federated States of Micronesia | 3.67 (2.77 to 4.92) | 5.19 (3.89 to 7.02) | 2.67 (2.01 to 3.59) | 1.83 (-0.71 to 4.44) | -3.02 (-11.56 to 6.35) | -1.26 (-3.61 to 1.14) |
| Gabon | 4.13 (3.24 to 5.35) | 4.19 (3.26 to 5.44) | 4.15 (3.15 to 5.54) | 0.09 (0.07 to 0.12) | -0.10 (-0.20 to -0.00) | 0.01 (-0.04 to 0.06) |
| United Kingdom | 6.31 (4.58 to 8.80) | 5.87 (4.28 to 8.12) | 4.89 (3.54 to 6.91) | -0.47 (-0.51 to -0.43) | -1.36 (-1.64 to -1.08) | -0.86 (-1.07 to -0.64) |
| Georgia | 9.96 (7.22 to 13.81) | 6.26 (4.47 to 8.80) | 2.61 (1.92 to 3.53) | -3.47 (-5.86 to -1.01) | -6.95 (-14.96 to 1.81) | -5.91 (-8.03 to -3.75) |
| Ghana | 3.56 (3.31 to 3.83) | 5.70 (5.27 to 6.17) | 3.60 (3.35 to 3.88) | 2.88 (-0.28 to 6.13) | -3.23 (-6.27 to -0.10) | 0.06 (-1.41 to 1.56) |
| Guinea | 3.63 (3.37 to 3.93) | 3.66 (3.40 to 3.96) | 3.93 (3.65 to 4.25) | 0.05 (0.05 to 0.05) | -0.31 (-5.38 to 5.03) | 0.68 (-0.67 to 2.06) |
| The Gambia | 4.59 (3.42 to 6.28) | 4.92 (3.78 to 6.52) | 4.71 (3.51 to 6.46) | 1.82 (-6.27 to 10.61) | -1.14 (-4.51 to 2.36) | 1.53 (-1.09 to 4.22) |
| Guinea-Bissau | 3.99 (3.03 to 5.39) | 4.03 (3.13 to 5.27) | 5.34 (4.10 to 7.10) | 0.06 (0.05 to 0.08) | 5.28 (-5.39 to 17.16) | 2.44 (-0.45 to 5.42) |
| Equatorial Guinea | 4.03 (3.14 to 5.28) | 4.10 (3.24 to 5.23) | 4.10 (3.16 to 5.42) | 0.11 (0.10 to 0.13) | -0.01 (-0.06 to 0.03) | 0.06 (0.02 to 0.09) |
| Greece | 4.76 (3.59 to 6.38) | 4.81 (3.57 to 6.57) | 14.14 (10.52 to 19.16) | 0.06 (0.01 to 0.11) | 7.36 (1.79 to 13.23) | 3.08 (0.95 to 5.25) |
| Grenada | 3.00 (2.22 to 4.11) | 4.12 (3.06 to 5.59) | 3.01 (2.22 to 4.10) | 1.92 (-0.21 to 4.10) | -2.22 (-4.38 to -0.01) | 0.01 (-0.99 to 1.03) |
| Guatemala | 3.94 (2.87 to 5.51) | 3.94 (2.88 to 5.45) | 3.93 (2.86 to 5.50) | -0.01 (-0.03 to 0.02) | -0.02 (-0.04 to -0.01) | -0.01 (-0.02 to 0.00) |
| Guyana | 3.51 (3.12 to 3.96) | 3.59 (3.19 to 4.04) | 3.83 (3.43 to 4.28) | 0.31 (-0.50 to 1.14) | 0.49 (0.27 to 0.71) | 0.29 (0.07 to 0.52) |
| Honduras | 3.94 (2.92 to 5.39) | 3.95 (2.96 to 5.36) | 3.96 (2.93 to 5.42) | 0.02 (-0.00 to 0.03) | 0.02 (-0.01 to 0.04) | 0.01 (0.00 to 0.02) |
| Croatia | 5.03 (3.74 to 6.75) | 4.91 (3.62 to 6.65) | 4.98 (3.70 to 6.70) | -0.16 (-0.16 to -0.16) | 0.14 (-0.02 to 0.31) | -0.04 (-0.13 to 0.04) |
| Haiti | 3.86 (3.49 to 4.29) | 3.86 (3.49 to 4.29) | 3.87 (3.49 to 4.29) | 0.00 (0.00 to 0.00) | 0.02 (-0.01 to 0.04) | 0.01 (-0.00 to 0.01) |
| Hungary | 4.53 (3.37 to 6.11) | 4.94 (3.58 to 6.84) | 4.50 (3.34 to 6.09) | 0.61 (0.01 to 1.20) | -0.58 (-1.66 to 0.51) | -0.03 (-0.38 to 0.32) |
| Indonesia | 3.81 (3.59 to 4.06) | 4.04 (3.80 to 4.31) | 4.21 (3.95 to 4.51) | 0.24 (-0.56 to 1.05) | 0.35 (0.16 to 0.54) | 0.22 (-0.01 to 0.46) |
| India | 4.28 (3.87 to 4.74) | 4.90 (4.43 to 5.42) | 5.47 (4.93 to 6.08) | 0.78 (0.23 to 1.32) | 1.17 (-0.07 to 2.42) | 0.88 (0.52 to 1.24) |
| Ireland | 6.92 (5.16 to 9.31) | 5.86 (4.34 to 7.98) | 4.73 (3.54 to 6.37) | -1.10 (-1.36 to -0.83) | -1.91 (-2.88 to -0.94) | -1.48 (-1.80 to -1.16) |
| Iran | 16.26 (15.35 to 17.21) | 16.01 (15.10 to 16.94) | 17.46 (16.55 to 18.50) | -0.20 (-0.65 to 0.24) | 0.73 (0.16 to 1.29) | 0.37 (0.08 to 0.66) |
| Iraq | 10.78 (7.64 to 15.48) | 7.57 (5.42 to 10.73) | 4.50 (3.20 to 6.43) | -1.95 (-7.09 to 3.47) | -3.79 (-6.82 to -0.65) | -2.54 (-4.07 to -0.98) |
| Iceland | 3.44 (2.51 to 4.82) | 5.15 (3.72 to 7.22) | 5.11 (3.70 to 7.17) | 3.06 (1.37 to 4.76) | -0.88 (-3.81 to 2.14) | 1.24 (0.03 to 2.47) |
| Israel | 10.16 (9.51 to 10.83) | 9.17 (8.63 to 9.74) | 9.21 (8.67 to 9.78) | -0.66 (-0.86 to -0.45) | 0.01 (-0.15 to 0.18) | -0.36 (-0.50 to -0.21) |
| Italy | 3.66 (2.91 to 4.60) | 3.71 (2.95 to 4.66) | 3.73 (2.97 to 4.69) | 0.09 (0.07 to 0.11) | 0.04 (0.00 to 0.08) | 0.08 (0.06 to 0.09) |
| Jamaica | 3.92 (2.92 to 5.33) | 3.94 (2.93 to 5.37) | 3.95 (2.95 to 5.36) | 0.04 (0.02 to 0.05) | 0.02 (0.01 to 0.04) | 0.02 (0.02 to 0.03) |
| Jordan | 9.69 (6.91 to 13.72) | 7.45 (5.26 to 10.72) | 6.31 (4.42 to 9.13) | -1.56 (-2.56 to -0.55) | -1.31 (-1.79 to -0.82) | -1.44 (-1.75 to -1.13) |
| Japan | 18.54 (15.81 to 21.77) | 22.43 (19.15 to 26.38) | 14.95 (12.74 to 17.54) | 1.32 (1.14 to 1.50) | -3.14 (-3.83 to -2.44) | -0.91 (-1.89 to 0.08) |
| Kazakhstan | 4.35 (3.92 to 4.81) | 5.19 (4.72 to 5.72) | 5.38 (4.90 to 5.91) | 1.14 (0.47 to 1.81) | 0.34 (0.09 to 0.60) | 0.67 (0.39 to 0.96) |
| Kenya | 8.37 (7.60 to 9.23) | 3.78 (3.50 to 4.10) | 13.09 (11.66 to 14.72) | -5.51 (-7.99 to -2.96) | 11.90 (4.50 to 19.83) | 2.00 (-2.38 to 6.57) |
| Kyrgyzstan | 4.75 (4.25 to 5.30) | 7.73 (6.98 to 8.57) | 8.04 (7.26 to 8.90) | 3.95 (1.27 to 6.71) | 0.12 (-0.78 to 1.03) | 1.87 (0.69 to 3.06) |
| Cambodia | 3.42 (3.12 to 3.79) | 3.42 (3.12 to 3.79) | 3.43 (3.12 to 3.80) | 0.00 (-0.04 to 0.04) | 0.03 (0.01 to 0.04) | 0.00 (-0.01 to 0.02) |
| Kiribati | 3.69 (2.80 to 4.89) | 5.23 (3.96 to 6.92) | 2.72 (2.08 to 3.59) | 1.85 (-0.69 to 4.46) | -2.92 (-11.51 to 6.51) | -1.20 (-3.56 to 1.21) |
| South Korea | 3.48 (3.38 to 3.58) | 3.48 (3.38 to 3.58) | 3.47 (3.37 to 3.57) | 0.00 (0.00 to 0.00) | -0.02 (-0.04 to -0.00) | -0.01 (-0.02 to -0.01) |
| Kuwait | 7.63 (5.21 to 11.29) | 7.54 (5.11 to 11.16) | 7.04 (4.77 to 10.42) | -0.36 (-3.91 to 3.32) | -1.77 (-8.20 to 5.10) | -0.56 (-2.49 to 1.40) |
| Laos | 6.75 (4.92 to 9.42) | 4.53 (3.41 to 6.07) | 11.92 (8.86 to 16.16) | -2.36 (-4.99 to 0.34) | 9.11 (3.53 to 14.99) | 2.58 (-0.48 to 5.74) |
| Lebanon | 8.42 (7.53 to 9.37) | 6.91 (6.19 to 7.69) | 7.50 (6.71 to 8.35) | -1.11 (-2.08 to -0.13) | 0.82 (0.04 to 1.60) | -0.14 (-0.68 to 0.40) |
| Liberia | 3.67 (3.40 to 3.96) | 3.57 (3.31 to 3.85) | 3.56 (3.31 to 3.85) | -0.16 (-0.40 to 0.08) | -0.03 (-0.08 to 0.02) | -0.06 (-0.14 to 0.02) |
| Libya | 7.56 (5.36 to 10.80) | 7.51 (5.34 to 10.66) | 7.47 (5.29 to 10.74) | 0.02 (-0.35 to 0.39) | -0.25 (-1.53 to 1.04) | 0.11 (-0.25 to 0.48) |
| Saint Lucia | 2.85 (2.10 to 3.91) | 4.16 (3.07 to 5.71) | 2.86 (2.08 to 3.99) | 2.69 (2.11 to 3.27) | -2.64 (-5.16 to -0.05) | -0.34 (-1.64 to 0.98) |
| Sri Lanka | 6.23 (5.18 to 7.44) | 6.56 (5.46 to 7.83) | 3.11 (2.55 to 3.77) | 0.28 (-0.63 to 1.19) | -4.48 (-13.49 to 5.47) | -0.90 (-3.66 to 1.94) |
| Lesotho | 5.75 (4.31 to 7.95) | 4.06 (3.17 to 5.29) | 4.56 (3.51 to 6.06) | -2.82 (-4.97 to -0.62) | 0.92 (0.26 to 1.60) | -1.03 (-2.11 to 0.06) |
| Lithuania | 3.51 (2.60 to 4.78) | 5.14 (3.69 to 7.19) | 4.67 (3.44 to 6.34) | 3.52 (-0.55 to 7.76) | -0.73 (-0.88 to -0.58) | 0.94 (-0.63 to 2.54) |
| Luxembourg | 6.74 (5.00 to 9.23) | 5.05 (3.71 to 7.00) | 7.34 (5.35 to 10.18) | -2.34 (-4.24 to -0.41) | 2.75 (0.69 to 4.86) | 0.62 (-0.78 to 2.04) |
| Latvia | 3.27 (2.41 to 4.45) | 5.23 (3.80 to 7.20) | 3.82 (2.81 to 5.17) | 4.10 (0.28 to 8.06) | -2.23 (-3.09 to -1.37) | 0.74 (-1.10 to 2.62) |
| Morocco | 6.45 (4.53 to 9.35) | 7.84 (5.60 to 11.25) | 8.37 (5.83 to 12.21) | 1.23 (0.88 to 1.59) | 0.53 (0.44 to 0.61) | 0.89 (0.71 to 1.08) |
| Moldova | 4.39 (3.25 to 5.89) | 5.01 (3.64 to 6.95) | 4.95 (3.68 to 6.65) | 0.78 (-0.26 to 1.83) | -0.18 (-0.50 to 0.14) | 0.54 (0.19 to 0.88) |
| Madagascar | 3.74 (3.50 to 4.01) | 3.77 (3.53 to 4.04) | 3.77 (3.52 to 4.04) | 0.05 (0.05 to 0.05) | -0.01 (-0.07 to 0.05) | 0.03 (0.01 to 0.06) |
| Maldives | 4.70 (4.26 to 5.19) | 4.38 (3.97 to 4.84) | 4.93 (4.46 to 5.44) | -0.38 (-0.81 to 0.04) | 0.95 (0.73 to 1.16) | 0.13 (-0.18 to 0.45) |
| Mexico | 3.53 (3.18 to 3.91) | 3.53 (3.18 to 3.92) | 3.53 (3.18 to 3.92) | 0.00 (0.00 to 0.00) | 0.00 (0.00 to 0.00) | 0.00 (0.00 to 0.00) |
| Marshall Islands | 4.16 (3.17 to 5.52) | 4.96 (3.75 to 6.63) | 5.17 (3.88 to 6.96) | 0.84 (-1.42 to 3.17) | 1.37 (-4.10 to 7.14) | 0.71 (-0.75 to 2.20) |
| Macedonia | 6.66 (4.97 to 8.90) | 5.12 (3.74 to 6.97) | 4.77 (3.56 to 6.39) | -1.28 (-3.26 to 0.75) | -0.42 (-1.35 to 0.52) | -1.08 (-1.72 to -0.42) |
| Mali | 1.94 (1.39 to 2.79) | 4.47 (3.32 to 6.17) | 3.87 (2.83 to 5.37) | 5.24 (-0.21 to 10.98) | -1.38 (-4.03 to 1.35) | 0.96 (-1.39 to 3.38) |
| Malta | 6.75 (4.94 to 9.32) | 5.56 (3.89 to 8.14) | 4.89 (3.56 to 6.76) | -1.19 (-1.76 to -0.61) | -0.23 (-6.17 to 6.09) | 0.05 (-1.51 to 1.64) |
| Myanmar | 3.96 (2.91 to 5.49) | 4.93 (3.70 to 6.62) | 7.24 (5.32 to 10.01) | 1.47 (0.59 to 2.36) | 2.42 (0.12 to 4.77) | 1.98 (1.31 to 2.66) |
| Montenegro | 5.03 (3.76 to 6.72) | 4.89 (3.57 to 6.71) | 5.07 (3.76 to 6.82) | 0.09 (-1.37 to 1.58) | 0.48 (-0.28 to 1.25) | 0.03 (-0.42 to 0.49) |
| Mongolia | 13.52 (9.99 to 18.30) | 5.52 (4.09 to 7.48) | 7.37 (5.43 to 9.98) | -4.60 (-12.34 to 3.83) | 2.14 (0.44 to 3.86) | -0.46 (-3.39 to 2.56) |
| Mozambique | 4.80 (4.22 to 5.45) | 5.30 (4.72 to 5.98) | 5.35 (4.77 to 6.05) | 0.77 (0.19 to 1.34) | 0.00 (-0.36 to 0.37) | 0.49 (0.24 to 0.75) |
| Mauritania | 6.74 (5.14 to 9.00) | 4.65 (3.65 to 6.03) | 9.33 (7.07 to 12.47) | -0.74 (-7.95 to 7.03) | 5.66 (3.24 to 8.14) | 2.37 (-0.16 to 4.96) |
| Mauritius | 5.42 (4.26 to 6.96) | 4.80 (3.82 to 6.06) | 5.28 (4.15 to 6.80) | -0.38 (-3.06 to 2.37) | 0.19 (-1.74 to 2.15) | 0.13 (-0.79 to 1.06) |
| Malawi | 3.66 (3.37 to 3.96) | 3.67 (3.38 to 3.98) | 4.03 (3.72 to 4.35) | 0.02 (-0.00 to 0.03) | 1.10 (-0.21 to 2.43) | 0.48 (0.08 to 0.89) |
| Malaysia | 2.18 (2.03 to 2.35) | 2.34 (2.17 to 2.52) | 3.01 (2.80 to 3.22) | 0.35 (-0.42 to 1.12) | 1.60 (-2.01 to 5.36) | 1.47 (0.52 to 2.43) |
| Namibia | 3.76 (3.49 to 4.08) | 3.78 (3.50 to 4.09) | 6.55 (6.02 to 7.16) | 0.71 (-2.74 to 4.29) | 5.21 (1.83 to 8.69) | 1.82 (0.18 to 3.50) |
| Niger | 2.88 (2.62 to 3.17) | 3.77 (3.43 to 4.14) | 3.99 (3.63 to 4.38) | 1.68 (0.08 to 3.31) | 0.82 (-0.60 to 2.26) | 1.62 (1.02 to 2.22) |
| Nigeria | 3.50 (3.26 to 3.77) | 3.52 (3.28 to 3.79) | 3.53 (3.28 to 3.79) | 0.03 (0.02 to 0.05) | 0.01 (-0.06 to 0.07) | 0.03 (0.01 to 0.05) |
| Nicaragua | 3.94 (2.89 to 5.44) | 3.95 (2.94 to 5.36) | 3.94 (2.89 to 5.50) | 0.02 (-0.00 to 0.03) | -0.02 (-0.04 to -0.01) | 0.00 (-0.01 to 0.01) |
| Netherlands | 6.18 (5.93 to 6.45) | 6.61 (6.32 to 6.91) | 6.31 (6.04 to 6.60) | 0.41 (0.19 to 0.63) | -0.26 (-0.76 to 0.25) | 0.06 (-0.12 to 0.24) |
| Norway | 4.38 (3.24 to 6.04) | 5.00 (3.64 to 7.01) | 4.81 (3.49 to 6.73) | 0.99 (0.34 to 1.65) | -0.09 (-1.05 to 0.88) | 0.60 (0.22 to 0.99) |
| Nepal | 3.54 (3.20 to 3.93) | 4.59 (4.15 to 5.08) | 5.29 (4.78 to 5.87) | 1.87 (1.00 to 2.75) | 1.22 (0.79 to 1.66) | 1.64 (1.35 to 1.93) |
| New Zealand | 9.42 (6.87 to 13.17) | 5.22 (3.79 to 7.30) | 4.39 (3.19 to 6.13) | -3.56 (-5.34 to -1.75) | -1.65 (-4.05 to 0.80) | -2.63 (-3.36 to -1.88) |
| Oman | 4.07 (2.91 to 5.78) | 7.82 (5.59 to 11.13) | 5.74 (4.01 to 8.39) | 4.48 (4.10 to 4.86) | -2.39 (-3.48 to -1.29) | 0.95 (-0.56 to 2.49) |
| Pakistan | 6.08 (4.35 to 8.59) | 5.63 (4.15 to 7.68) | 5.26 (3.78 to 7.36) | -0.59 (-1.34 to 0.17) | -0.34 (-2.91 to 2.31) | -0.74 (-1.41 to -0.07) |
| Panama | 3.87 (2.90 to 5.23) | 3.96 (2.94 to 5.39) | 3.87 (2.88 to 5.28) | 0.14 (0.01 to 0.28) | 0.24 (-1.33 to 1.83) | 0.25 (-0.14 to 0.65) |
| Peru | 3.95 (2.93 to 5.37) | 3.93 (2.86 to 5.50) | 3.93 (2.89 to 5.41) | 0.01 (-1.61 to 1.65) | 0.00 (-0.00 to 0.00) | -0.30 (-0.78 to 0.18) |
| Philippines | 4.11 (3.85 to 4.39) | 4.10 (3.85 to 4.39) | 4.11 (3.85 to 4.39) | -0.01 (-0.03 to 0.00) | 0.02 (0.01 to 0.04) | -0.00 (-0.01 to 0.01) |
| Papua New Guinea | 4.05 (2.94 to 5.69) | 4.93 (3.59 to 6.79) | 7.28 (5.23 to 10.29) | 0.98 (-1.36 to 3.36) | 3.54 (-3.22 to 10.76) | 1.52 (-0.40 to 3.47) |
| Poland | 5.25 (5.05 to 5.47) | 12.73 (12.23 to 13.23) | 8.03 (7.74 to 8.33) | 5.10 (-3.44 to 14.39) | -5.46 (-13.35 to 3.14) | 0.04 (-4.04 to 4.28) |
| Portugal | 3.93 (3.82 to 4.04) | 3.98 (3.87 to 4.09) | 6.16 (5.92 to 6.42) | 0.09 (0.05 to 0.13) | 2.72 (-0.97 to 6.55) | 0.97 (-0.15 to 2.10) |
| Paraguay | 5.59 (4.11 to 7.68) | 5.52 (4.07 to 7.51) | 6.12 (4.46 to 8.45) | -0.29 (-1.25 to 0.67) | 0.76 (0.42 to 1.10) | 0.30 (-0.09 to 0.70) |
| Palestine | 7.56 (5.39 to 10.80) | 7.50 (5.37 to 10.68) | 7.50 (5.31 to 10.78) | 0.01 (-0.38 to 0.41) | -0.20 (-1.48 to 1.09) | 0.14 (-0.23 to 0.51) |
| Qatar | 7.49 (5.09 to 11.15) | 7.50 (5.09 to 11.18) | 7.12 (4.76 to 10.89) | 0.07 (-0.30 to 0.44) | -0.58 (-1.57 to 0.43) | -0.05 (-0.38 to 0.28) |
| Romania | 3.55 (3.36 to 3.74) | 3.49 (3.31 to 3.68) | 3.53 (3.34 to 3.73) | -0.11 (-0.14 to -0.08) | 0.12 (-0.04 to 0.29) | -0.02 (-0.09 to 0.05) |
| Russia | 3.60 (2.62 to 4.98) | 5.63 (3.95 to 8.11) | 4.99 (3.62 to 6.90) | 2.78 (0.71 to 4.90) | -0.85 (-1.56 to -0.13) | 0.91 (-0.15 to 1.98) |
| Rwanda | 3.89 (2.76 to 5.66) | 3.97 (3.00 to 5.35) | 3.93 (2.86 to 5.55) | 0.14 (0.12 to 0.16) | -0.10 (-0.22 to 0.01) | 0.04 (-0.03 to 0.10) |
| Saudi Arabia | 7.33 (5.20 to 10.48) | 7.43 (5.27 to 10.61) | 7.64 (5.38 to 11.03) | 0.82 (-2.57 to 4.33) | 0.26 (0.05 to 0.47) | 0.58 (-0.38 to 1.55) |
| Sudan | 4.87 (3.71 to 6.53) | 4.12 (3.20 to 5.39) | 13.18 (10.06 to 17.68) | -0.83 (-2.71 to 1.08) | 10.96 (5.14 to 17.10) | 4.81 (1.82 to 7.88) |
| Senegal | 3.18 (2.39 to 4.29) | 4.32 (3.19 to 5.98) | 4.29 (3.23 to 5.83) | 2.21 (0.43 to 4.02) | -0.57 (-2.33 to 1.23) | 0.63 (-0.35 to 1.61) |
| Singapore | 3.76 (2.70 to 5.32) | 5.09 (3.68 to 7.08) | 4.27 (3.05 to 6.08) | 2.42 (0.25 to 4.64) | -1.35 (-1.71 to -0.99) | 0.18 (-0.94 to 1.32) |
| Solomon Islands | 4.99 (3.69 to 6.83) | 4.50 (3.35 to 6.13) | 9.21 (6.92 to 12.40) | -0.62 (-1.31 to 0.08) | 5.17 (0.80 to 9.73) | 1.60 (-0.15 to 3.39) |
| Sierra Leone | 3.53 (3.26 to 3.82) | 3.54 (3.28 to 3.84) | 3.53 (3.27 to 3.83) | 0.02 (-0.00 to 0.04) | -0.04 (-0.12 to 0.04) | 0.01 (-0.02 to 0.03) |
| El Salvador | 3.95 (2.89 to 5.45) | 3.94 (2.95 to 5.32) | 3.93 (2.85 to 5.51) | -0.02 (-0.03 to 0.00) | -0.03 (-0.07 to 0.01) | -0.02 (-0.04 to -0.01) |
| Serbia | 5.01 (3.73 to 6.70) | 4.89 (3.59 to 6.66) | 4.97 (3.71 to 6.67) | 0.12 (-1.32 to 1.58) | 0.16 (0.01 to 0.32) | -0.13 (-0.56 to 0.30) |
| South Sudan | 4.78 (3.55 to 6.61) | 4.11 (3.15 to 5.49) | 7.23 (5.43 to 9.89) | -0.73 (-2.58 to 1.16) | 3.95 (1.17 to 6.80) | 2.03 (0.67 to 3.41) |
| Sao Tome and Principe | 3.84 (3.55 to 4.17) | 3.88 (3.59 to 4.21) | 3.90 (3.60 to 4.23) | 0.06 (0.03 to 0.09) | 0.03 (-0.02 to 0.07) | 0.05 (0.04 to 0.07) |
| Suriname | 3.81 (2.83 to 5.22) | 4.01 (2.97 to 5.47) | 7.10 (5.27 to 9.72) | 0.26 (-0.61 to 1.13) | 4.52 (1.80 to 7.30) | 2.83 (1.63 to 4.04) |
| Slovakia | 2.31 (1.89 to 2.83) | 2.27 (1.85 to 2.78) | 3.06 (2.50 to 3.77) | 0.08 (-0.94 to 1.10) | 1.20 (-4.08 to 6.77) | 1.18 (-0.16 to 2.54) |
| Slovenia | 4.92 (3.61 to 6.70) | 4.87 (3.53 to 6.73) | 4.87 (3.58 to 6.62) | 1.23 (-5.30 to 8.20) | 0.02 (-0.05 to 0.10) | -0.50 (-2.48 to 1.52) |
| Sweden | 3.56 (3.32 to 3.81) | 3.71 (3.46 to 3.97) | 3.88 (3.63 to 4.15) | 0.49 (-0.78 to 1.78) | 0.10 (-0.96 to 1.18) | 0.54 (0.06 to 1.01) |
| Swaziland | 3.36 (3.08 to 3.67) | 3.59 (3.31 to 3.89) | 3.76 (3.47 to 4.08) | 0.48 (-0.67 to 1.65) | 0.38 (-0.07 to 0.82) | 0.28 (-0.07 to 0.62) |
| Seychelles | 6.81 (6.28 to 7.37) | 5.44 (5.01 to 5.90) | 11.59 (10.73 to 12.53) | -1.10 (-3.67 to 1.53) | 5.30 (1.34 to 9.41) | 2.70 (0.80 to 4.63) |
| Syria | 7.66 (5.42 to 11.00) | 7.56 (5.40 to 10.70) | 7.69 (5.44 to 11.10) | -0.02 (-0.42 to 0.38) | -0.02 (-1.11 to 1.09) | 0.18 (-0.13 to 0.50) |
| Chad | 3.72 (3.43 to 4.03) | 3.31 (3.05 to 3.60) | 3.30 (3.04 to 3.58) | -0.69 (-1.50 to 0.13) | -0.03 (-0.09 to 0.02) | -0.27 (-0.56 to 0.02) |
| Togo | 4.01 (3.13 to 5.26) | 4.05 (3.21 to 5.22) | 4.05 (3.18 to 5.25) | 0.06 (0.05 to 0.08) | -0.01 (-0.07 to 0.05) | 0.04 (0.01 to 0.06) |
| Thailand | 2.27 (1.69 to 3.09) | 5.11 (3.93 to 6.72) | 6.24 (4.67 to 8.41) | 5.55 (5.04 to 6.05) | 1.44 (0.87 to 2.03) | 3.60 (2.65 to 4.57) |
| Tajikistan | 2.40 (1.76 to 3.28) | 5.63 (4.00 to 7.91) | 3.31 (2.42 to 4.58) | 6.12 (2.95 to 9.38) | -4.73 (-7.37 to -2.00) | 1.60 (-0.99 to 4.25) |
| Turkmenistan | 1.76 (1.31 to 2.38) | 5.90 (4.26 to 8.23) | 3.16 (2.35 to 4.27) | 6.23 (-7.14 to 21.53) | -5.29 (-7.33 to -3.20) | -0.49 (-5.13 to 4.39) |
| Timor-Leste | 4.25 (3.98 to 4.56) | 4.24 (3.97 to 4.55) | 4.25 (3.98 to 4.55) | -0.01 (-0.03 to 0.00) | 0.02 (0.01 to 0.04) | -0.00 (-0.01 to 0.01) |
| Tonga | 4.11 (2.98 to 5.77) | 4.91 (3.59 to 6.85) | 5.20 (3.76 to 7.34) | 0.85 (-1.44 to 3.20) | 1.51 (-4.03 to 7.37) | 0.78 (-0.71 to 2.29) |
| Trinidad and Tobago | 3.28 (2.37 to 4.60) | 4.08 (3.00 to 5.61) | 3.44 (2.49 to 4.82) | 1.88 (0.14 to 3.65) | -1.01 (-2.05 to 0.04) | 0.23 (-0.65 to 1.12) |
| Tunisia | 9.13 (6.47 to 13.08) | 7.53 (5.42 to 10.65) | 5.50 (3.90 to 7.90) | -0.81 (-3.20 to 1.63) | -3.08 (-5.52 to -0.58) | -1.58 (-2.63 to -0.51) |
| Turkey | 5.46 (3.64 to 8.10) | 5.76 (3.84 to 8.55) | 5.79 (3.85 to 8.59) | 0.37 (0.14 to 0.59) | -0.01 (-0.19 to 0.17) | 0.24 (0.13 to 0.35) |
| Taiwan | 3.95 (3.01 to 5.24) | 5.37 (3.95 to 7.40) | 5.25 (3.92 to 7.08) | 1.98 (1.57 to 2.39) | -0.32 (-1.48 to 0.85) | 1.07 (0.44 to 1.71) |
| Tanzania | 3.80 (3.52 to 4.11) | 3.82 (3.53 to 4.13) | 4.47 (4.13 to 4.83) | 0.04 (0.02 to 0.05) | 0.95 (-0.43 to 2.36) | 0.34 (-0.07 to 0.76) |
| Uganda | 3.54 (3.28 to 3.81) | 3.56 (3.30 to 3.83) | 3.55 (3.30 to 3.82) | -1.25 (-7.66 to 5.60) | -0.03 (-0.08 to 0.02) | -0.96 (-2.80 to 0.92) |
| Ukraine | 3.27 (2.39 to 4.47) | 5.22 (3.73 to 7.37) | 4.66 (3.40 to 6.41) | 3.44 (1.54 to 5.38) | -0.73 (-1.32 to -0.15) | 1.68 (0.65 to 2.72) |
| Uruguay | 3.83 (2.73 to 5.44) | 5.30 (3.70 to 7.77) | 5.64 (3.96 to 8.14) | 2.02 (0.57 to 3.48) | 0.50 (0.30 to 0.71) | 1.16 (0.60 to 1.72) |
| United States | 4.68 (4.50 to 4.86) | 4.92 (4.75 to 5.10) | 4.84 (4.67 to 5.03) | 0.33 (0.16 to 0.49) | -0.12 (-0.25 to 0.01) | 0.11 (-0.01 to 0.23) |
| Uzbekistan | 4.35 (3.93 to 4.82) | 8.40 (7.52 to 9.42) | 8.55 (7.64 to 9.58) | 4.67 (3.25 to 6.10) | 0.26 (-1.23 to 1.78) | 2.05 (0.88 to 3.24) |
| Saint Vincent and the Grenadines | 3.93 (2.92 to 5.35) | 3.94 (2.93 to 5.36) | 5.39 (4.02 to 7.32) | 0.02 (0.01 to 0.03) | 3.20 (-1.54 to 8.16) | 2.01 (0.67 to 3.36) |
| Venezuela | 3.93 (2.84 to 5.56) | 3.94 (2.85 to 5.53) | 3.92 (2.82 to 5.58) | 0.01 (-0.02 to 0.04) | -0.04 (-0.06 to -0.01) | -0.01 (-0.02 to 0.01) |
| Vietnam | 3.36 (3.14 to 3.61) | 5.68 (5.26 to 6.12) | 8.57 (7.88 to 9.33) | 2.65 (-2.36 to 7.91) | 3.13 (2.74 to 3.52) | 3.86 (2.31 to 5.44) |
| Vanuatu | 4.51 (3.38 to 6.08) | 4.52 (3.39 to 6.10) | 4.53 (3.42 to 6.07) | 0.02 (0.01 to 0.03) | 0.02 (-0.02 to 0.05) | 0.01 (0.00 to 0.02) |
| Samoa | 4.09 (3.07 to 5.51) | 4.66 (3.49 to 6.30) | 6.60 (4.93 to 8.94) | 0.48 (-1.49 to 2.49) | 2.91 (2.22 to 3.62) | 1.92 (1.05 to 2.80) |
| Yemen | 7.74 (5.11 to 11.90) | 7.26 (5.03 to 10.66) | 5.17 (3.50 to 7.78) | -0.84 (-4.39 to 2.84) | -4.41 (-10.48 to 2.08) | -1.55 (-3.60 to 0.55) |
| South Africa | 4.12 (3.83 to 4.45) | 4.50 (4.17 to 4.87) | 3.38 (3.12 to 3.67) | 0.28 (-1.69 to 2.29) | -2.21 (-2.72 to -1.70) | -0.59 (-1.29 to 0.11) |
| Zambia | 3.76 (3.52 to 4.02) | 3.78 (3.54 to 4.03) | 3.77 (3.54 to 4.02) | 0.03 (-0.01 to 0.07) | -0.04 (-0.11 to 0.04) | 0.01 (-0.01 to 0.04) |
| Zimbabwe | 3.50 (3.21 to 3.82) | 3.81 (3.53 to 4.12) | 3.55 (3.26 to 3.86) | 0.47 (-0.24 to 1.18) | -0.67 (-1.22 to -0.12) | 0.14 (-0.21 to 0.49) |

CI=confidence interval; EAPC=estimated annual percentage change; UI=uncertainty interval.

The standardized serving size used for this analysis was a cup (8oz). Total green or black tea intake, including caffeinated, decaffeinated, sweetened or unsweetened tea. This definition excludes herbal tea.

*In previous Global Dietary Database reports, the region central or eastern Europe and central Asia was referred to as the former Soviet Union, and southeast and east Asia was referred to as Asia.

**Supplementary Table 2. National mean tea intake (cup (8 oz) per week) in adults aged ≥20 years by sex, age, parental education, and area of residence in the 25 most populous countries in 2018.**

| **Country** | **Mean (95% UI)** | | | | | | | | | | |
| --- | --- | --- | --- | --- | --- | --- | --- | --- | --- | --- | --- |
|  | **Overall** | **Sex** | | **Age** | | | **Education** | | | **Area of residence** | |
|  |  | **Female** | **Male** | **20-39 years** | **40-59 years** | **≥60 years** | **0-6 years** | **>6-12 years** | **>12 years** | **Rural** | **Urban** |
| India | 5.47 (4.93 to 6.08) | 5.57 (5.02 to 6.19) | 5.38 (4.82 to 6.02) | 5.12 (4.74 to 5.52) | 5.81 (5.22 to 6.46) | 6.00 (4.99 to 7.21) | 5.37 (4.83 to 5.96) | 5.61 (5.04 to 6.26) | 5.64 (5.06 to 6.30) | 5.47 (4.92 to 6.08) | 5.49 (4.93 to 6.11) |
| China | 8.04 (6.11 to 10.55) | 8.19 (5.85 to 11.54) | 7.81 (5.63 to 10.79) | 8.05 (6.13 to 10.55) | 8.09 (6.14 to 10.61) | 7.91 (6.00 to 10.42) | 8.03 (5.51 to 11.69) | 7.94 (5.47 to 11.48) | 7.92 (5.48 to 11.51) | 7.94 (5.72 to 11.06) | 8.03 (5.79 to 11.18) |
| Nigeria | 3.53 (3.28 to 3.79) | 3.42 (3.18 to 3.69) | 3.63 (3.35 to 3.93) | 3.46 (3.24 to 3.70) | 3.63 (3.39 to 3.90) | 3.60 (3.21 to 4.03) | 3.49 (3.25 to 3.75) | 3.60 (3.35 to 3.88) | 3.72 (3.44 to 4.01) | 3.45 (3.21 to 3.71) | 3.61 (3.36 to 3.89) |
| Pakistan | 5.26 (3.78 to 7.36) | 5.25 (3.52 to 8.02) | 5.19 (3.52 to 7.79) | 4.96 (3.58 to 6.86) | 5.64 (4.06 to 7.93) | 5.84 (4.09 to 8.45) | 5.16 (3.45 to 7.71) | 5.38 (3.61 to 8.08) | 5.40 (3.63 to 8.13) | 5.22 (3.55 to 7.81) | 5.24 (3.56 to 7.78) |
| Indonesia | 4.21 (3.95 to 4.51) | 4.23 (3.93 to 4.56) | 4.20 (3.90 to 4.52) | 4.22 (3.96 to 4.51) | 4.23 (3.97 to 4.53) | 4.14 (3.88 to 4.43) | 4.24 (3.97 to 4.53) | 4.18 (3.92 to 4.48) | 4.17 (3.91 to 4.47) | 4.19 (3.93 to 4.48) | 4.23 (3.96 to 4.52) |
| United States | 4.84 (4.67 to 5.03) | 4.88 (4.70 to 5.07) | 4.80 (4.62 to 4.99) | 4.46 (4.30 to 4.62) | 5.01 (4.84 to 5.20) | 5.14 (4.95 to 5.35) | 4.45 (4.28 to 4.63) | 4.86 (4.68 to 5.05) | 4.86 (4.68 to 5.05) | 4.77 (4.66 to 4.88) | 4.86 (4.66 to 5.07) |
| Brazil | 3.66 (3.40 to 3.94) | 3.68 (3.36 to 4.03) | 3.64 (3.33 to 3.96) | 3.67 (3.43 to 3.94) | 3.75 (3.50 to 4.02) | 3.46 (3.15 to 3.81) | 3.66 (3.39 to 3.95) | 3.66 (3.40 to 3.96) | 3.64 (3.36 to 3.95) | 3.66 (3.38 to 3.97) | 3.66 (3.40 to 3.94) |
| Bangladesh | 4.45 (4.08 to 4.86) | 4.17 (3.81 to 4.57) | 4.71 (4.23 to 5.28) | 4.16 (3.88 to 4.48) | 4.74 (4.37 to 5.14) | 4.90 (4.18 to 5.74) | 4.36 (3.99 to 4.78) | 4.56 (4.16 to 5.00) | 4.58 (4.18 to 5.03) | 4.44 (4.07 to 4.85) | 4.46 (4.06 to 4.91) |
| Ethiopia | 4.19 (3.95 to 4.46) | 4.68 (4.39 to 4.99) | 3.70 (3.44 to 3.98) | 4.11 (3.91 to 4.34) | 4.34 (4.09 to 4.61) | 4.32 (3.87 to 4.80) | 4.15 (3.91 to 4.41) | 4.28 (4.03 to 4.55) | 4.42 (4.14 to 4.72) | 4.16 (3.92 to 4.42) | 4.35 (4.09 to 4.63) |
| Democratic Republic of the Congo | 4.02 (3.10 to 5.32) | 4.02 (2.84 to 5.88) | 3.96 (2.82 to 5.69) | 3.95 (3.05 to 5.20) | 4.16 (3.20 to 5.51) | 4.11 (3.11 to 5.51) | 3.96 (2.83 to 5.59) | 4.08 (2.92 to 5.80) | 4.23 (3.03 to 5.97) | 3.91 (2.77 to 5.69) | 4.09 (2.91 to 5.93) |
| Mexico | 3.53 (3.18 to 3.92) | 3.55 (3.14 to 4.02) | 3.50 (3.10 to 3.95) | 3.54 (3.22 to 3.90) | 3.62 (3.26 to 4.01) | 3.33 (2.91 to 3.82) | 3.53 (3.17 to 3.94) | 3.54 (3.18 to 3.93) | 3.52 (3.16 to 3.92) | 3.53 (3.18 to 3.93) | 3.53 (3.18 to 3.93) |
| Philippines | 4.11 (3.85 to 4.39) | 4.28 (4.01 to 4.58) | 3.93 (3.65 to 4.24) | 4.11 (3.85 to 4.39) | 4.13 (3.87 to 4.41) | 4.06 (3.80 to 4.35) | 4.15 (3.89 to 4.44) | 4.10 (3.84 to 4.39) | 4.09 (3.83 to 4.37) | 4.09 (3.83 to 4.38) | 4.13 (3.87 to 4.42) |
| Egypt | 9.66 (8.77 to 10.63) | 9.71 (8.86 to 10.66) | 9.61 (8.64 to 10.66) | 9.04 (8.24 to 9.88) | 10.29 (9.33 to 11.35) | 10.74 (9.63 to 12.01) | 9.59 (8.71 to 10.57) | 9.60 (8.68 to 10.58) | 10.00 (9.04 to 11.06) | 9.92 (8.98 to 10.93) | 9.33 (8.46 to 10.28) |
| Russia | 4.99 (3.62 to 6.90) | 5.03 (3.39 to 7.55) | 4.86 (3.31 to 7.20) | 5.09 (3.72 to 6.98) | 5.03 (3.64 to 6.95) | 4.80 (3.46 to 6.73) | 5.55 (3.67 to 8.46) | 4.96 (3.28 to 7.53) | 4.82 (3.19 to 7.32) | 5.08 (3.52 to 7.40) | 4.93 (3.40 to 7.18) |
| Tanzania | 4.47 (4.13 to 4.83) | 4.57 (4.22 to 4.95) | 4.36 (3.97 to 4.78) | 4.39 (4.08 to 4.71) | 4.61 (4.27 to 4.99) | 4.58 (4.07 to 5.17) | 4.41 (4.08 to 4.77) | 4.55 (4.20 to 4.92) | 4.70 (4.32 to 5.11) | 4.40 (4.07 to 4.77) | 4.60 (4.25 to 4.98) |
| Vietnam | 8.57 (7.88 to 9.33) | 8.59 (7.84 to 9.42) | 8.56 (7.81 to 9.39) | 8.59 (7.90 to 9.34) | 8.61 (7.91 to 9.37) | 8.43 (7.75 to 9.18) | 8.63 (7.93 to 9.40) | 8.53 (7.84 to 9.28) | 8.51 (7.81 to 9.25) | 8.55 (7.85 to 9.30) | 8.63 (7.94 to 9.39) |
| Turkey | 5.79 (3.85 to 8.59) | 5.68 (3.69 to 8.59) | 5.89 (3.88 to 8.81) | 5.36 (3.58 to 7.92) | 6.08 (4.04 to 9.04) | 6.36 (4.20 to 9.52) | 5.74 (3.82 to 8.56) | 5.73 (3.82 to 8.53) | 6.00 (3.98 to 8.88) | 6.06 (4.01 to 8.98) | 5.69 (3.79 to 8.46) |
| Kenya | 13.09 (11.66 to 14.72) | 13.24 (11.73 to 15.00) | 12.94 (11.42 to 14.63) | 12.86 (11.51 to 14.39) | 13.51 (12.03 to 15.21) | 13.39 (11.53 to 15.56) | 12.91 (11.50 to 14.52) | 13.31 (11.85 to 14.97) | 13.76 (12.24 to 15.52) | 12.94 (11.53 to 14.55) | 13.53 (12.04 to 15.23) |
| Iran | 17.46 (16.55 to 18.50) | 16.19 (15.16 to 17.34) | 18.73 (17.60 to 19.99) | 16.34 (15.46 to 17.33) | 18.45 (17.58 to 19.44) | 19.33 (18.17 to 20.61) | 17.27 (16.26 to 18.39) | 17.27 (16.31 to 18.34) | 17.99 (16.96 to 19.17) | 18.25 (17.10 to 19.57) | 17.18 (16.28 to 18.20) |
| Uganda | 3.55 (3.30 to 3.82) | 3.48 (3.23 to 3.76) | 3.61 (3.32 to 3.93) | 3.49 (3.26 to 3.73) | 3.67 (3.42 to 3.96) | 3.64 (3.23 to 4.08) | 3.51 (3.26 to 3.78) | 3.62 (3.36 to 3.90) | 3.74 (3.46 to 4.05) | 3.51 (3.27 to 3.78) | 3.67 (3.41 to 3.95) |
| Japan | 14.95 (12.74 to 17.54) | 16.19 (13.51 to 19.34) | 13.61 (11.34 to 16.21) | 15.01 (12.80 to 17.63) | 15.06 (12.83 to 17.66) | 14.82 (12.64 to 17.40) | 15.13 (12.87 to 17.80) | 14.95 (12.72 to 17.56) | 14.92 (12.72 to 17.50) | 14.83 (12.63 to 17.41) | 14.96 (12.75 to 17.55) |
| South Africa | 3.38 (3.12 to 3.67) | 3.38 (3.09 to 3.69) | 3.38 (3.10 to 3.69) | 3.32 (3.09 to 3.56) | 3.47 (3.22 to 3.76) | 3.43 (3.04 to 3.88) | 3.30 (3.05 to 3.58) | 3.40 (3.14 to 3.69) | 3.52 (3.24 to 3.82) | 3.29 (3.04 to 3.56) | 3.43 (3.17 to 3.73) |
| Sudan | 13.18 (10.06 to 17.68) | 13.20 (9.20 to 19.51) | 12.95 (9.08 to 18.94) | 12.95 (9.91 to 17.30) | 13.59 (10.37 to 18.26) | 13.48 (10.10 to 18.36) | 12.97 (9.18 to 18.62) | 13.37 (9.45 to 19.21) | 13.83 (9.70 to 19.96) | 12.90 (9.13 to 18.63) | 13.49 (9.54 to 19.36) |
| Afghanistan | 13.74 (9.40 to 20.41) | 13.58 (8.40 to 22.70) | 13.48 (8.48 to 22.14) | 13.05 (8.96 to 19.32) | 14.89 (10.17 to 22.06) | 15.42 (10.27 to 23.57) | 13.60 (8.97 to 20.88) | 14.23 (9.38 to 21.95) | 14.31 (9.44 to 22.10) | 13.62 (8.73 to 21.53) | 13.64 (8.74 to 21.61) |
| Myanmar | 7.24 (5.32 to 10.01) | 7.33 (4.96 to 11.13) | 6.99 (4.80 to 10.47) | 7.24 (5.35 to 10.05) | 7.27 (5.34 to 10.02) | 7.14 (5.19 to 9.85) | 7.21 (4.92 to 10.76) | 7.13 (4.87 to 10.59) | 7.12 (4.86 to 10.60) | 7.17 (4.94 to 10.57) | 7.24 (5.02 to 10.69) |

UI=uncertainty interval.

The standardized serving size used for this analysis was a cup (8oz). Total green or black tea intake, including caffeinated, decaffeinated, sweetened or unsweetened tea. This definition excludes herbal tea.

**Supplementary Table 3. Mean tea intake (cup (8 oz) per week) in adults aged ≥20 years by age, education, and area of residence, presented by global and regional estimates stratified by sex across 185 countries in 2018.**

|  | **Mean (95% UI)** | | | | | | | | | | | | | | | | | |
| --- | --- | --- | --- | --- | --- | --- | --- | --- | --- | --- | --- | --- | --- | --- | --- | --- | --- | --- |
|  | **Worldwide** | | | **Central and eastern Europe and central Asia*** | | | **High income countries** | | **Latin America and the Caribbean** | | **Middle East and north Africa** | | **South Asia*** | | **Southeast and east Asia** | | **Sub-Saharan Africa** | |
|  | Female | Male | Female | | Male | Female | | Male | Female | Male | Female | Male | Female | Male | Female | Male | Female | Male |
| **Overall** | 6.24 (5.64 to 7.03) | 6.10 (5.52 to 6.84) | 5.47 (4.59 to 6.68) | | 5.42 (4.60 to 6.50) | 5.01 (4.61 to 5.52) | | 4.74 (4.37 to 5.20) | 3.90 (3.54 to 4.33) | 3.80 (3.45 to 4.22) | 8.73 (7.68 to 10.10) | 9.16 (8.11 to 10.59) | 5.51 (4.96 to 6.16) | 5.41 (4.85 to 6.06) | 7.81 (6.24 to 10.05) | 7.43 (5.93 to 9.44) | 4.99 (4.60 to 5.46) | 4.89 (4.50 to 5.35) |
| **Age (years)** |  |  |  | |  |  | |  |  |  |  |  |  |  |  |  |  |  |
| 20-39 | 5.93 (5.42 to 6.62) | 5.81 (5.31 to 6.44) | 5.70 (4.84 to 6.87) | | 5.63 (4.81 to 6.72) | 4.60 (4.25 to 5.06) | | 4.35 (4.03 to 4.75) | 3.90 (3.58 to 4.31) | 3.82 (3.50 to 4.21) | 8.21 (7.24 to 9.47) | 8.47 (7.51 to 9.80) | 5.19 (4.77 to 5.67) | 5.09 (4.67 to 5.58) | 7.53 (6.03 to 9.67) | 7.18 (5.74 to 9.12) | 4.91 (4.56 to 5.34) | 4.81 (4.46 to 5.22) |
| 40-59 | 6.48 (5.81 to 7.39) | 6.32 (5.67 to 7.16) | 5.47 (4.57 to 6.70) | | 5.45 (4.61 to 6.56) | 5.17 (4.75 to 5.71) | | 4.87 (4.49 to 5.34) | 3.98 (3.63 to 4.41) | 3.90 (3.54 to 4.31) | 9.29 (8.16 to 10.76) | 9.71 (8.57 to 11.23) | 5.84 (5.24 to 6.51) | 5.72 (5.12 to 6.38) | 7.85 (6.20 to 10.20) | 7.45 (5.90 to 9.54) | 5.15 (4.73 to 5.65) | 5.04 (4.64 to 5.51) |
| ≥60 | 6.56 (5.90 to 7.42) | 6.36 (5.72 to 7.19) | 5.05 (4.16 to 6.31) | | 5.13 (4.35 to 6.19) | 5.31 (4.87 to 5.85) | | 5.00 (4.59 to 5.49) | 3.71 (3.28 to 4.20) | 3.60 (3.18 to 4.10) | 9.59 (8.37 to 11.14) | 10.46 (9.23 to 12.05) | 6.02 (5.02 to 7.25) | 5.87 (4.87 to 7.06) | 8.30 (6.73 to 10.55) | 7.79 (6.29 to 9.82) | 5.06 (4.46 to 5.74) | 4.94 (4.35 to 5.63) |
| **Education (years)** |  |  |  | |  |  | |  |  |  |  |  |  |  |  |  |  |  |
| 0-6 | 6.08 (5.35 to 7.16) | 6.00 (5.28 to 7.00) | 5.94 (4.77 to 7.76) | | 5.78 (4.71 to 7.37) | 4.71 (3.99 to 5.75) | | 4.41 (3.77 to 5.34) | 3.85 (3.48 to 4.33) | 3.78 (3.40 to 4.23) | 8.62 (7.47 to 10.17) | 9.03 (7.88 to 10.62) | 5.49 (4.88 to 6.24) | 5.39 (4.77 to 6.13) | 7.29 (5.25 to 10.65) | 7.01 (5.07 to 10.06) | 4.91 (4.48 to 5.46) | 4.82 (4.39 to 5.35) |
| >6-12 | 6.30 (5.47 to 7.65) | 6.13 (5.31 to 7.38) | 5.50 (4.62 to 6.75) | | 5.53 (4.73 to 6.65) | 5.04 (4.43 to 5.92) | | 4.73 (4.16 to 5.51) | 3.90 (3.55 to 4.33) | 3.80 (3.45 to 4.22) | 8.26 (7.14 to 9.73) | 8.67 (7.52 to 10.22) | 5.56 (5.01 to 6.19) | 5.44 (4.88 to 6.08) | 7.85 (5.70 to 11.40) | 7.45 (5.43 to 10.69) | 5.24 (4.81 to 5.78) | 5.12 (4.70 to 5.65) |
| >12 | 6.32 (5.59 to 7.42) | 6.14 (5.44 to 7.20) | 5.31 (4.23 to 6.97) | | 5.22 (4.23 to 6.73) | 5.03 (4.72 to 5.45) | | 4.81 (4.52 to 5.18) | 3.97 (3.49 to 4.61) | 3.85 (3.38 to 4.47) | 9.66 (8.62 to 11.03) | 10.14 (9.03 to 11.63) | 5.40 (4.69 to 6.38) | 5.38 (4.67 to 6.34) | 8.21 (6.22 to 11.42) | 7.68 (5.82 to 10.65) | 4.80 (4.37 to 5.31) | 4.69 (4.28 to 5.17) |
| **Area of residence** |  |  |  | |  |  | |  |  |  |  |  |  |  |  |  |  |  |
| Rural | 6.18 (5.46 to 7.19) | 6.05 (5.35 to 7.02) | 5.69 (4.85 to 6.85) | | 5.62 (4.85 to 6.66) | 4.84 (4.44 to 5.37) | | 4.59 (4.22 to 5.06) | 3.93 (3.49 to 4.48) | 3.84 (3.40 to 4.38) | 9.04 (7.94 to 10.49) | 9.46 (8.35 to 10.91) | 5.52 (4.94 to 6.21) | 5.41 (4.83 to 6.10) | 7.36 (5.46 to 10.26) | 7.07 (5.27 to 9.76) | 5.10 (4.67 to 5.66) | 4.96 (4.53 to 5.50) |
| Urban | 6.26 (5.57 to 7.27) | 6.12 (5.45 to 7.06) | 5.34 (4.30 to 6.85) | | 5.29 (4.34 to 6.65) | 5.05 (4.62 to 5.61) | | 4.78 (4.38 to 5.28) | 3.89 (3.54 to 4.31) | 3.79 (3.45 to 4.21) | 8.57 (7.48 to 10.03) | 9.02 (7.92 to 10.54) | 5.50 (4.90 to 6.21) | 5.40 (4.80 to 6.11) | 8.07 (6.21 to 10.94) | 7.61 (5.87 to 10.22) | 4.80 (4.39 to 5.33) | 4.76 (4.35 to 5.27) |

UI=uncertainty interval.

The standardized serving size used for this analysis was a cup (8oz). Total green or black tea intake, including caffeinated, decaffeinated, sweetened or unsweetened tea. This definition excludes herbal tea.

*In previous Global Dietary Database reports, the region central or eastern Europe and central Asia was referred to as the former Soviet Union, and southeast and east Asia was referred to as Asia.

**Supplementary Table 4. National mean tea intake (cup (8 oz) per week) in adults aged ≥20 years by age, education, and area of residence, presented by global and regional estimates stratified by sex across in the 25 most populous countries in 2018.**

|  | **Mean (95% UI)** | | | | | | | | | | | | | | | | | |
| --- | --- | --- | --- | --- | --- | --- | --- | --- | --- | --- | --- | --- | --- | --- | --- | --- | --- | --- |
|  | **Worldwide** | | | **Central and eastern Europe and central Asia*** | | | **High income countries** | | **Latin America and the Caribbean** | | **Middle East and north Africa** | | **South Asia*** | | **Southeast and east Asia** | | **Sub-Saharan Africa** | |
|  | Low | High | Low | | High | Low | | High | Low | High | Low | High | Low | High | Low | High | Low | High |
| **Overall** | 6.05 (5.44 to 6.87) | 6.25 (5.65 to 7.06) | 5.88 (4.88 to 7.25) | | 5.28 (4.36 to 6.56) | 4.58 (3.99 to 5.37) | | 4.93 (4.65 to 5.26) | 3.82 (3.47 to 4.24) | 3.91 (3.47 to 4.47) | 8.82 (7.76 to 10.29) | 9.90 (8.88 to 11.23) | 5.44 (4.87 to 6.11) | 5.40 (4.75 to 6.22) | 7.22 (5.55 to 9.60) | 8.01 (6.42 to 10.30) | 4.87 (4.49 to 5.33) | 4.75 (4.36 to 5.18) |
| **Sex** |  |  |  | |  |  | |  |  |  |  |  |  |  |  |  |  |  |
| Female | 6.08 (5.35 to 7.16) | 6.32 (5.59 to 7.42) | 5.94 (4.77 to 7.76) | | 5.31 (4.23 to 6.97) | 4.71 (3.99 to 5.75) | | 5.03 (4.72 to 5.45) | 3.85 (3.48 to 4.33) | 3.97 (3.49 to 4.61) | 8.62 (7.47 to 10.17) | 9.66 (8.62 to 11.03) | 5.49 (4.88 to 6.24) | 5.40 (4.69 to 6.38) | 7.29 (5.25 to 10.65) | 8.21 (6.22 to 11.42) | 4.91 (4.48 to 5.46) | 4.80 (4.37 to 5.31) |
| Male | 6.00 (5.28 to 7.00) | 6.14 (5.44 to 7.20) | 5.78 (4.71 to 7.37) | | 5.22 (4.23 to 6.73) | 4.41 (3.77 to 5.34) | | 4.81 (4.52 to 5.18) | 3.78 (3.40 to 4.23) | 3.85 (3.38 to 4.47) | 9.03 (7.88 to 10.62) | 10.14 (9.03 to 11.63) | 5.39 (4.77 to 6.13) | 5.38 (4.67 to 6.34) | 7.01 (5.07 to 10.06) | 7.68 (5.82 to 10.65) | 4.82 (4.39 to 5.35) | 4.69 (4.28 to 5.17) |
| **Age (years)** |  |  |  | |  |  | |  |  |  |  |  |  |  |  |  |  |  |
| 20-39 | 5.74 (5.24 to 6.39) | 6.01 (5.44 to 6.76) | 6.15 (5.16 to 7.49) | | 5.50 (4.61 to 6.71) | 4.20 (3.67 to 4.91) | | 4.50 (4.26 to 4.80) | 3.83 (3.51 to 4.24) | 3.92 (3.51 to 4.45) | 8.19 (7.22 to 9.57) | 9.25 (8.31 to 10.49) | 5.13 (4.69 to 5.65) | 5.12 (4.58 to 5.83) | 7.05 (5.50 to 9.27) | 7.61 (6.03 to 9.88) | 4.78 (4.44 to 5.20) | 4.68 (4.33 to 5.08) |
| 40-59 | 6.31 (5.62 to 7.21) | 6.44 (5.79 to 7.34) | 5.91 (4.89 to 7.27) | | 5.31 (4.37 to 6.59) | 4.72 (4.10 to 5.53) | | 5.07 (4.79 to 5.42) | 3.90 (3.56 to 4.33) | 4.01 (3.56 to 4.56) | 9.42 (8.26 to 10.97) | 10.49 (9.39 to 11.91) | 5.75 (5.15 to 6.42) | 5.69 (5.03 to 6.49) | 7.30 (5.54 to 9.74) | 7.99 (6.35 to 10.38) | 5.03 (4.62 to 5.51) | 4.88 (4.48 to 5.32) |
| ≥60 | 6.36 (5.60 to 7.38) | 6.50 (5.92 to 7.27) | 5.46 (4.46 to 6.87) | | 4.93 (3.99 to 6.28) | 4.84 (4.21 to 5.68) | | 5.22 (4.92 to 5.60) | 3.62 (3.21 to 4.10) | 3.73 (3.23 to 4.35) | 9.93 (8.69 to 11.56) | 11.11 (9.96 to 12.56) | 5.92 (4.92 to 7.11) | 5.79 (4.78 to 7.03) | 7.39 (5.64 to 9.97) | 8.79 (7.25 to 10.95) | 4.95 (4.37 to 5.64) | 4.79 (4.22 to 5.44) |
| **Area of residence** |  |  |  | |  |  | |  |  |  |  |  |  |  |  |  |  |  |
| Rural | 6.01 (5.33 to 6.97) | 6.20 (5.30 to 7.61) | 6.06 (5.12 to 7.37) | | 5.50 (4.61 to 6.76) | 4.45 (3.85 to 5.28) | | 4.80 (4.53 to 5.15) | 3.87 (3.43 to 4.43) | 3.93 (3.40 to 4.61) | 9.00 (7.86 to 10.56) | 10.45 (9.38 to 11.78) | 5.46 (4.85 to 6.18) | 5.37 (4.68 to 6.29) | 7.05 (5.15 to 10.09) | 7.21 (5.07 to 10.69) | 4.94 (4.51 to 5.47) | 4.85 (4.43 to 5.35) |
| Urban | 6.07 (5.31 to 7.22) | 6.24 (5.62 to 7.16) | 5.75 (4.57 to 7.53) | | 5.15 (4.05 to 6.78) | 4.61 (3.95 to 5.51) | | 4.96 (4.66 to 5.33) | 3.80 (3.46 to 4.23) | 3.91 (3.47 to 4.48) | 8.72 (7.59 to 10.31) | 9.68 (8.62 to 11.11) | 5.40 (4.79 to 6.14) | 5.44 (4.70 to 6.46) | 7.25 (5.19 to 10.53) | 8.41 (6.60 to 11.20) | 4.75 (4.33 to 5.27) | 4.61 (4.20 to 5.09) |

UI=uncertainty interval.

The standardized serving size used for this analysis was a cup (8oz). Total green or black tea intake, including caffeinated, decaffeinated, sweetened or unsweetened tea. This definition excludes herbal tea.

*In previous Global Dietary Database reports, the region central or eastern Europe and central Asia was referred to as the former Soviet Union, and southeast and east Asia was referred to as Asia.

**Supplementary Table 5. Mean tea intake (cup (8 oz) per week) in adults aged ≥20 years by sex, age, and area of residence, presented by global and regional estimates stratified by education level across 185 countries in 2018.**

|  | **Mean (95% UI)** | | | | | | | | | | | | | | | | | |
| --- | --- | --- | --- | --- | --- | --- | --- | --- | --- | --- | --- | --- | --- | --- | --- | --- | --- | --- |
|  | **Worldwide** | | | **Central and eastern Europe and central Asia*** | | | **High income countries** | | **Latin America and the Caribbean** | | **Middle East and north Africa** | | **South Asia*** | | **Southeast and east Asia** | | **Sub-Saharan Africa** | |
|  | Rural | Urban | Rural | | Urban | Rural | | Urban | Rural | Urban | Rural | Urban | Rural | Urban | Rural | Urban | Rural | Urban |
| **Overall** | 6.13 (5.52 to 6.91) | 6.20 (5.63 to 6.96) | 5.65 (4.92 to 6.60) | | 5.33 (4.43 to 6.52) | 4.73 (4.37 to 5.15) | | 4.92 (4.55 to 5.38) | 3.89 (3.47 to 4.40) | 3.84 (3.52 to 4.23) | 9.25 (8.20 to 10.61) | 8.80 (7.76 to 10.22) | 5.47 (4.92 to 6.10) | 5.45 (4.89 to 6.10) | 7.26 (5.72 to 9.40) | 7.89 (6.38 to 9.98) | 5.04 (4.65 to 5.51) | 4.79 (4.41 to 5.24) |
| **Sex** |  |  |  | |  |  | |  |  |  |  |  |  |  |  |  |  |  |
| Female | 6.18 (5.46 to 7.19) | 6.26 (5.57 to 7.27) | 5.69 (4.85 to 6.85) | | 5.34 (4.30 to 6.85) | 4.84 (4.44 to 5.37) | | 5.05 (4.62 to 5.61) | 3.93 (3.49 to 4.48) | 3.89 (3.54 to 4.31) | 9.04 (7.94 to 10.49) | 8.57 (7.48 to 10.03) | 5.52 (4.94 to 6.21) | 5.50 (4.90 to 6.21) | 7.36 (5.46 to 10.26) | 8.07 (6.21 to 10.94) | 5.10 (4.67 to 5.66) | 4.80 (4.39 to 5.33) |
| Male | 6.05 (5.35 to 7.02) | 6.12 (5.45 to 7.06) | 5.62 (4.85 to 6.66) | | 5.29 (4.34 to 6.65) | 4.59 (4.22 to 5.06) | | 4.78 (4.38 to 5.28) | 3.84 (3.40 to 4.38) | 3.79 (3.45 to 4.21) | 9.46 (8.35 to 10.91) | 9.02 (7.92 to 10.54) | 5.41 (4.83 to 6.10) | 5.40 (4.80 to 6.11) | 7.07 (5.27 to 9.76) | 7.61 (5.87 to 10.22) | 4.96 (4.53 to 5.50) | 4.76 (4.35 to 5.27) |
| **Age (years)** |  |  |  | |  |  | |  |  |  |  |  |  |  |  |  |  |  |
| 20-39 | 5.83 (5.33 to 6.47) | 5.91 (5.40 to 6.59) | 5.91 (5.20 to 6.81) | | 5.53 (4.66 to 6.69) | 4.34 (4.03 to 4.71) | | 4.51 (4.18 to 4.91) | 3.90 (3.51 to 4.40) | 3.85 (3.56 to 4.21) | 8.60 (7.66 to 9.85) | 8.22 (7.26 to 9.55) | 5.15 (4.74 to 5.62) | 5.12 (4.70 to 5.60) | 7.12 (5.66 to 9.12) | 7.54 (6.08 to 9.61) | 4.95 (4.60 to 5.37) | 4.72 (4.38 to 5.12) |
| 40-59 | 6.38 (5.69 to 7.27) | 6.42 (5.78 to 7.27) | 5.68 (4.93 to 6.65) | | 5.35 (4.43 to 6.56) | 4.86 (4.50 to 5.30) | | 5.06 (4.68 to 5.54) | 3.97 (3.56 to 4.47) | 3.93 (3.61 to 4.32) | 9.88 (8.75 to 11.32) | 9.34 (8.21 to 10.85) | 5.78 (5.20 to 6.43) | 5.77 (5.17 to 6.44) | 7.34 (5.74 to 9.55) | 7.88 (6.33 to 10.04) | 5.21 (4.79 to 5.69) | 4.92 (4.54 to 5.38) |
| ≥60 | 6.39 (5.65 to 7.34) | 6.53 (5.92 to 7.31) | 5.25 (4.50 to 6.24) | | 4.99 (4.10 to 6.21) | 4.99 (4.61 to 5.46) | | 5.21 (4.80 to 5.71) | 3.68 (3.21 to 4.24) | 3.65 (3.25 to 4.12) | 10.32 (9.07 to 11.92) | 9.82 (8.64 to 11.35) | 5.94 (4.95 to 7.14) | 5.95 (4.95 to 7.17) | 7.40 (5.77 to 9.67) | 8.54 (7.03 to 10.55) | 5.10 (4.50 to 5.80) | 4.85 (4.28 to 5.51) |
| **Education (years)** |  |  |  | |  |  | |  |  |  |  |  |  |  |  |  |  |  |
| 0-6 | 6.01 (5.33 to 6.97) | 6.07 (5.31 to 7.22) | 6.06 (5.12 to 7.37) | | 5.75 (4.57 to 7.53) | 4.45 (3.85 to 5.28) | | 4.61 (3.95 to 5.51) | 3.87 (3.43 to 4.43) | 3.80 (3.46 to 4.23) | 9.00 (7.86 to 10.56) | 8.72 (7.59 to 10.31) | 5.46 (4.85 to 6.18) | 5.40 (4.79 to 6.14) | 7.05 (5.15 to 10.09) | 7.25 (5.19 to 10.53) | 4.94 (4.51 to 5.47) | 4.75 (4.33 to 5.27) |
| >6-12 | 6.19 (5.34 to 7.50) | 6.24 (5.42 to 7.51) | 5.71 (4.99 to 6.67) | | 5.40 (4.51 to 6.65) | 4.70 (4.18 to 5.37) | | 4.95 (4.38 to 5.71) | 3.88 (3.47 to 4.39) | 3.85 (3.53 to 4.23) | 8.94 (7.85 to 10.33) | 8.25 (7.12 to 9.77) | 5.49 (4.95 to 6.10) | 5.52 (4.96 to 6.16) | 7.30 (5.16 to 10.75) | 7.93 (5.88 to 11.24) | 5.37 (4.92 to 5.92) | 4.92 (4.53 to 5.40) |
| >12 | 6.20 (5.30 to 7.61) | 6.24 (5.62 to 7.16) | 5.50 (4.61 to 6.76) | | 5.15 (4.05 to 6.78) | 4.80 (4.53 to 5.15) | | 4.96 (4.66 to 5.33) | 3.93 (3.40 to 4.61) | 3.91 (3.47 to 4.48) | 10.45 (9.38 to 11.78) | 9.68 (8.62 to 11.11) | 5.37 (4.68 to 6.29) | 5.44 (4.70 to 6.46) | 7.21 (5.07 to 10.69) | 8.41 (6.60 to 11.20) | 4.85 (4.43 to 5.35) | 4.61 (4.20 to 5.09) |

UI=uncertainty interval.

Education levels are defined as: low education (0-6 years) and high education (>12 years ). The standardized serving size used for this analysis was a cup (8oz). Total green or black tea intake, including caffeinated, decaffeinated, sweetened or unsweetened tea. This definition excludes herbal tea.

*In previous Global Dietary Database reports, the region central or eastern Europe and central Asia was referred to as the former Soviet Union, and southeast and east Asia was referred to as Asia.

**Supplementary Table 6. National mean tea intake (cup (8 oz) per week) in adults aged ≥20 years by sex, age, and area of residence, presented by global and regional estimates stratified by education level across in the 25 most populous countries in 2018.**

|  | **Mean (95% UI)** | | | | | | | | | | | | | | | | | | | | | | | | |
| --- | --- | --- | --- | --- | --- | --- | --- | --- | --- | --- | --- | --- | --- | --- | --- | --- | --- | --- | --- | --- | --- | --- | --- | --- | --- |
|  | **Overall** | | | **Age** | | | | | | | | **Education** | | | | | | | | | **Area of residence** | | | | |
|  |  |  |  | **20-39 years** | | **40-59 years** | | | **≥60 years** | | | **0-6 years** | | | **>6-12 years** | | | **>12 years** | | | **Rural** | | | **Urban** | |
|  | Female | Male | Female | | Male | Female | Male | Female | | Male | Female | | Male | Female | | Male | Female | | Male | Female | | Male | Female | | Male |
| India | 5.22 (4.82 to 5.64) | 5.02 (4.62 to 5.47) | 5.22 (4.82 to 5.64) | | 5.02 (4.62 to 5.47) | 5.92 (5.32 to 6.59) | 5.70 (5.10 to 6.37) | 6.11 (5.09 to 7.35) | | 5.88 (4.87 to 7.10) | 5.11 (4.72 to 5.54) | | 4.92 (4.52 to 5.36) | 5.35 (4.93 to 5.80) | | 5.15 (4.72 to 5.61) | 5.38 (4.94 to 5.84) | | 5.18 (4.73 to 5.66) | 5.21 (4.81 to 5.64) | | 5.02 (4.60 to 5.46) | 5.23 (4.82 to 5.67) | | 5.03 (4.62 to 5.49) |
| China | 8.21 (5.88 to 11.56) | 7.84 (5.66 to 10.81) | 8.21 (5.88 to 11.56) | | 7.84 (5.66 to 10.81) | 8.23 (5.86 to 11.61) | 7.86 (5.67 to 10.86) | 8.06 (5.78 to 11.37) | | 7.67 (5.54 to 10.64) | 8.15 (4.96 to 13.52) | | 7.79 (4.83 to 12.47) | 8.03 (4.92 to 13.10) | | 7.68 (4.78 to 12.26) | 8.02 (4.90 to 13.18) | | 7.67 (4.77 to 12.30) | 8.10 (5.26 to 12.43) | | 7.73 (5.18 to 11.66) | 8.15 (5.33 to 12.62) | | 7.81 (5.16 to 11.82) |
| Nigeria | 3.36 (3.14 to 3.60) | 3.56 (3.31 to 3.83) | 3.36 (3.14 to 3.60) | | 3.56 (3.31 to 3.83) | 3.53 (3.28 to 3.80) | 3.74 (3.46 to 4.05) | 3.50 (3.11 to 3.92) | | 3.70 (3.29 to 4.19) | 3.33 (3.11 to 3.56) | | 3.52 (3.27 to 3.79) | 3.43 (3.21 to 3.67) | | 3.63 (3.38 to 3.91) | 3.55 (3.30 to 3.81) | | 3.76 (3.48 to 4.05) | 3.29 (3.08 to 3.52) | | 3.48 (3.24 to 3.75) | 3.44 (3.22 to 3.69) | | 3.64 (3.38 to 3.92) |
| Pakistan | 4.95 (3.34 to 7.51) | 4.88 (3.33 to 7.28) | 4.95 (3.34 to 7.51) | | 4.88 (3.33 to 7.28) | 5.64 (3.79 to 8.62) | 5.55 (3.78 to 8.34) | 5.82 (3.82 to 9.13) | | 5.75 (3.80 to 8.89) | 4.82 (2.91 to 8.15) | | 4.77 (2.89 to 7.89) | 5.02 (2.99 to 8.53) | | 4.95 (3.05 to 8.14) | 5.06 (3.05 to 8.49) | | 4.99 (3.05 to 8.27) | 4.88 (3.00 to 8.24) | | 4.83 (3.00 to 7.88) | 4.90 (2.99 to 8.09) | | 4.83 (3.01 to 7.90) |
| Indonesia | 4.23 (3.93 to 4.57) | 4.21 (3.91 to 4.52) | 4.23 (3.93 to 4.57) | | 4.21 (3.91 to 4.52) | 4.24 (3.95 to 4.58) | 4.22 (3.92 to 4.54) | 4.15 (3.86 to 4.49) | | 4.12 (3.84 to 4.44) | 4.25 (3.95 to 4.59) | | 4.23 (3.93 to 4.55) | 4.20 (3.91 to 4.54) | | 4.18 (3.88 to 4.50) | 4.19 (3.90 to 4.53) | | 4.17 (3.87 to 4.48) | 4.21 (3.91 to 4.55) | | 4.19 (3.89 to 4.50) | 4.25 (3.95 to 4.59) | | 4.22 (3.93 to 4.54) |
| United States | 4.50 (4.34 to 4.67) | 4.41 (4.25 to 4.58) | 4.50 (4.34 to 4.67) | | 4.41 (4.25 to 4.58) | 5.06 (4.88 to 5.26) | 4.96 (4.78 to 5.15) | 5.18 (4.99 to 5.40) | | 5.08 (4.88 to 5.29) | 4.14 (3.97 to 4.31) | | 4.05 (3.89 to 4.22) | 4.52 (4.35 to 4.69) | | 4.43 (4.26 to 4.60) | 4.52 (4.35 to 4.70) | | 4.43 (4.26 to 4.60) | 4.43 (4.33 to 4.54) | | 4.34 (4.23 to 4.45) | 4.52 (4.33 to 4.71) | | 4.43 (4.24 to 4.62) |
| Brazil | 3.69 (3.38 to 4.02) | 3.66 (3.37 to 3.96) | 3.69 (3.38 to 4.02) | | 3.66 (3.37 to 3.96) | 3.76 (3.45 to 4.10) | 3.73 (3.43 to 4.04) | 3.48 (3.13 to 3.88) | | 3.43 (3.08 to 3.83) | 3.69 (3.37 to 4.03) | | 3.66 (3.36 to 3.97) | 3.69 (3.38 to 4.04) | | 3.66 (3.37 to 3.98) | 3.67 (3.35 to 4.02) | | 3.64 (3.34 to 3.97) | 3.69 (3.36 to 4.05) | | 3.66 (3.35 to 4.00) | 3.69 (3.38 to 4.02) | | 3.66 (3.37 to 3.96) |
| Bangladesh | 3.91 (3.64 to 4.19) | 4.42 (4.02 to 4.90) | 3.91 (3.64 to 4.19) | | 4.42 (4.02 to 4.90) | 4.45 (4.08 to 4.85) | 5.02 (4.53 to 5.56) | 4.59 (3.88 to 5.41) | | 5.19 (4.38 to 6.13) | 3.83 (3.55 to 4.12) | | 4.33 (3.93 to 4.81) | 4.00 (3.72 to 4.30) | | 4.52 (4.10 to 5.02) | 4.03 (3.74 to 4.33) | | 4.55 (4.11 to 5.06) | 3.90 (3.64 to 4.18) | | 4.41 (4.01 to 4.89) | 3.91 (3.62 to 4.23) | | 4.42 (4.00 to 4.93) |
| Ethiopia | 4.59 (4.34 to 4.87) | 3.63 (3.41 to 3.87) | 4.59 (4.34 to 4.87) | | 3.63 (3.41 to 3.87) | 4.83 (4.55 to 5.15) | 3.82 (3.56 to 4.11) | 4.79 (4.30 to 5.34) | | 3.79 (3.36 to 4.24) | 4.54 (4.29 to 4.81) | | 3.59 (3.37 to 3.83) | 4.68 (4.42 to 4.96) | | 3.70 (3.47 to 3.95) | 4.84 (4.54 to 5.15) | | 3.82 (3.57 to 4.11) | 4.55 (4.31 to 4.83) | | 3.60 (3.38 to 3.84) | 4.76 (4.49 to 5.04) | | 3.76 (3.53 to 4.02) |
| Democratic Republic of the Congo | 3.94 (2.80 to 5.77) | 3.89 (2.77 to 5.56) | 3.94 (2.80 to 5.77) | | 3.89 (2.77 to 5.56) | 4.15 (2.94 to 6.05) | 4.08 (2.92 to 5.90) | 4.12 (2.86 to 6.07) | | 4.02 (2.82 to 5.88) | 3.86 (2.43 to 6.24) | | 3.81 (2.44 to 5.99) | 4.00 (2.52 to 6.50) | | 3.92 (2.51 to 6.22) | 4.13 (2.57 to 6.65) | | 4.07 (2.60 to 6.35) | 3.80 (2.39 to 6.40) | | 3.75 (2.42 to 6.11) | 3.98 (2.50 to 6.67) | | 3.93 (2.52 to 6.42) |
| Mexico | 3.56 (3.17 to 3.99) | 3.51 (3.13 to 3.93) | 3.56 (3.17 to 3.99) | | 3.51 (3.13 to 3.93) | 3.64 (3.22 to 4.11) | 3.59 (3.18 to 4.04) | 3.36 (2.89 to 3.90) | | 3.30 (2.86 to 3.83) | 3.56 (3.16 to 4.01) | | 3.51 (3.12 to 3.95) | 3.56 (3.17 to 4.01) | | 3.52 (3.12 to 3.94) | 3.54 (3.15 to 3.99) | | 3.49 (3.10 to 3.93) | 3.56 (3.17 to 4.00) | | 3.51 (3.13 to 3.94) | 3.55 (3.16 to 3.99) | | 3.51 (3.12 to 3.94) |
| Philippines | 4.28 (4.01 to 4.58) | 3.94 (3.66 to 4.24) | 4.28 (4.01 to 4.58) | | 3.94 (3.66 to 4.24) | 4.30 (4.02 to 4.60) | 3.95 (3.67 to 4.26) | 4.21 (3.93 to 4.52) | | 3.87 (3.59 to 4.17) | 4.32 (4.05 to 4.63) | | 3.98 (3.69 to 4.29) | 4.27 (4.00 to 4.57) | | 3.93 (3.65 to 4.24) | 4.26 (3.99 to 4.56) | | 3.92 (3.63 to 4.22) | 4.26 (3.99 to 4.56) | | 3.92 (3.64 to 4.22) | 4.30 (4.03 to 4.60) | | 3.96 (3.67 to 4.26) |
| Egypt | 9.10 (8.34 to 9.93) | 8.97 (8.10 to 9.87) | 9.10 (8.34 to 9.93) | | 8.97 (8.10 to 9.87) | 10.36 (9.45 to 11.40) | 10.21 (9.16 to 11.37) | 10.81 (9.72 to 12.05) | | 10.66 (9.46 to 12.02) | 9.04 (8.27 to 9.88) | | 8.90 (8.04 to 9.84) | 9.04 (8.26 to 9.88) | | 8.90 (8.02 to 9.84) | 9.42 (8.59 to 10.33) | | 9.28 (8.35 to 10.27) | 9.34 (8.54 to 10.22) | | 9.20 (8.30 to 10.16) | 8.78 (8.03 to 9.61) | | 8.66 (7.80 to 9.58) |
| Russia | 5.12 (3.46 to 7.64) | 4.98 (3.40 to 7.35) | 5.12 (3.46 to 7.64) | | 4.98 (3.40 to 7.35) | 5.06 (3.40 to 7.61) | 4.92 (3.35 to 7.30) | 4.82 (3.24 to 7.30) | | 4.67 (3.18 to 6.93) | 5.67 (3.32 to 9.81) | | 5.49 (3.28 to 9.30) | 5.06 (2.97 to 8.71) | | 4.91 (2.97 to 8.32) | 4.89 (2.88 to 8.37) | | 4.78 (2.85 to 8.06) | 5.19 (3.24 to 8.53) | | 5.05 (3.21 to 8.08) | 5.04 (3.14 to 8.11) | | 4.90 (3.10 to 7.80) |
| Tanzania | 4.49 (4.17 to 4.84) | 4.28 (3.92 to 4.66) | 4.49 (4.17 to 4.84) | | 4.28 (3.92 to 4.66) | 4.72 (4.37 to 5.12) | 4.49 (4.10 to 4.93) | 4.68 (4.15 to 5.30) | | 4.45 (3.90 to 5.06) | 4.44 (4.11 to 4.77) | | 4.22 (3.87 to 4.60) | 4.57 (4.25 to 4.93) | | 4.35 (3.99 to 4.75) | 4.73 (4.36 to 5.11) | | 4.50 (4.11 to 4.93) | 4.43 (4.11 to 4.77) | | 4.22 (3.87 to 4.60) | 4.63 (4.30 to 4.99) | | 4.41 (4.04 to 4.81) |
| Vietnam | 8.61 (7.86 to 9.43) | 8.58 (7.83 to 9.41) | 8.61 (7.86 to 9.43) | | 8.58 (7.83 to 9.41) | 8.63 (7.87 to 9.46) | 8.60 (7.85 to 9.45) | 8.45 (7.70 to 9.27) | | 8.41 (7.67 to 9.25) | 8.66 (7.90 to 9.49) | | 8.64 (7.88 to 9.47) | 8.56 (7.81 to 9.37) | | 8.53 (7.79 to 9.36) | 8.54 (7.79 to 9.36) | | 8.52 (7.75 to 9.33) | 8.58 (7.83 to 9.39) | | 8.55 (7.81 to 9.38) | 8.66 (7.89 to 9.49) | | 8.63 (7.88 to 9.47) |
| Turkey | 5.27 (3.44 to 7.96) | 5.44 (3.59 to 8.12) | 5.27 (3.44 to 7.96) | | 5.44 (3.59 to 8.12) | 5.98 (3.88 to 9.08) | 6.18 (4.07 to 9.25) | 6.26 (4.03 to 9.46) | | 6.46 (4.25 to 9.67) | 5.22 (3.41 to 7.91) | | 5.40 (3.57 to 8.08) | 5.22 (3.41 to 7.89) | | 5.40 (3.56 to 8.08) | 5.46 (3.56 to 8.25) | | 5.63 (3.73 to 8.40) | 5.51 (3.59 to 8.32) | | 5.69 (3.75 to 8.50) | 5.18 (3.39 to 7.83) | | 5.35 (3.54 to 7.96) |
| Kenya | 13.01 (11.59 to 14.68) | 12.72 (11.28 to 14.31) | 13.01 (11.59 to 14.68) | | 12.72 (11.28 to 14.31) | 13.66 (12.09 to 15.49) | 13.35 (11.78 to 15.10) | 13.52 (11.58 to 15.77) | | 13.20 (11.31 to 15.40) | 12.84 (11.42 to 14.49) | | 12.54 (11.11 to 14.11) | 13.23 (11.76 to 14.93) | | 12.94 (11.46 to 14.57) | 13.68 (12.14 to 15.47) | | 13.36 (11.84 to 15.12) | 12.86 (11.46 to 14.53) | | 12.57 (11.15 to 14.16) | 13.45 (11.94 to 15.17) | | 13.14 (11.66 to 14.77) |
| Iran | 15.17 (14.21 to 16.25) | 17.52 (16.44 to 18.72) | 15.17 (14.21 to 16.25) | | 17.52 (16.44 to 18.72) | 17.11 (16.09 to 18.25) | 19.77 (18.66 to 21.02) | 17.90 (16.60 to 19.31) | | 20.70 (19.33 to 22.19) | 15.00 (13.98 to 16.15) | | 17.32 (16.16 to 18.62) | 14.99 (14.03 to 16.09) | | 17.31 (16.19 to 18.54) | 15.63 (14.58 to 16.81) | | 18.05 (16.88 to 19.36) | 15.85 (14.69 to 17.17) | | 18.31 (17.02 to 19.76) | 14.92 (13.98 to 15.98) | | 17.23 (16.17 to 18.41) |
| Uganda | 3.43 (3.20 to 3.68) | 3.55 (3.29 to 3.83) | 3.43 (3.20 to 3.68) | | 3.55 (3.29 to 3.83) | 3.61 (3.35 to 3.90) | 3.74 (3.44 to 4.07) | 3.58 (3.18 to 4.03) | | 3.71 (3.27 to 4.20) | 3.39 (3.17 to 3.64) | | 3.52 (3.25 to 3.80) | 3.50 (3.26 to 3.76) | | 3.63 (3.35 to 3.92) | 3.62 (3.36 to 3.90) | | 3.75 (3.45 to 4.06) | 3.39 (3.17 to 3.64) | | 3.52 (3.25 to 3.80) | 3.55 (3.31 to 3.81) | | 3.68 (3.40 to 3.97) |
| Japan | 16.31 (13.63 to 19.51) | 13.75 (11.45 to 16.43) | 16.31 (13.63 to 19.51) | | 13.75 (11.45 to 16.43) | 16.35 (13.65 to 19.54) | 13.78 (11.49 to 16.44) | 15.95 (13.31 to 19.05) | | 13.40 (11.17 to 15.94) | 16.51 (13.78 to 19.80) | | 13.92 (11.57 to 16.61) | 16.32 (13.62 to 19.49) | | 13.74 (11.44 to 16.41) | 16.28 (13.61 to 19.45) | | 13.72 (11.42 to 16.39) | 16.18 (13.53 to 19.35) | | 13.64 (11.34 to 16.26) | 16.33 (13.64 to 19.52) | | 13.76 (11.46 to 16.44) |
| South Africa | 3.32 (3.06 to 3.60) | 3.32 (3.07 to 3.58) | 3.32 (3.06 to 3.60) | | 3.32 (3.07 to 3.58) | 3.47 (3.18 to 3.80) | 3.47 (3.20 to 3.77) | 3.44 (3.03 to 3.91) | | 3.43 (3.03 to 3.90) | 3.24 (2.98 to 3.52) | | 3.24 (2.99 to 3.50) | 3.34 (3.08 to 3.63) | | 3.34 (3.09 to 3.61) | 3.45 (3.17 to 3.76) | | 3.45 (3.18 to 3.73) | 3.22 (2.98 to 3.50) | | 3.22 (2.98 to 3.48) | 3.37 (3.10 to 3.66) | | 3.37 (3.12 to 3.64) |
| Sudan | 12.96 (9.07 to 19.06) | 12.71 (8.93 to 18.59) | 12.96 (9.07 to 19.06) | | 12.71 (8.93 to 18.59) | 13.61 (9.45 to 20.26) | 13.35 (9.36 to 19.51) | 13.50 (9.24 to 20.17) | | 13.27 (9.16 to 19.46) | 12.67 (7.96 to 20.87) | | 12.47 (7.81 to 20.16) | 13.13 (8.04 to 21.35) | | 12.83 (8.07 to 20.52) | 13.54 (8.38 to 22.07) | | 13.26 (8.39 to 21.59) | 12.60 (7.87 to 20.83) | | 12.37 (7.81 to 20.18) | 13.19 (8.25 to 21.69) | | 12.94 (8.25 to 20.88) |
| Afghanistan | 12.92 (8.00 to 21.57) | 12.78 (8.07 to 20.93) | 12.92 (8.00 to 21.57) | | 12.78 (8.07 to 20.93) | 14.74 (9.19 to 24.60) | 14.57 (9.17 to 23.91) | 15.32 (9.26 to 26.18) | | 15.11 (9.30 to 25.20) | 12.77 (7.39 to 22.44) | | 12.62 (7.41 to 21.65) | 13.40 (7.79 to 23.59) | | 13.22 (7.85 to 22.76) | 13.47 (7.83 to 23.71) | | 13.28 (7.87 to 22.86) | 12.79 (7.11 to 23.44) | | 12.60 (7.18 to 22.47) | 12.76 (7.17 to 23.59) | | 12.63 (7.23 to 22.60) |
| Myanmar | 7.33 (5.01 to 11.11) | 7.01 (4.82 to 10.48) | 7.33 (5.01 to 11.11) | | 7.01 (4.82 to 10.48) | 7.36 (4.96 to 11.23) | 7.03 (4.81 to 10.56) | 7.21 (4.82 to 10.98) | | 6.87 (4.71 to 10.26) | 7.27 (4.36 to 12.27) | | 6.96 (4.25 to 11.56) | 7.19 (4.32 to 12.11) | | 6.87 (4.17 to 11.27) | 7.17 (4.37 to 12.18) | | 6.86 (4.22 to 11.39) | 7.22 (4.49 to 12.01) | | 6.92 (4.32 to 11.34) | 7.32 (4.50 to 12.33) | | 6.98 (4.38 to 11.45) |

UI=uncertainty interval.

Education levels are defined as: low education (0-6 years) and high education (>12 years ). The standardized serving size used for this analysis was a cup (8oz). Total green or black tea intake, including caffeinated, decaffeinated, sweetened or unsweetened tea. This definition excludes herbal tea.

*In previous Global Dietary Database reports, the region central or eastern Europe and central Asia was referred to as the former Soviet Union, and southeast and east Asia was referred to as Asia.

**Supplementary Table 7. Mean tea intake (cup (8 oz) per week) in adults aged ≥20 years by sex, age, and education, presented by global and regional estimates stratified by area of residence across 185 countries in 2018.**

|  | **Mean (95% UI)** | | | | | | | | | | | | | | | | | | | | | |
| --- | --- | --- | --- | --- | --- | --- | --- | --- | --- | --- | --- | --- | --- | --- | --- | --- | --- | --- | --- | --- | --- | --- |
|  | **Overall** | | | **Sex** | | | | | **Age** | | | | | | | | | **Area of residence** | | | | |
|  |  |  |  | **Female** | | **Male** | | | **20-39 years** | | | **40-59 years** | | | **≥60 years** | | | **Rural** | | | **Urban** | |
|  | Low | High | Low | | High | Low | High | Low | | High | Low | | High | Low | | High | Low | | High | Low | | High |
| India | 5.01 (4.63 to 5.42) | 5.27 (4.85 to 5.73) | 5.46 (4.92 to 6.07) | | 5.75 (5.15 to 6.41) | 5.28 (4.72 to 5.90) | 5.55 (4.94 to 6.24) | 5.01 (4.63 to 5.42) | | 5.27 (4.85 to 5.73) | 5.70 (5.11 to 6.32) | | 5.99 (5.36 to 6.69) | 5.88 (4.89 to 7.04) | | 6.19 (5.13 to 7.46) | 5.36 (4.82 to 5.96) | | 5.64 (5.04 to 6.30) | 5.38 (4.83 to 5.99) | | 5.66 (5.06 to 6.32) |
| China | 8.04 (5.56 to 11.73) | 7.94 (5.48 to 11.53) | 8.12 (4.94 to 13.41) | | 8.01 (4.88 to 13.11) | 7.75 (4.80 to 12.42) | 7.64 (4.77 to 12.32) | 8.04 (5.56 to 11.73) | | 7.94 (5.48 to 11.53) | 8.08 (5.52 to 11.72) | | 7.97 (5.51 to 11.59) | 7.91 (5.41 to 11.57) | | 7.80 (5.40 to 11.36) | 7.89 (4.85 to 12.82) | | 7.78 (4.81 to 12.65) | 7.98 (4.92 to 12.90) | | 7.87 (4.87 to 12.73) |
| Nigeria | 3.43 (3.21 to 3.66) | 3.65 (3.40 to 3.91) | 3.39 (3.15 to 3.65) | | 3.61 (3.34 to 3.90) | 3.59 (3.31 to 3.89) | 3.83 (3.52 to 4.16) | 3.43 (3.21 to 3.66) | | 3.65 (3.40 to 3.91) | 3.60 (3.35 to 3.86) | | 3.83 (3.55 to 4.13) | 3.56 (3.18 to 3.99) | | 3.79 (3.37 to 4.27) | 3.42 (3.18 to 3.67) | | 3.64 (3.37 to 3.93) | 3.57 (3.32 to 3.85) | | 3.80 (3.52 to 4.11) |
| Pakistan | 4.87 (3.28 to 7.19) | 5.09 (3.44 to 7.61) | 5.10 (3.06 to 8.69) | | 5.36 (3.21 to 9.08) | 5.06 (3.06 to 8.43) | 5.30 (3.22 to 8.82) | 4.87 (3.28 to 7.19) | | 5.09 (3.44 to 7.61) | 5.52 (3.70 to 8.30) | | 5.79 (3.91 to 8.71) | 5.71 (3.73 to 8.80) | | 6.00 (3.91 to 9.27) | 5.09 (3.10 to 8.43) | | 5.34 (3.25 to 8.83) | 5.10 (3.08 to 8.40) | | 5.36 (3.25 to 8.89) |
| Indonesia | 4.24 (3.98 to 4.54) | 4.18 (3.91 to 4.48) | 4.25 (3.94 to 4.59) | | 4.19 (3.89 to 4.53) | 4.22 (3.93 to 4.54) | 4.16 (3.86 to 4.47) | 4.24 (3.98 to 4.54) | | 4.18 (3.91 to 4.48) | 4.26 (3.99 to 4.55) | | 4.19 (3.92 to 4.49) | 4.16 (3.90 to 4.46) | | 4.10 (3.84 to 4.40) | 4.21 (3.95 to 4.51) | | 4.15 (3.89 to 4.45) | 4.25 (3.98 to 4.55) | | 4.19 (3.92 to 4.49) |
| United States | 4.09 (3.94 to 4.26) | 4.47 (4.31 to 4.64) | 4.49 (4.31 to 4.67) | | 4.90 (4.71 to 5.10) | 4.41 (4.23 to 4.60) | 4.82 (4.63 to 5.01) | 4.09 (3.94 to 4.26) | | 4.47 (4.31 to 4.64) | 4.61 (4.43 to 4.79) | | 5.03 (4.85 to 5.22) | 4.72 (4.54 to 4.91) | | 5.15 (4.96 to 5.37) | 4.38 (4.27 to 4.50) | | 4.79 (4.67 to 4.91) | 4.47 (4.27 to 4.67) | | 4.87 (4.68 to 5.09) |
| Brazil | 3.68 (3.42 to 3.95) | 3.66 (3.39 to 3.94) | 3.68 (3.36 to 4.03) | | 3.66 (3.33 to 4.03) | 3.64 (3.32 to 3.97) | 3.62 (3.30 to 3.97) | 3.68 (3.42 to 3.95) | | 3.66 (3.39 to 3.94) | 3.75 (3.50 to 4.02) | | 3.73 (3.46 to 4.02) | 3.46 (3.14 to 3.82) | | 3.44 (3.11 to 3.82) | 3.66 (3.37 to 3.97) | | 3.65 (3.33 to 3.99) | 3.66 (3.39 to 3.95) | | 3.64 (3.36 to 3.94) |
| Bangladesh | 4.08 (3.79 to 4.40) | 4.29 (3.98 to 4.63) | 4.09 (3.72 to 4.49) | | 4.30 (3.91 to 4.72) | 4.62 (4.14 to 5.18) | 4.86 (4.33 to 5.45) | 4.08 (3.79 to 4.40) | | 4.29 (3.98 to 4.63) | 4.64 (4.27 to 5.05) | | 4.88 (4.48 to 5.32) | 4.80 (4.10 to 5.62) | | 5.05 (4.28 to 5.92) | 4.35 (3.99 to 4.77) | | 4.58 (4.18 to 5.02) | 4.37 (3.97 to 4.82) | | 4.59 (4.16 to 5.07) |
| Ethiopia | 4.06 (3.86 to 4.29) | 4.33 (4.09 to 4.60) | 4.62 (4.34 to 4.93) | | 4.93 (4.60 to 5.28) | 3.66 (3.41 to 3.93) | 3.90 (3.61 to 4.22) | 4.06 (3.86 to 4.29) | | 4.33 (4.09 to 4.60) | 4.29 (4.04 to 4.56) | | 4.57 (4.28 to 4.88) | 4.28 (3.83 to 4.75) | | 4.55 (4.06 to 5.08) | 4.11 (3.87 to 4.37) | | 4.38 (4.10 to 4.68) | 4.30 (4.04 to 4.58) | | 4.58 (4.28 to 4.90) |
| Democratic Republic of the Congo | 3.88 (2.78 to 5.47) | 4.15 (2.99 to 5.84) | 3.94 (2.47 to 6.35) | | 4.21 (2.63 to 6.79) | 3.88 (2.48 to 6.11) | 4.15 (2.64 to 6.50) | 3.88 (2.78 to 5.47) | | 4.15 (2.99 to 5.84) | 4.09 (2.92 to 5.76) | | 4.37 (3.13 to 6.18) | 4.05 (2.85 to 5.80) | | 4.32 (3.03 to 6.20) | 3.84 (2.43 to 6.11) | | 4.08 (2.61 to 6.47) | 4.01 (2.55 to 6.35) | | 4.29 (2.72 to 6.83) |
| Mexico | 3.54 (3.21 to 3.91) | 3.52 (3.19 to 3.89) | 3.55 (3.13 to 4.03) | | 3.54 (3.12 to 4.01) | 3.50 (3.09 to 3.97) | 3.49 (3.08 to 3.95) | 3.54 (3.21 to 3.91) | | 3.52 (3.19 to 3.89) | 3.62 (3.26 to 4.02) | | 3.60 (3.23 to 4.01) | 3.34 (2.91 to 3.83) | | 3.32 (2.90 to 3.81) | 3.53 (3.18 to 3.94) | | 3.52 (3.15 to 3.93) | 3.53 (3.17 to 3.94) | | 3.52 (3.15 to 3.92) |
| Philippines | 4.15 (3.89 to 4.44) | 4.09 (3.83 to 4.37) | 4.32 (4.04 to 4.63) | | 4.26 (3.98 to 4.56) | 3.97 (3.69 to 4.28) | 3.92 (3.63 to 4.22) | 4.15 (3.89 to 4.44) | | 4.09 (3.83 to 4.37) | 4.17 (3.91 to 4.46) | | 4.11 (3.85 to 4.39) | 4.10 (3.84 to 4.39) | | 4.04 (3.78 to 4.33) | 4.13 (3.87 to 4.42) | | 4.07 (3.81 to 4.35) | 4.17 (3.90 to 4.46) | | 4.11 (3.85 to 4.39) |
| Egypt | 8.97 (8.18 to 9.83) | 9.35 (8.50 to 10.27) | 9.64 (8.78 to 10.60) | | 10.05 (9.12 to 11.09) | 9.54 (8.57 to 10.61) | 9.94 (8.89 to 11.08) | 8.97 (8.18 to 9.83) | | 9.35 (8.50 to 10.27) | 10.21 (9.26 to 11.29) | | 10.64 (9.61 to 11.82) | 10.67 (9.55 to 11.94) | | 11.11 (9.90 to 12.52) | 9.84 (8.91 to 10.87) | | 10.26 (9.25 to 11.37) | 9.26 (8.39 to 10.23) | | 9.65 (8.72 to 10.69) |
| Russia | 5.66 (3.75 to 8.58) | 4.91 (3.29 to 7.38) | 5.56 (3.25 to 9.64) | | 4.82 (2.81 to 8.28) | 5.37 (3.21 to 9.09) | 4.67 (2.77 to 7.87) | 5.66 (3.75 to 8.58) | | 4.91 (3.29 to 7.38) | 5.62 (3.72 to 8.50) | | 4.86 (3.20 to 7.35) | 5.31 (3.50 to 8.23) | | 4.64 (3.05 to 7.20) | 5.62 (3.41 to 9.28) | | 4.89 (2.97 to 8.06) | 5.46 (3.30 to 9.06) | | 4.73 (2.86 to 7.79) |
| Tanzania | 4.33 (4.03 to 4.66) | 4.62 (4.27 to 4.98) | 4.51 (4.17 to 4.89) | | 4.81 (4.42 to 5.24) | 4.30 (3.91 to 4.72) | 4.58 (4.16 to 5.05) | 4.33 (4.03 to 4.66) | | 4.62 (4.27 to 4.98) | 4.55 (4.21 to 4.92) | | 4.85 (4.47 to 5.27) | 4.52 (4.02 to 5.10) | | 4.82 (4.26 to 5.47) | 4.35 (4.02 to 4.70) | | 4.63 (4.26 to 5.04) | 4.54 (4.20 to 4.92) | | 4.84 (4.45 to 5.26) |
| Vietnam | 8.65 (7.95 to 9.41) | 8.53 (7.83 to 9.27) | 8.65 (7.89 to 9.48) | | 8.53 (7.77 to 9.35) | 8.61 (7.85 to 9.46) | 8.49 (7.73 to 9.32) | 8.65 (7.95 to 9.41) | | 8.53 (7.83 to 9.27) | 8.67 (7.96 to 9.44) | | 8.55 (7.85 to 9.29) | 8.48 (7.80 to 9.25) | | 8.36 (7.67 to 9.12) | 8.61 (7.90 to 9.37) | | 8.48 (7.79 to 9.22) | 8.68 (7.98 to 9.46) | | 8.56 (7.86 to 9.32) |
| Turkey | 5.31 (3.56 to 7.90) | 5.55 (3.70 to 8.19) | 5.63 (3.66 to 8.52) | | 5.88 (3.82 to 8.89) | 5.84 (3.85 to 8.75) | 6.09 (4.02 to 9.09) | 5.31 (3.56 to 7.90) | | 5.55 (3.70 to 8.19) | 6.03 (4.01 to 9.02) | | 6.31 (4.18 to 9.34) | 6.31 (4.16 to 9.47) | | 6.58 (4.33 to 9.80) | 6.00 (3.97 to 8.92) | | 6.27 (4.14 to 9.26) | 5.64 (3.76 to 8.42) | | 5.90 (3.91 to 8.75) |
| Kenya | 12.69 (11.36 to 14.20) | 13.52 (12.09 to 15.19) | 13.06 (11.56 to 14.79) | | 13.91 (12.29 to 15.80) | 12.76 (11.26 to 14.43) | 13.60 (11.98 to 15.44) | 12.69 (11.36 to 14.20) | | 13.52 (12.09 to 15.19) | 13.32 (11.86 to 15.00) | | 14.20 (12.63 to 16.02) | 13.20 (11.37 to 15.34) | | 14.07 (12.08 to 16.36) | 12.76 (11.36 to 14.35) | | 13.60 (12.10 to 15.34) | 13.34 (11.86 to 15.02) | | 14.22 (12.64 to 16.03) |
| Iran | 16.16 (15.19 to 17.24) | 16.83 (15.87 to 17.94) | 16.01 (14.93 to 17.22) | | 16.68 (15.55 to 17.95) | 18.52 (17.31 to 19.88) | 19.30 (18.06 to 20.68) | 16.16 (15.19 to 17.24) | | 16.83 (15.87 to 17.94) | 18.24 (17.26 to 19.35) | | 19.00 (18.01 to 20.14) | 19.11 (17.93 to 20.44) | | 19.92 (18.59 to 21.41) | 18.04 (16.83 to 19.44) | | 18.80 (17.53 to 20.26) | 16.99 (15.99 to 18.09) | | 17.70 (16.70 to 18.83) |
| Uganda | 3.45 (3.23 to 3.70) | 3.68 (3.42 to 3.96) | 3.45 (3.20 to 3.72) | | 3.68 (3.40 to 3.98) | 3.58 (3.29 to 3.89) | 3.81 (3.49 to 4.16) | 3.45 (3.23 to 3.70) | | 3.68 (3.42 to 3.96) | 3.64 (3.38 to 3.92) | | 3.88 (3.59 to 4.20) | 3.60 (3.20 to 4.04) | | 3.83 (3.39 to 4.33) | 3.48 (3.23 to 3.74) | | 3.70 (3.43 to 4.01) | 3.63 (3.37 to 3.92) | | 3.87 (3.58 to 4.19) |
| Japan | 15.20 (12.93 to 17.91) | 14.99 (12.78 to 17.57) | 16.38 (13.66 to 19.62) | | 16.16 (13.49 to 19.29) | 13.77 (11.45 to 16.41) | 13.58 (11.32 to 16.18) | 15.20 (12.93 to 17.91) | | 14.99 (12.78 to 17.57) | 15.25 (12.97 to 17.94) | | 15.03 (12.82 to 17.63) | 15.00 (12.75 to 17.63) | | 14.80 (12.60 to 17.35) | 15.01 (12.77 to 17.66) | | 14.80 (12.61 to 17.37) | 15.15 (12.88 to 17.82) | | 14.94 (12.73 to 17.52) |
| South Africa | 3.24 (3.02 to 3.48) | 3.45 (3.20 to 3.71) | 3.30 (3.02 to 3.61) | | 3.51 (3.20 to 3.85) | 3.30 (3.03 to 3.61) | 3.52 (3.22 to 3.85) | 3.24 (3.02 to 3.48) | | 3.45 (3.20 to 3.71) | 3.39 (3.14 to 3.67) | | 3.61 (3.33 to 3.92) | 3.35 (2.97 to 3.79) | | 3.57 (3.15 to 4.05) | 3.21 (2.96 to 3.48) | | 3.42 (3.14 to 3.71) | 3.35 (3.09 to 3.64) | | 3.57 (3.28 to 3.88) |
| Sudan | 12.74 (9.03 to 18.25) | 13.59 (9.55 to 19.54) | 12.91 (8.05 to 21.28) | | 13.78 (8.51 to 22.66) | 12.70 (7.97 to 20.53) | 13.51 (8.49 to 21.93) | 12.74 (9.03 to 18.25) | | 13.59 (9.55 to 19.54) | 13.37 (9.48 to 19.16) | | 14.27 (9.99 to 20.63) | 13.27 (9.29 to 19.33) | | 14.10 (9.82 to 20.63) | 12.64 (7.99 to 20.05) | | 13.49 (8.52 to 21.49) | 13.23 (8.35 to 20.93) | | 14.09 (8.86 to 22.49) |
| Afghanistan | 12.93 (8.56 to 19.79) | 13.58 (8.99 to 20.96) | 13.41 (7.78 to 23.60) | | 14.13 (8.21 to 24.94) | 13.32 (7.83 to 22.92) | 14.04 (8.28 to 24.18) | 12.93 (8.56 to 19.79) | | 13.58 (8.99 to 20.96) | 14.73 (9.70 to 22.53) | | 15.52 (10.25 to 23.81) | 15.27 (9.83 to 24.07) | | 16.10 (10.30 to 25.46) | 13.48 (8.15 to 22.23) | | 14.18 (8.58 to 23.42) | 13.49 (8.17 to 22.33) | | 14.23 (8.60 to 23.79) |
| Myanmar | 7.22 (4.96 to 10.80) | 7.13 (4.89 to 10.61) | 7.26 (4.34 to 12.28) | | 7.16 (4.33 to 12.18) | 6.93 (4.24 to 11.53) | 6.85 (4.19 to 11.35) | 7.22 (4.96 to 10.80) | | 7.13 (4.89 to 10.61) | 7.24 (4.93 to 10.76) | | 7.14 (4.85 to 10.64) | 7.13 (4.79 to 10.65) | | 7.02 (4.75 to 10.45) | 7.11 (4.39 to 11.61) | | 7.04 (4.33 to 11.45) | 7.20 (4.45 to 11.74) | | 7.09 (4.38 to 11.50) |

UI=uncertainty interval.

The standardized serving size used for this analysis was a cup (8oz). Total green or black tea intake, including caffeinated, decaffeinated, sweetened or unsweetened tea. This definition excludes herbal tea.

*In previous Global Dietary Database reports, the region central or eastern Europe and central Asia was referred to as the former Soviet Union, and southeast and east Asia was referred to as Asia.

**Supplementary Table 8. National mean tea intake (cup (8 oz) per week) in adults aged ≥20 years by sex, age, and education, presented by global and regional estimates stratified by area of residence across in the 25 most populous countries in 2018.**

|  | **Mean (95% UI)** | | | | | | | | | | | | | | | | | | | | | | | | |
| --- | --- | --- | --- | --- | --- | --- | --- | --- | --- | --- | --- | --- | --- | --- | --- | --- | --- | --- | --- | --- | --- | --- | --- | --- | --- |
|  | **Overall** | | | **Sex** | | | | | **Age** | | | | | | | | | **Education** | | | | | | | |
|  |  |  |  | **Female** | | **Male** | | | **20-39 years** | | | **40-59 years** | | | **≥60 years** | | | **0-6 years** | | | **>6-12 years** | | | **>12 years** | |
|  | Rural | Urban | Rural | | Urban | Rural | Urban | Rural | | Urban | Rural | | Urban | Rural | | Urban | Rural | | Urban | Rural | | Urban | Rural | | Urban |
| India | 5.47 (4.92 to 6.08) | 5.49 (4.93 to 6.11) | 5.57 (5.01 to 6.19) | | 5.58 (5.02 to 6.22) | 5.38 (4.80 to 6.02) | 5.39 (4.81 to 6.05) | 5.11 (4.72 to 5.52) | | 5.12 (4.74 to 5.56) | 5.80 (5.21 to 6.46) | | 5.82 (5.22 to 6.49) | 6.00 (4.99 to 7.21) | | 6.02 (4.99 to 7.24) | 5.36 (4.82 to 5.96) | | 5.38 (4.83 to 5.99) | 5.61 (5.03 to 6.25) | | 5.62 (5.04 to 6.29) | 5.64 (5.04 to 6.30) | | 5.66 (5.06 to 6.32) |
| China | 7.94 (5.72 to 11.06) | 8.03 (5.79 to 11.18) | 8.06 (5.27 to 12.41) | | 8.15 (5.30 to 12.57) | 7.68 (5.12 to 11.62) | 7.78 (5.16 to 11.76) | 7.97 (5.73 to 11.09) | | 8.04 (5.83 to 11.23) | 7.99 (5.76 to 11.10) | | 8.08 (5.81 to 11.23) | 7.82 (5.63 to 10.94) | | 7.93 (5.68 to 10.99) | 7.89 (4.85 to 12.82) | | 7.98 (4.92 to 12.90) | 7.79 (4.80 to 12.65) | | 7.87 (4.87 to 12.77) | 7.78 (4.81 to 12.65) | | 7.87 (4.87 to 12.73) |
| Nigeria | 3.45 (3.21 to 3.71) | 3.61 (3.36 to 3.89) | 3.35 (3.11 to 3.61) | | 3.50 (3.25 to 3.78) | 3.55 (3.28 to 3.85) | 3.71 (3.43 to 4.03) | 3.39 (3.18 to 3.62) | | 3.54 (3.32 to 3.79) | 3.56 (3.32 to 3.82) | | 3.72 (3.46 to 4.00) | 3.52 (3.14 to 3.95) | | 3.68 (3.28 to 4.13) | 3.42 (3.18 to 3.67) | | 3.57 (3.32 to 3.85) | 3.52 (3.28 to 3.79) | | 3.68 (3.42 to 3.97) | 3.64 (3.37 to 3.93) | | 3.80 (3.52 to 4.11) |
| Pakistan | 5.22 (3.55 to 7.81) | 5.24 (3.56 to 7.78) | 5.17 (3.17 to 8.77) | | 5.19 (3.16 to 8.67) | 5.12 (3.19 to 8.44) | 5.14 (3.19 to 8.46) | 4.92 (3.37 to 7.29) | | 4.94 (3.37 to 7.27) | 5.59 (3.81 to 8.41) | | 5.62 (3.83 to 8.33) | 5.78 (3.84 to 8.87) | | 5.80 (3.85 to 8.94) | 5.09 (3.10 to 8.43) | | 5.10 (3.08 to 8.40) | 5.31 (3.23 to 8.79) | | 5.32 (3.25 to 8.80) | 5.34 (3.25 to 8.83) | | 5.36 (3.25 to 8.89) |
| Indonesia | 4.19 (3.93 to 4.48) | 4.23 (3.96 to 4.52) | 4.20 (3.91 to 4.55) | | 4.24 (3.94 to 4.58) | 4.18 (3.88 to 4.50) | 4.22 (3.92 to 4.53) | 4.20 (3.93 to 4.49) | | 4.24 (3.97 to 4.53) | 4.21 (3.94 to 4.50) | | 4.25 (3.98 to 4.54) | 4.12 (3.86 to 4.42) | | 4.16 (3.90 to 4.45) | 4.21 (3.95 to 4.51) | | 4.25 (3.98 to 4.55) | 4.16 (3.90 to 4.46) | | 4.20 (3.93 to 4.49) | 4.15 (3.89 to 4.45) | | 4.19 (3.92 to 4.49) |
| United States | 4.77 (4.66 to 4.88) | 4.86 (4.66 to 5.07) | 4.81 (4.69 to 4.93) | | 4.90 (4.70 to 5.11) | 4.73 (4.60 to 4.85) | 4.82 (4.61 to 5.03) | 4.39 (4.29 to 4.49) | | 4.47 (4.29 to 4.66) | 4.94 (4.83 to 5.05) | | 5.03 (4.83 to 5.25) | 5.06 (4.93 to 5.19) | | 5.15 (4.94 to 5.39) | 4.38 (4.27 to 4.50) | | 4.47 (4.27 to 4.67) | 4.79 (4.67 to 4.91) | | 4.88 (4.68 to 5.09) | 4.79 (4.67 to 4.91) | | 4.87 (4.68 to 5.09) |
| Brazil | 3.66 (3.38 to 3.97) | 3.66 (3.40 to 3.94) | 3.68 (3.34 to 4.06) | | 3.68 (3.36 to 4.02) | 3.64 (3.32 to 3.99) | 3.64 (3.33 to 3.96) | 3.68 (3.40 to 3.97) | | 3.67 (3.43 to 3.93) | 3.75 (3.48 to 4.05) | | 3.75 (3.50 to 4.01) | 3.46 (3.13 to 3.83) | | 3.46 (3.15 to 3.81) | 3.66 (3.37 to 3.97) | | 3.66 (3.39 to 3.95) | 3.67 (3.37 to 3.99) | | 3.66 (3.40 to 3.96) | 3.65 (3.33 to 3.99) | | 3.64 (3.36 to 3.94) |
| Bangladesh | 4.44 (4.07 to 4.85) | 4.46 (4.06 to 4.91) | 4.17 (3.81 to 4.56) | | 4.18 (3.79 to 4.61) | 4.71 (4.23 to 5.26) | 4.72 (4.21 to 5.32) | 4.16 (3.88 to 4.47) | | 4.17 (3.86 to 4.52) | 4.73 (4.37 to 5.13) | | 4.75 (4.35 to 5.19) | 4.90 (4.17 to 5.72) | | 4.91 (4.17 to 5.78) | 4.35 (3.99 to 4.77) | | 4.37 (3.97 to 4.82) | 4.55 (4.17 to 4.99) | | 4.57 (4.14 to 5.05) | 4.58 (4.18 to 5.02) | | 4.59 (4.16 to 5.07) |
| Ethiopia | 4.16 (3.92 to 4.42) | 4.35 (4.09 to 4.63) | 4.64 (4.36 to 4.95) | | 4.84 (4.55 to 5.17) | 3.67 (3.41 to 3.94) | 3.83 (3.57 to 4.13) | 4.07 (3.87 to 4.30) | | 4.26 (4.04 to 4.50) | 4.30 (4.05 to 4.57) | | 4.50 (4.23 to 4.78) | 4.29 (3.84 to 4.76) | | 4.48 (4.01 to 4.99) | 4.11 (3.87 to 4.37) | | 4.30 (4.04 to 4.58) | 4.24 (3.99 to 4.51) | | 4.43 (4.17 to 4.72) | 4.38 (4.10 to 4.68) | | 4.58 (4.28 to 4.90) |
| Democratic Republic of the Congo | 3.91 (2.77 to 5.69) | 4.09 (2.91 to 5.93) | 3.88 (2.43 to 6.50) | | 4.05 (2.55 to 6.80) | 3.82 (2.45 to 6.25) | 3.99 (2.56 to 6.54) | 3.84 (2.73 to 5.57) | | 4.02 (2.85 to 5.81) | 4.04 (2.86 to 5.87) | | 4.22 (3.01 to 6.10) | 4.00 (2.78 to 5.88) | | 4.18 (2.93 to 6.12) | 3.84 (2.43 to 6.11) | | 4.01 (2.55 to 6.35) | 3.97 (2.50 to 6.32) | | 4.13 (2.62 to 6.60) | 4.08 (2.61 to 6.47) | | 4.29 (2.72 to 6.83) |
| Mexico | 3.53 (3.18 to 3.93) | 3.53 (3.18 to 3.93) | 3.55 (3.14 to 4.03) | | 3.55 (3.14 to 4.02) | 3.51 (3.10 to 3.96) | 3.50 (3.10 to 3.95) | 3.54 (3.21 to 3.91) | | 3.53 (3.21 to 3.90) | 3.62 (3.26 to 4.02) | | 3.62 (3.26 to 4.01) | 3.33 (2.91 to 3.82) | | 3.33 (2.91 to 3.82) | 3.53 (3.18 to 3.94) | | 3.53 (3.17 to 3.94) | 3.54 (3.18 to 3.94) | | 3.54 (3.17 to 3.93) | 3.52 (3.15 to 3.93) | | 3.52 (3.15 to 3.92) |
| Philippines | 4.09 (3.83 to 4.38) | 4.13 (3.87 to 4.42) | 4.26 (3.99 to 4.56) | | 4.30 (4.02 to 4.60) | 3.92 (3.64 to 4.22) | 3.95 (3.67 to 4.26) | 4.09 (3.84 to 4.37) | | 4.13 (3.87 to 4.42) | 4.11 (3.85 to 4.40) | | 4.15 (3.89 to 4.44) | 4.04 (3.78 to 4.33) | | 4.08 (3.82 to 4.37) | 4.13 (3.87 to 4.42) | | 4.17 (3.90 to 4.46) | 4.08 (3.82 to 4.37) | | 4.12 (3.86 to 4.41) | 4.07 (3.81 to 4.35) | | 4.11 (3.85 to 4.39) |
| Egypt | 9.92 (8.98 to 10.93) | 9.33 (8.46 to 10.28) | 9.97 (9.06 to 10.96) | | 9.38 (8.54 to 10.31) | 9.86 (8.84 to 10.96) | 9.28 (8.32 to 10.32) | 9.27 (8.44 to 10.15) | | 8.72 (7.95 to 9.56) | 10.56 (9.55 to 11.67) | | 9.93 (8.99 to 10.98) | 11.03 (9.83 to 12.35) | | 10.37 (9.29 to 11.62) | 9.84 (8.91 to 10.87) | | 9.26 (8.39 to 10.23) | 9.84 (8.88 to 10.89) | | 9.26 (8.37 to 10.24) | 10.26 (9.25 to 11.37) | | 9.65 (8.72 to 10.69) |
| Russia | 5.08 (3.52 to 7.40) | 4.93 (3.40 to 7.18) | 5.10 (3.19 to 8.39) | | 4.95 (3.08 to 8.02) | 4.92 (3.11 to 7.88) | 4.78 (3.03 to 7.62) | 5.19 (3.63 to 7.47) | | 5.03 (3.50 to 7.25) | 5.12 (3.55 to 7.49) | | 4.97 (3.42 to 7.23) | 4.87 (3.32 to 7.20) | | 4.74 (3.25 to 7.03) | 5.62 (3.41 to 9.28) | | 5.46 (3.30 to 9.06) | 5.01 (3.04 to 8.24) | | 4.88 (2.95 to 8.07) | 4.89 (2.97 to 8.06) | | 4.73 (2.86 to 7.79) |
| Tanzania | 4.40 (4.07 to 4.77) | 4.60 (4.25 to 4.98) | 4.51 (4.16 to 4.88) | | 4.71 (4.35 to 5.11) | 4.29 (3.91 to 4.71) | 4.49 (4.09 to 4.93) | 4.33 (4.02 to 4.65) | | 4.52 (4.21 to 4.86) | 4.55 (4.21 to 4.92) | | 4.75 (4.39 to 5.15) | 4.51 (4.01 to 5.10) | | 4.72 (4.19 to 5.33) | 4.35 (4.02 to 4.70) | | 4.54 (4.20 to 4.92) | 4.48 (4.14 to 4.86) | | 4.69 (4.33 to 5.07) | 4.63 (4.26 to 5.04) | | 4.84 (4.45 to 5.26) |
| Vietnam | 8.55 (7.85 to 9.30) | 8.63 (7.94 to 9.39) | 8.57 (7.81 to 9.38) | | 8.65 (7.88 to 9.48) | 8.53 (7.78 to 9.36) | 8.61 (7.86 to 9.45) | 8.57 (7.87 to 9.32) | | 8.65 (7.96 to 9.40) | 8.59 (7.89 to 9.35) | | 8.67 (7.98 to 9.43) | 8.40 (7.72 to 9.16) | | 8.48 (7.80 to 9.25) | 8.61 (7.90 to 9.37) | | 8.68 (7.98 to 9.46) | 8.50 (7.81 to 9.25) | | 8.58 (7.89 to 9.35) | 8.48 (7.79 to 9.22) | | 8.56 (7.86 to 9.32) |
| Turkey | 6.06 (4.01 to 8.98) | 5.69 (3.79 to 8.46) | 5.94 (3.85 to 8.96) | | 5.58 (3.64 to 8.45) | 6.16 (4.05 to 9.21) | 5.79 (3.82 to 8.65) | 5.61 (3.72 to 8.31) | | 5.26 (3.53 to 7.79) | 6.37 (4.21 to 9.45) | | 5.98 (3.98 to 8.93) | 6.66 (4.38 to 9.90) | | 6.26 (4.13 to 9.37) | 6.00 (3.97 to 8.92) | | 5.64 (3.76 to 8.42) | 6.00 (3.98 to 8.94) | | 5.64 (3.76 to 8.40) | 6.27 (4.14 to 9.26) | | 5.90 (3.91 to 8.75) |
| Kenya | 12.94 (11.53 to 14.55) | 13.53 (12.04 to 15.23) | 13.08 (11.60 to 14.83) | | 13.68 (12.09 to 15.50) | 12.79 (11.29 to 14.48) | 13.37 (11.80 to 15.11) | 12.71 (11.39 to 14.21) | | 13.30 (11.89 to 14.89) | 13.36 (11.89 to 15.04) | | 13.96 (12.41 to 15.72) | 13.23 (11.40 to 15.38) | | 13.83 (11.90 to 16.10) | 12.76 (11.36 to 14.35) | | 13.34 (11.86 to 15.02) | 13.16 (11.71 to 14.80) | | 13.76 (12.23 to 15.48) | 13.60 (12.10 to 15.34) | | 14.22 (12.64 to 16.03) |
| Iran | 18.25 (17.10 to 19.57) | 17.18 (16.28 to 18.20) | 16.92 (15.67 to 18.33) | | 15.92 (14.92 to 17.06) | 19.57 (18.21 to 21.10) | 18.42 (17.30 to 19.66) | 17.07 (16.00 to 18.33) | | 16.08 (15.21 to 17.06) | 19.28 (18.13 to 20.60) | | 18.15 (17.29 to 19.13) | 20.20 (18.76 to 21.79) | | 19.02 (17.89 to 20.28) | 18.04 (16.83 to 19.44) | | 16.99 (15.99 to 18.09) | 18.05 (16.85 to 19.38) | | 16.99 (16.03 to 18.03) | 18.80 (17.53 to 20.26) | | 17.70 (16.70 to 18.83) |
| Uganda | 3.51 (3.27 to 3.78) | 3.67 (3.41 to 3.95) | 3.45 (3.20 to 3.72) | | 3.61 (3.34 to 3.89) | 3.58 (3.29 to 3.89) | 3.74 (3.44 to 4.07) | 3.45 (3.23 to 3.70) | | 3.61 (3.37 to 3.87) | 3.64 (3.38 to 3.92) | | 3.80 (3.53 to 4.10) | 3.60 (3.20 to 4.04) | | 3.76 (3.34 to 4.23) | 3.48 (3.23 to 3.74) | | 3.63 (3.37 to 3.92) | 3.58 (3.33 to 3.86) | | 3.75 (3.48 to 4.04) | 3.70 (3.43 to 4.01) | | 3.87 (3.58 to 4.19) |
| Japan | 14.83 (12.63 to 17.41) | 14.96 (12.75 to 17.55) | 16.05 (13.40 to 19.21) | | 16.20 (13.52 to 19.35) | 13.49 (11.22 to 16.09) | 13.62 (11.35 to 16.23) | 14.89 (12.68 to 17.48) | | 15.02 (12.81 to 17.64) | 14.94 (12.72 to 17.53) | | 15.07 (12.84 to 17.67) | 14.70 (12.53 to 17.28) | | 14.83 (12.64 to 17.41) | 15.01 (12.77 to 17.66) | | 15.15 (12.88 to 17.82) | 14.83 (12.62 to 17.42) | | 14.96 (12.73 to 17.58) | 14.80 (12.61 to 17.37) | | 14.94 (12.73 to 17.52) |
| South Africa | 3.29 (3.04 to 3.56) | 3.43 (3.17 to 3.73) | 3.28 (3.01 to 3.59) | | 3.43 (3.14 to 3.75) | 3.29 (3.02 to 3.59) | 3.43 (3.15 to 3.75) | 3.22 (3.01 to 3.46) | | 3.37 (3.14 to 3.62) | 3.37 (3.13 to 3.65) | | 3.53 (3.27 to 3.82) | 3.34 (2.95 to 3.77) | | 3.49 (3.09 to 3.95) | 3.21 (2.96 to 3.48) | | 3.35 (3.09 to 3.64) | 3.31 (3.06 to 3.58) | | 3.46 (3.19 to 3.75) | 3.42 (3.14 to 3.71) | | 3.57 (3.28 to 3.88) |
| Sudan | 12.90 (9.13 to 18.63) | 13.49 (9.54 to 19.36) | 12.83 (7.99 to 21.26) | | 13.41 (8.35 to 22.20) | 12.60 (7.95 to 20.55) | 13.18 (8.39 to 21.36) | 12.68 (8.99 to 18.23) | | 13.25 (9.40 to 18.89) | 13.30 (9.44 to 19.19) | | 13.90 (9.84 to 20.10) | 13.15 (9.16 to 19.47) | | 13.80 (9.61 to 20.11) | 12.64 (7.99 to 20.05) | | 13.23 (8.35 to 20.93) | 13.03 (8.24 to 20.69) | | 13.65 (8.64 to 21.76) | 13.49 (8.52 to 21.49) | | 14.09 (8.86 to 22.49) |
| Afghanistan | 13.62 (8.73 to 21.53) | 13.64 (8.74 to 21.61) | 13.43 (7.48 to 24.64) | | 13.41 (7.49 to 24.90) | 13.30 (7.57 to 23.84) | 13.36 (7.62 to 23.92) | 12.95 (8.30 to 20.34) | | 12.95 (8.34 to 20.42) | 14.75 (9.49 to 23.34) | | 14.78 (9.43 to 23.49) | 15.30 (9.61 to 24.91) | | 15.35 (9.59 to 24.85) | 13.48 (8.15 to 22.23) | | 13.49 (8.17 to 22.33) | 14.10 (8.59 to 23.51) | | 14.12 (8.54 to 23.38) | 14.18 (8.58 to 23.42) | | 14.23 (8.60 to 23.79) |
| Myanmar | 7.17 (4.94 to 10.57) | 7.24 (5.02 to 10.69) | 7.22 (4.45 to 12.06) | | 7.29 (4.49 to 12.28) | 6.91 (4.32 to 11.33) | 6.96 (4.38 to 11.46) | 7.17 (4.98 to 10.60) | | 7.24 (5.03 to 10.70) | 7.21 (4.94 to 10.58) | | 7.29 (5.05 to 10.74) | 7.08 (4.84 to 10.45) | | 7.13 (4.88 to 10.53) | 7.11 (4.39 to 11.61) | | 7.20 (4.45 to 11.74) | 7.05 (4.38 to 11.47) | | 7.11 (4.39 to 11.54) | 7.04 (4.33 to 11.45) | | 7.09 (4.38 to 11.50) |

UI=uncertainty interval.

The standardized serving size used for this analysis was a cup (8oz). Total green or black tea intake, including caffeinated, decaffeinated, sweetened or unsweetened tea. This definition excludes herbal tea.

*In previous Global Dietary Database reports, the region central or eastern Europe and central Asia was referred to as the former Soviet Union, and southeast and east Asia was referred to as Asia.

**Supplementary Table 9. Global and regional mean tea intake (cup (8 oz) per week) in adults aged ≥20 years, by age, sex, education, and area of residence across 185 countries in 1990.**

|  | **Mean (95% UI)** | | | | | | | |
| --- | --- | --- | --- | --- | --- | --- | --- | --- |
|  | **Worldwide** | **Central and eastern Europe and central Asia*** | **High income countries** | **Latin America and the Caribbean** | **Middle East and north Africa** | **South Asia*** | **Southeast and east Asia** | **Sub-Saharan Africa** |
| **Overall** | 4.82 (4.51 to 5.21) | 4.13 (3.49 to 4.93) | 4.73 (4.38 to 5.16) | 3.71 (3.40 to 4.09) | 9.48 (8.43 to 10.87) | 4.44 (4.02 to 4.92) | 4.98 (4.27 to 5.90) | 4.08 (3.80 to 4.38) |
| **Sex** |  |  |  |  |  |  |  |  |
| Female | 4.91 (4.54 to 5.37) | 4.14 (3.43 to 5.09) | 4.85 (4.45 to 5.37) | 3.76 (3.42 to 4.17) | 9.24 (8.14 to 10.66) | 4.49 (4.04 to 5.01) | 5.18 (4.33 to 6.37) | 4.11 (3.82 to 4.45) |
| Male | 4.73 (4.38 to 5.17) | 4.11 (3.46 to 4.97) | 4.60 (4.23 to 5.06) | 3.67 (3.33 to 4.06) | 9.73 (8.64 to 11.20) | 4.39 (3.94 to 4.91) | 4.74 (3.95 to 5.85) | 4.04 (3.75 to 4.36) |
| **Age (years)** |  |  |  |  |  |  |  |  |
| 20-39 | 4.56 (4.25 to 4.94) | 4.22 (3.60 to 5.01) | 4.40 (4.08 to 4.78) | 3.70 (3.41 to 4.06) | 8.90 (7.91 to 10.21) | 4.18 (3.87 to 4.53) | 4.55 (3.87 to 5.47) | 4.02 (3.78 to 4.29) |
| 40-59 | 5.11 (4.79 to 5.49) | 4.14 (3.49 to 4.94) | 4.92 (4.54 to 5.36) | 3.81 (3.49 to 4.20) | 10.23 (9.10 to 11.68) | 4.76 (4.28 to 5.29) | 5.44 (4.73 to 6.37) | 4.17 (3.89 to 4.48) |
| ≥60 | 5.20 (4.87 to 5.60) | 3.90 (3.26 to 4.73) | 5.08 (4.68 to 5.57) | 3.55 (3.16 to 4.02) | 10.76 (9.53 to 12.36) | 4.93 (4.11 to 5.92) | 5.73 (4.97 to 6.67) | 4.15 (3.70 to 4.65) |
| **Education (years)** |  |  |  |  |  |  |  |  |
| 0-6 | 4.50 (4.13 to 5.00) | 4.58 (3.77 to 5.64) | 4.28 (3.74 to 5.00) | 3.68 (3.35 to 4.08) | 9.47 (8.34 to 10.99) | 4.38 (3.93 to 4.92) | 3.99 (3.14 to 5.27) | 3.98 (3.70 to 4.31) |
| >6-12 | 4.80 (4.39 to 5.37) | 4.26 (3.62 to 5.09) | 4.78 (4.24 to 5.47) | 3.71 (3.39 to 4.08) | 8.85 (7.73 to 10.31) | 4.44 (4.02 to 4.91) | 4.99 (4.08 to 6.34) | 4.26 (3.97 to 4.58) |
| >12 | 5.27 (4.89 to 5.76) | 3.93 (3.20 to 4.92) | 4.79 (4.52 to 5.14) | 3.80 (3.38 to 4.33) | 10.40 (9.37 to 11.74) | 4.87 (4.29 to 5.62) | 6.36 (5.41 to 7.65) | 4.28 (3.95 to 4.66) |
| **Area of residence** |  |  |  |  |  |  |  |  |
| Rural | 4.47 (4.07 to 4.99) | 4.30 (3.67 to 5.09) | 4.54 (4.20 to 4.95) | 3.73 (3.37 to 4.16) | 9.91 (8.80 to 11.36) | 4.42 (3.99 to 4.91) | 4.14 (3.35 to 5.24) | 4.05 (3.77 to 4.37) |
| Urban | 5.24 (4.93 to 5.60) | 4.02 (3.32 to 4.95) | 4.80 (4.41 to 5.28) | 3.71 (3.39 to 4.08) | 9.15 (8.06 to 10.59) | 4.49 (4.02 to 5.04) | 6.57 (5.74 to 7.62) | 4.13 (3.83 to 4.47) |

UI=uncertainty interval.

The standardized serving size used for this analysis was a cup (8oz). Total green or black tea intake, including caffeinated, decaffeinated, sweetened or unsweetened tea. This definition excludes herbal tea.

*In previous Global Dietary Database reports, the region central or eastern Europe and central Asia was referred to as the former Soviet Union, and southeast and east Asia was referred to as Asia.

**Supplementary Table 10. Global and regional mean tea intake (cup (8 oz) per week) in adults aged ≥20 years, by age, sex, education, and area of residence across 185 countries in 2005.**

|  | **Mean (95% UI)** | | | | | | | |
| --- | --- | --- | --- | --- | --- | --- | --- | --- |
|  | **Worldwide** | **Central and eastern Europe and central Asia*** | **High income countries** | **Latin America and the Caribbean** | **Middle East and north Africa** | **South Asia*** | **Southeast and east Asia** | **Sub-Saharan Africa** |
| **Overall** | 5.45 (5.07 to 5.91) | 6.16 (5.23 to 7.38) | 4.93 (4.56 to 5.37) | 3.83 (3.51 to 4.21) | 8.23 (7.21 to 9.58) | 4.91 (4.45 to 5.42) | 6.06 (5.18 to 7.19) | 4.00 (3.74 to 4.30) |
| **Sex** |  |  |  |  |  |  |  |  |
| Female | 5.52 (5.08 to 6.09) | 6.12 (5.05 to 7.66) | 5.06 (4.64 to 5.59) | 3.87 (3.53 to 4.29) | 7.95 (6.90 to 9.33) | 4.97 (4.50 to 5.50) | 6.25 (5.20 to 7.72) | 4.04 (3.76 to 4.36) |
| Male | 5.35 (4.93 to 5.89) | 6.20 (5.22 to 7.57) | 4.78 (4.40 to 5.26) | 3.78 (3.44 to 4.18) | 8.51 (7.46 to 9.92) | 4.86 (4.38 to 5.41) | 5.81 (4.83 to 7.16) | 3.97 (3.69 to 4.29) |
| **Age (years)** |  |  |  |  |  |  |  |  |
| 20-39 | 5.15 (4.79 to 5.58) | 6.32 (5.41 to 7.47) | 4.55 (4.21 to 4.96) | 3.82 (3.52 to 4.18) | 7.81 (6.86 to 9.11) | 4.60 (4.28 to 4.96) | 5.64 (4.80 to 6.73) | 3.93 (3.70 to 4.19) |
| 40-59 | 5.61 (5.21 to 6.10) | 6.23 (5.26 to 7.49) | 5.10 (4.73 to 5.54) | 3.93 (3.60 to 4.31) | 8.73 (7.66 to 10.15) | 5.26 (4.75 to 5.81) | 6.05 (5.16 to 7.21) | 4.14 (3.87 to 4.45) |
| ≥60 | 6.03 (5.61 to 6.53) | 5.78 (4.84 to 7.04) | 5.22 (4.80 to 5.71) | 3.65 (3.24 to 4.12) | 9.00 (7.83 to 10.52) | 5.44 (4.53 to 6.53) | 7.32 (6.36 to 8.52) | 4.12 (3.67 to 4.62) |
| **Education (years)** |  |  |  |  |  |  |  |  |
| 0-6 | 4.90 (4.51 to 5.38) | 6.30 (5.19 to 7.83) | 4.47 (3.95 to 5.14) | 3.80 (3.46 to 4.23) | 7.83 (6.68 to 9.44) | 4.77 (4.29 to 5.34) | 5.02 (4.04 to 6.44) | 3.91 (3.63 to 4.24) |
| >6-12 | 5.28 (4.74 to 6.03) | 6.32 (5.44 to 7.48) | 4.90 (4.35 to 5.61) | 3.83 (3.51 to 4.21) | 7.80 (6.69 to 9.29) | 4.97 (4.49 to 5.52) | 5.54 (4.43 to 7.16) | 4.06 (3.79 to 4.36) |
| >12 | 5.93 (5.51 to 6.48) | 6.10 (5.07 to 7.52) | 4.97 (4.63 to 5.39) | 3.84 (3.46 to 4.32) | 9.16 (8.23 to 10.37) | 5.03 (4.53 to 5.60) | 7.24 (6.13 to 8.75) | 4.28 (3.97 to 4.62) |
| **Area of residence** |  |  |  |  |  |  |  |  |
| Rural | 5.07 (4.60 to 5.66) | 6.35 (5.45 to 7.56) | 4.79 (4.41 to 5.25) | 3.84 (3.45 to 4.30) | 8.07 (7.04 to 9.45) | 4.90 (4.44 to 5.42) | 5.11 (4.09 to 6.53) | 3.92 (3.66 to 4.23) |
| Urban | 5.79 (5.39 to 6.29) | 6.05 (4.97 to 7.55) | 4.97 (4.57 to 5.45) | 3.83 (3.51 to 4.20) | 8.32 (7.28 to 9.71) | 4.93 (4.44 to 5.49) | 7.08 (6.06 to 8.44) | 4.16 (3.86 to 4.50) |

UI=uncertainty interval.

The standardized serving size used for this analysis was a cup (8oz). Total green or black tea intake, including caffeinated, decaffeinated, sweetened or unsweetened tea. This definition excludes herbal tea.

*In previous Global Dietary Database reports, the region central or eastern Europe and central Asia was referred to as the former Soviet Union, and southeast and east Asia was referred to as Asia.

**Supplementary Table 11. National mean tea intake (cup (8 oz) per week) in adults aged ≥20 years by sex, age, parental education, and area of residence in the 25 most populous countries in 1990.**

| **Country** | **Mean (95% UI)** | | | | | | | | | | |
| --- | --- | --- | --- | --- | --- | --- | --- | --- | --- | --- | --- |
|  | **Overall** | **Sex** | | **Age** | | | **Education** | | | **Area of residence** | |
|  |  | **Female** | **Male** | **20-39 years** | **40-59 years** | **≥60 years** | **0-6 years** | **>6-12 years** | **>12 years** | **Rural** | **Urban** |
| India | 4.28 (3.87 to 4.74) | 4.36 (3.94 to 4.83) | 4.21 (3.78 to 4.69) | 4.03 (3.73 to 4.36) | 4.59 (4.13 to 5.11) | 4.74 (3.95 to 5.69) | 4.20 (3.79 to 4.65) | 4.39 (3.96 to 4.87) | 4.41 (3.97 to 4.91) | 4.28 (3.87 to 4.74) | 4.29 (3.87 to 4.77) |
| China | 3.79 (2.87 to 5.03) | 3.85 (2.72 to 5.53) | 3.68 (2.63 to 5.22) | 3.79 (2.87 to 5.03) | 3.81 (2.89 to 5.04) | 3.73 (2.81 to 4.97) | 3.78 (2.55 to 5.66) | 3.73 (2.54 to 5.58) | 3.73 (2.53 to 5.57) | 3.77 (2.73 to 5.23) | 3.79 (2.75 to 5.29) |
| Nigeria | 3.50 (3.26 to 3.77) | 3.40 (3.16 to 3.66) | 3.60 (3.32 to 3.91) | 3.43 (3.22 to 3.67) | 3.61 (3.36 to 3.87) | 3.57 (3.19 to 3.99) | 3.46 (3.22 to 3.73) | 3.57 (3.32 to 3.85) | 3.69 (3.42 to 3.99) | 3.45 (3.21 to 3.72) | 3.61 (3.35 to 3.89) |
| Pakistan | 6.08 (4.35 to 8.59) | 6.08 (4.05 to 9.41) | 5.98 (4.04 to 9.10) | 5.72 (4.14 to 8.04) | 6.54 (4.66 to 9.22) | 6.76 (4.66 to 9.84) | 5.95 (3.94 to 9.08) | 6.22 (4.14 to 9.45) | 6.24 (4.15 to 9.48) | 6.04 (4.10 to 9.05) | 6.06 (4.11 to 9.06) |
| Indonesia | 3.81 (3.59 to 4.06) | 3.82 (3.56 to 4.11) | 3.79 (3.54 to 4.07) | 3.81 (3.59 to 4.06) | 3.83 (3.60 to 4.08) | 3.75 (3.53 to 4.00) | 3.83 (3.61 to 4.08) | 3.78 (3.56 to 4.04) | 3.77 (3.55 to 4.03) | 3.80 (3.57 to 4.05) | 3.83 (3.61 to 4.08) |
| United States | 4.68 (4.50 to 4.86) | 4.70 (4.53 to 4.89) | 4.64 (4.46 to 4.83) | 4.36 (4.20 to 4.53) | 4.89 (4.71 to 5.08) | 5.03 (4.84 to 5.24) | 4.30 (4.13 to 4.47) | 4.70 (4.52 to 4.88) | 4.69 (4.51 to 4.88) | 4.61 (4.49 to 4.74) | 4.70 (4.50 to 4.91) |
| Brazil | 3.41 (3.15 to 3.68) | 3.42 (3.11 to 3.76) | 3.39 (3.10 to 3.71) | 3.40 (3.16 to 3.67) | 3.49 (3.24 to 3.76) | 3.23 (2.93 to 3.56) | 3.41 (3.15 to 3.70) | 3.41 (3.15 to 3.69) | 3.39 (3.12 to 3.69) | 3.41 (3.13 to 3.71) | 3.40 (3.15 to 3.68) |
| Bangladesh | 3.36 (3.09 to 3.66) | 3.15 (2.89 to 3.43) | 3.54 (3.19 to 3.94) | 3.16 (2.94 to 3.40) | 3.60 (3.33 to 3.90) | 3.73 (3.19 to 4.35) | 3.29 (3.02 to 3.59) | 3.44 (3.15 to 3.76) | 3.46 (3.16 to 3.79) | 3.35 (3.08 to 3.65) | 3.36 (3.07 to 3.70) |
| Ethiopia | 4.19 (3.95 to 4.45) | 4.67 (4.39 to 4.98) | 3.69 (3.44 to 3.97) | 4.11 (3.91 to 4.33) | 4.32 (4.07 to 4.59) | 4.28 (3.84 to 4.75) | 4.14 (3.90 to 4.40) | 4.27 (4.02 to 4.54) | 4.41 (4.13 to 4.72) | 4.16 (3.93 to 4.43) | 4.35 (4.10 to 4.63) |
| Democratic Republic of the Congo | 4.00 (3.04 to 5.37) | 3.98 (2.77 to 5.99) | 3.92 (2.77 to 5.77) | 3.92 (2.99 to 5.26) | 4.12 (3.14 to 5.52) | 4.09 (3.05 to 5.59) | 3.93 (2.76 to 5.67) | 4.06 (2.86 to 5.85) | 4.19 (2.96 to 6.05) | 3.91 (2.76 to 5.70) | 4.10 (2.91 to 5.93) |
| Mexico | 3.53 (3.18 to 3.91) | 3.55 (3.14 to 4.01) | 3.50 (3.10 to 3.94) | 3.52 (3.20 to 3.88) | 3.62 (3.26 to 4.01) | 3.33 (2.91 to 3.82) | 3.53 (3.18 to 3.92) | 3.53 (3.18 to 3.92) | 3.51 (3.16 to 3.90) | 3.53 (3.18 to 3.92) | 3.52 (3.18 to 3.91) |
| Philippines | 4.11 (3.85 to 4.39) | 4.28 (4.01 to 4.58) | 3.93 (3.65 to 4.24) | 4.10 (3.85 to 4.39) | 4.13 (3.87 to 4.41) | 4.06 (3.80 to 4.35) | 4.15 (3.89 to 4.44) | 4.10 (3.84 to 4.38) | 4.09 (3.83 to 4.37) | 4.09 (3.83 to 4.37) | 4.13 (3.87 to 4.41) |
| Egypt | 11.57 (10.42 to 12.82) | 11.62 (10.51 to 12.83) | 11.53 (10.28 to 12.88) | 10.88 (9.84 to 11.99) | 12.43 (11.18 to 13.81) | 12.99 (11.55 to 14.63) | 11.49 (10.34 to 12.75) | 11.49 (10.31 to 12.77) | 11.97 (10.74 to 13.35) | 11.88 (10.67 to 13.19) | 11.18 (10.05 to 12.41) |
| Russia | 3.60 (2.62 to 4.98) | 3.64 (2.46 to 5.48) | 3.51 (2.40 to 5.17) | 3.66 (2.68 to 5.03) | 3.63 (2.63 to 5.00) | 3.47 (2.49 to 4.86) | 4.02 (2.66 to 6.11) | 3.58 (2.38 to 5.41) | 3.48 (2.31 to 5.28) | 3.67 (2.53 to 5.35) | 3.56 (2.46 to 5.19) |
| Tanzania | 3.80 (3.52 to 4.11) | 3.89 (3.59 to 4.21) | 3.70 (3.38 to 4.06) | 3.72 (3.47 to 4.00) | 3.91 (3.63 to 4.23) | 3.88 (3.46 to 4.38) | 3.75 (3.47 to 4.06) | 3.86 (3.58 to 4.18) | 3.99 (3.67 to 4.34) | 3.76 (3.49 to 4.07) | 3.93 (3.64 to 4.26) |
| Vietnam | 3.36 (3.14 to 3.61) | 3.37 (3.11 to 3.66) | 3.36 (3.10 to 3.64) | 3.37 (3.14 to 3.62) | 3.38 (3.16 to 3.64) | 3.31 (3.08 to 3.56) | 3.39 (3.16 to 3.64) | 3.35 (3.12 to 3.59) | 3.34 (3.11 to 3.59) | 3.36 (3.13 to 3.61) | 3.39 (3.16 to 3.64) |
| Turkey | 5.46 (3.64 to 8.10) | 5.36 (3.49 to 8.10) | 5.56 (3.66 to 8.28) | 5.10 (3.42 to 7.55) | 5.86 (3.91 to 8.71) | 6.12 (4.04 to 9.12) | 5.41 (3.61 to 8.04) | 5.41 (3.61 to 8.03) | 5.66 (3.75 to 8.35) | 5.66 (3.76 to 8.40) | 5.32 (3.55 to 7.91) |
| Kenya | 8.37 (7.60 to 9.23) | 8.45 (7.62 to 9.39) | 8.28 (7.46 to 9.19) | 8.23 (7.52 to 9.03) | 8.69 (7.87 to 9.58) | 8.60 (7.55 to 9.80) | 8.25 (7.49 to 9.10) | 8.51 (7.73 to 9.38) | 8.80 (7.96 to 9.72) | 8.31 (7.55 to 9.15) | 8.68 (7.88 to 9.58) |
| Iran | 16.26 (15.35 to 17.21) | 15.06 (14.07 to 16.12) | 17.43 (16.32 to 18.58) | 15.32 (14.43 to 16.24) | 17.52 (16.64 to 18.47) | 18.34 (17.26 to 19.47) | 16.08 (15.09 to 17.13) | 16.07 (15.14 to 17.05) | 16.76 (15.75 to 17.83) | 16.82 (15.73 to 17.99) | 15.83 (14.97 to 16.74) |
| Uganda | 3.54 (3.28 to 3.81) | 3.47 (3.22 to 3.75) | 3.60 (3.31 to 3.92) | 3.48 (3.25 to 3.72) | 3.66 (3.40 to 3.94) | 3.62 (3.22 to 4.07) | 3.50 (3.25 to 3.78) | 3.61 (3.35 to 3.89) | 3.73 (3.44 to 4.04) | 3.52 (3.26 to 3.79) | 3.68 (3.41 to 3.97) |
| Japan | 18.54 (15.81 to 21.77) | 20.09 (16.78 to 24.03) | 16.89 (14.09 to 20.15) | 18.53 (15.81 to 21.78) | 18.63 (15.88 to 21.83) | 18.43 (15.70 to 21.66) | 18.78 (15.97 to 22.10) | 18.55 (15.77 to 21.79) | 18.52 (15.80 to 21.73) | 18.42 (15.70 to 21.64) | 18.58 (15.84 to 21.82) |
| South Africa | 4.12 (3.83 to 4.45) | 4.12 (3.79 to 4.50) | 4.12 (3.80 to 4.48) | 4.05 (3.79 to 4.33) | 4.25 (3.95 to 4.58) | 4.20 (3.73 to 4.73) | 4.02 (3.73 to 4.35) | 4.15 (3.85 to 4.48) | 4.29 (3.96 to 4.64) | 4.03 (3.74 to 4.35) | 4.21 (3.91 to 4.55) |
| Sudan | 4.87 (3.71 to 6.53) | 4.87 (3.38 to 7.24) | 4.78 (3.37 to 7.01) | 4.78 (3.65 to 6.41) | 5.04 (3.82 to 6.74) | 4.98 (3.75 to 6.75) | 4.78 (3.37 to 6.92) | 4.93 (3.48 to 7.18) | 5.10 (3.58 to 7.36) | 4.78 (3.40 to 6.89) | 4.99 (3.54 to 7.14) |
| Afghanistan | 9.23 (6.27 to 13.82) | 9.12 (5.61 to 15.50) | 9.04 (5.69 to 14.99) | 8.72 (5.97 to 13.03) | 9.98 (6.75 to 14.93) | 10.30 (6.81 to 15.71) | 9.13 (5.96 to 14.19) | 9.57 (6.28 to 14.81) | 9.60 (6.33 to 14.89) | 9.16 (5.87 to 14.53) | 9.20 (5.90 to 14.47) |
| Myanmar | 3.96 (2.91 to 5.49) | 4.00 (2.69 to 6.13) | 3.82 (2.61 to 5.78) | 3.96 (2.91 to 5.51) | 3.97 (2.92 to 5.49) | 3.90 (2.85 to 5.42) | 3.95 (2.67 to 5.91) | 3.90 (2.64 to 5.84) | 3.89 (2.64 to 5.80) | 3.93 (2.73 to 5.77) | 3.95 (2.74 to 5.81) |

UI=uncertainty interval.

The standardized serving size used for this analysis was a cup (8oz). Total green or black tea intake, including caffeinated, decaffeinated, sweetened or unsweetened tea. This definition excludes herbal tea.

**Supplementary Table 12. National mean tea intake (cup (8 oz) per week) in adults aged ≥20 years by sex, age, parental education, and area of residence in the 25 most populous countries in 2005.**

| **Country** | **Mean (95% UI)** | | | | | | | | | | |
| --- | --- | --- | --- | --- | --- | --- | --- | --- | --- | --- | --- |
|  | **Overall** | **Sex** | | **Age** | | | **Education** | | | **Area of residence** | |
|  |  | **Female** | **Male** | **20-39 years** | **40-59 years** | **≥60 years** | **0-6 years** | **>6-12 years** | **>12 years** | **Rural** | **Urban** |
| India | 4.90 (4.43 to 5.42) | 4.99 (4.51 to 5.52) | 4.81 (4.32 to 5.37) | 4.59 (4.25 to 4.96) | 5.23 (4.71 to 5.80) | 5.41 (4.50 to 6.51) | 4.76 (4.30 to 5.27) | 4.98 (4.48 to 5.53) | 5.00 (4.50 to 5.57) | 4.89 (4.42 to 5.42) | 4.91 (4.43 to 5.45) |
| China | 4.90 (3.74 to 6.47) | 5.00 (3.57 to 7.08) | 4.76 (3.43 to 6.66) | 4.91 (3.75 to 6.48) | 4.92 (3.76 to 6.51) | 4.81 (3.65 to 6.37) | 4.90 (3.40 to 7.15) | 4.86 (3.37 to 7.05) | 4.85 (3.36 to 7.02) | 4.86 (3.49 to 6.79) | 4.90 (3.52 to 6.89) |
| Nigeria | 3.52 (3.28 to 3.79) | 3.42 (3.18 to 3.68) | 3.63 (3.35 to 3.93) | 3.46 (3.24 to 3.69) | 3.64 (3.39 to 3.91) | 3.60 (3.22 to 4.03) | 3.47 (3.23 to 3.74) | 3.58 (3.33 to 3.86) | 3.70 (3.43 to 3.99) | 3.46 (3.22 to 3.72) | 3.62 (3.37 to 3.90) |
| Pakistan | 5.63 (4.15 to 7.68) | 5.64 (3.89 to 8.25) | 5.55 (3.90 to 8.02) | 5.30 (3.94 to 7.17) | 6.05 (4.47 to 8.24) | 6.26 (4.45 to 8.84) | 5.44 (3.65 to 8.19) | 5.68 (3.80 to 8.58) | 5.72 (3.81 to 8.58) | 5.60 (3.94 to 8.01) | 5.62 (3.93 to 8.08) |
| Indonesia | 4.04 (3.80 to 4.31) | 4.05 (3.78 to 4.37) | 4.03 (3.75 to 4.32) | 4.05 (3.80 to 4.32) | 4.06 (3.82 to 4.33) | 3.97 (3.73 to 4.25) | 4.08 (3.83 to 4.35) | 4.03 (3.78 to 4.30) | 4.02 (3.77 to 4.29) | 4.02 (3.78 to 4.30) | 4.06 (3.82 to 4.33) |
| United States | 4.92 (4.75 to 5.10) | 4.95 (4.77 to 5.14) | 4.88 (4.70 to 5.07) | 4.54 (4.38 to 4.70) | 5.10 (4.92 to 5.29) | 5.24 (5.05 to 5.45) | 4.51 (4.33 to 4.69) | 4.93 (4.75 to 5.11) | 4.92 (4.75 to 5.11) | 4.84 (4.73 to 4.96) | 4.93 (4.74 to 5.15) |
| Brazil | 3.65 (3.40 to 3.93) | 3.67 (3.36 to 4.02) | 3.63 (3.33 to 3.95) | 3.65 (3.41 to 3.91) | 3.74 (3.50 to 4.01) | 3.45 (3.14 to 3.81) | 3.66 (3.40 to 3.94) | 3.66 (3.40 to 3.95) | 3.64 (3.37 to 3.94) | 3.66 (3.37 to 3.96) | 3.65 (3.40 to 3.93) |
| Bangladesh | 3.97 (3.66 to 4.32) | 3.73 (3.43 to 4.06) | 4.20 (3.79 to 4.68) | 3.74 (3.49 to 4.01) | 4.28 (3.96 to 4.63) | 4.41 (3.78 to 5.15) | 3.86 (3.55 to 4.21) | 4.04 (3.71 to 4.41) | 4.06 (3.72 to 4.43) | 3.97 (3.66 to 4.31) | 3.98 (3.64 to 4.37) |
| Ethiopia | 4.22 (3.98 to 4.48) | 4.70 (4.42 to 5.01) | 3.72 (3.46 to 4.00) | 4.14 (3.93 to 4.36) | 4.36 (4.12 to 4.64) | 4.32 (3.88 to 4.80) | 4.15 (3.91 to 4.41) | 4.28 (4.03 to 4.55) | 4.42 (4.14 to 4.73) | 4.19 (3.95 to 4.45) | 4.38 (4.12 to 4.66) |
| Democratic Republic of the Congo | 4.02 (3.10 to 5.33) | 4.02 (2.86 to 5.87) | 3.96 (2.85 to 5.69) | 3.94 (3.06 to 5.20) | 4.15 (3.19 to 5.50) | 4.12 (3.11 to 5.53) | 3.95 (2.81 to 5.64) | 4.08 (2.90 to 5.77) | 4.21 (2.99 to 6.01) | 3.93 (2.81 to 5.66) | 4.10 (2.94 to 5.89) |
| Mexico | 3.53 (3.18 to 3.92) | 3.55 (3.15 to 4.02) | 3.50 (3.10 to 3.95) | 3.53 (3.21 to 3.89) | 3.62 (3.26 to 4.01) | 3.32 (2.90 to 3.81) | 3.53 (3.18 to 3.94) | 3.54 (3.18 to 3.93) | 3.52 (3.16 to 3.91) | 3.53 (3.18 to 3.93) | 3.53 (3.18 to 3.92) |
| Philippines | 4.10 (3.85 to 4.39) | 4.27 (4.00 to 4.58) | 3.93 (3.65 to 4.23) | 4.10 (3.85 to 4.39) | 4.12 (3.87 to 4.41) | 4.05 (3.80 to 4.34) | 4.15 (3.89 to 4.44) | 4.10 (3.84 to 4.39) | 4.09 (3.83 to 4.38) | 4.09 (3.83 to 4.37) | 4.12 (3.87 to 4.41) |
| Egypt | 4.68 (4.32 to 5.07) | 4.70 (4.35 to 5.07) | 4.65 (4.25 to 5.09) | 4.38 (4.07 to 4.72) | 5.01 (4.62 to 5.45) | 5.25 (4.77 to 5.79) | 4.61 (4.25 to 5.01) | 4.61 (4.24 to 5.01) | 4.81 (4.41 to 5.23) | 4.80 (4.42 to 5.21) | 4.52 (4.16 to 4.90) |
| Russia | 5.63 (3.95 to 8.11) | 5.67 (3.63 to 9.09) | 5.46 (3.55 to 8.57) | 5.73 (4.03 to 8.17) | 5.68 (4.00 to 8.18) | 5.39 (3.73 to 7.87) | 6.41 (4.23 to 9.72) | 5.71 (3.80 to 8.63) | 5.55 (3.68 to 8.41) | 5.72 (3.75 to 8.81) | 5.56 (3.64 to 8.52) |
| Tanzania | 3.82 (3.53 to 4.13) | 3.90 (3.61 to 4.23) | 3.72 (3.40 to 4.08) | 3.75 (3.49 to 4.02) | 3.94 (3.66 to 4.27) | 3.92 (3.49 to 4.42) | 3.75 (3.47 to 4.06) | 3.87 (3.58 to 4.18) | 3.99 (3.68 to 4.34) | 3.77 (3.50 to 4.08) | 3.94 (3.65 to 4.27) |
| Vietnam | 5.68 (5.26 to 6.12) | 5.69 (5.24 to 6.19) | 5.67 (5.21 to 6.16) | 5.68 (5.27 to 6.12) | 5.71 (5.29 to 6.16) | 5.57 (5.17 to 6.02) | 5.73 (5.31 to 6.18) | 5.66 (5.25 to 6.10) | 5.65 (5.23 to 6.09) | 5.66 (5.25 to 6.10) | 5.71 (5.30 to 6.16) |
| Turkey | 5.76 (3.84 to 8.55) | 5.66 (3.68 to 8.54) | 5.87 (3.87 to 8.77) | 5.37 (3.59 to 7.94) | 6.12 (4.07 to 9.08) | 6.41 (4.23 to 9.57) | 5.70 (3.79 to 8.50) | 5.69 (3.79 to 8.47) | 5.95 (3.95 to 8.80) | 6.01 (3.98 to 8.92) | 5.65 (3.77 to 8.39) |
| Kenya | 3.78 (3.50 to 4.10) | 3.83 (3.50 to 4.18) | 3.74 (3.43 to 4.08) | 3.73 (3.46 to 4.01) | 3.92 (3.62 to 4.25) | 3.89 (3.44 to 4.38) | 3.70 (3.42 to 4.01) | 3.82 (3.53 to 4.14) | 3.95 (3.63 to 4.28) | 3.75 (3.47 to 4.06) | 3.92 (3.62 to 4.25) |
| Iran | 16.01 (15.10 to 16.94) | 14.83 (13.84 to 15.88) | 17.15 (16.06 to 18.28) | 15.01 (14.13 to 15.91) | 17.25 (16.37 to 18.15) | 18.08 (16.96 to 19.20) | 15.72 (14.73 to 16.75) | 15.71 (14.78 to 16.68) | 16.38 (15.38 to 17.43) | 16.67 (15.57 to 17.85) | 15.69 (14.80 to 16.60) |
| Uganda | 3.56 (3.30 to 3.83) | 3.49 (3.24 to 3.77) | 3.62 (3.33 to 3.94) | 3.50 (3.27 to 3.75) | 3.68 (3.42 to 3.96) | 3.63 (3.22 to 4.09) | 3.51 (3.26 to 3.78) | 3.62 (3.36 to 3.90) | 3.74 (3.46 to 4.04) | 3.53 (3.28 to 3.80) | 3.69 (3.43 to 3.97) |
| Japan | 22.43 (19.15 to 26.38) | 24.31 (20.28 to 29.06) | 20.42 (17.03 to 24.36) | 22.49 (19.21 to 26.46) | 22.53 (19.25 to 26.47) | 22.27 (18.98 to 26.20) | 22.73 (19.35 to 26.76) | 22.46 (19.11 to 26.40) | 22.42 (19.13 to 26.35) | 22.26 (18.97 to 26.18) | 22.46 (19.17 to 26.40) |
| South Africa | 4.50 (4.17 to 4.87) | 4.49 (4.12 to 4.91) | 4.51 (4.14 to 4.90) | 4.41 (4.12 to 4.73) | 4.64 (4.31 to 5.01) | 4.59 (4.06 to 5.18) | 4.34 (4.02 to 4.70) | 4.48 (4.15 to 4.85) | 4.63 (4.27 to 5.01) | 4.38 (4.06 to 4.74) | 4.58 (4.24 to 4.96) |
| Sudan | 4.12 (3.20 to 5.39) | 4.14 (2.93 to 5.96) | 4.05 (2.91 to 5.74) | 4.05 (3.15 to 5.29) | 4.25 (3.30 to 5.56) | 4.21 (3.21 to 5.59) | 4.03 (2.84 to 5.79) | 4.16 (2.95 to 5.99) | 4.29 (3.04 to 6.15) | 4.04 (2.93 to 5.66) | 4.22 (3.05 to 5.92) |
| Afghanistan | 5.17 (3.66 to 7.39) | 5.14 (3.35 to 8.17) | 5.09 (3.38 to 7.88) | 4.90 (3.49 to 6.97) | 5.60 (3.95 to 8.04) | 5.76 (3.99 to 8.49) | 5.06 (3.32 to 7.77) | 5.31 (3.48 to 8.20) | 5.32 (3.50 to 8.25) | 5.14 (3.47 to 7.71) | 5.16 (3.48 to 7.76) |
| Myanmar | 4.93 (3.70 to 6.62) | 5.00 (3.50 to 7.30) | 4.76 (3.36 to 6.85) | 4.93 (3.70 to 6.61) | 4.95 (3.72 to 6.67) | 4.86 (3.64 to 6.54) | 4.90 (3.34 to 7.28) | 4.86 (3.30 to 7.23) | 4.83 (3.31 to 7.21) | 4.89 (3.49 to 6.92) | 4.94 (3.54 to 7.00) |

UI=uncertainty interval.

The standardized serving size used for this analysis was a cup (8oz). Total green or black tea intake, including caffeinated, decaffeinated, sweetened or unsweetened tea. This definition excludes herbal tea.

**Supplementary Table 13. Global and regional EAPC in mean tea intake (cup (8 oz) per week) from 1990-2005, 2005-2018, and 1990-2018 in adults aged ≥20 years, by sex, age, education, and area of residence across 185 countries.**

|  |  | **EAPC (95% CI)** | | | | | | | |
| --- | --- | --- | --- | --- | --- | --- | --- | --- | --- |
|  |  | **Worldwide** | **Central and eastern Europe and central Asia*** | **High income countries** | **Latin America and the Caribbean** | **Middle East and north Africa** | **South Asia*** | **Southeast and east Asia** | **Sub-Saharan Africa** |
| **Sex** |  |  |  |  |  |  |  |  |  |
| Female | 1990-2005 | 0.72 (0.44 to 1.00) | 2.43 (0.49 to 4.40) | 0.29 (0.15 to 0.43) | 0.20 (-0.04 to 0.45) | -0.93 (-1.21 to -0.65) | 0.57 (0.13 to 1.02) | 1.17 (0.60 to 1.74) | -0.18 (-0.56 to 0.21) |
|  | 2005-2018 | 1.00 (0.69 to 1.31) | -1.21 (-2.78 to 0.38) | -0.09 (-0.30 to 0.13) | 0.07 (0.04 to 0.09) | 0.66 (-0.73 to 2.06) | 1.09 (-0.02 to 2.22) | 1.76 (1.36 to 2.15) | 1.90 (0.93 to 2.88) |
|  | 1990-2018 | 0.91 (0.78 to 1.04) | 0.68 (-0.42 to 1.79) | 0.10 (0.01 to 0.19) | 0.09 (0.01 to 0.18) | -0.00 (-0.46 to 0.45) | 0.75 (0.42 to 1.08) | 1.61 (1.35 to 1.87) | 0.78 (0.22 to 1.33) |
| Male | 1990-2005 | 0.75 (0.44 to 1.06) | 2.53 (0.23 to 4.88) | 0.27 (0.13 to 0.40) | 0.21 (-0.01 to 0.43) | -0.82 (-1.11 to -0.53) | 0.58 (0.15 to 1.01) | 1.27 (0.68 to 1.87) | -0.18 (-0.57 to 0.21) |
|  | 2005-2018 | 1.07 (0.74 to 1.40) | -1.49 (-3.55 to 0.62) | -0.08 (-0.29 to 0.14) | 0.05 (0.01 to 0.09) | 0.52 (-0.68 to 1.74) | 1.13 (-0.01 to 2.28) | 1.95 (1.49 to 2.40) | 1.88 (0.93 to 2.83) |
|  | 1990-2018 | 0.96 (0.82 to 1.11) | 0.62 (-0.66 to 1.92) | 0.09 (0.01 to 0.18) | 0.09 (0.01 to 0.17) | -0.03 (-0.42 to 0.37) | 0.76 (0.42 to 1.10) | 1.76 (1.48 to 2.05) | 0.77 (0.22 to 1.32) |
| **Age (years)** |  |  |  |  |  |  |  |  |  |
| 20-39 | 1990-2005 | 0.74 (0.43 to 1.05) | 2.50 (0.41 to 4.63) | 0.23 (0.12 to 0.35) | 0.22 (-0.01 to 0.46) | -0.80 (-1.07 to -0.53) | 0.54 (0.12 to 0.96) | 1.33 (0.68 to 1.99) | -0.22 (-0.64 to 0.19) |
|  | 2005-2018 | 1.08 (0.69 to 1.47) | -1.22 (-2.92 to 0.52) | -0.14 (-0.31 to 0.03) | 0.09 (0.05 to 0.13) | 0.46 (-0.82 to 1.75) | 1.15 (0.03 to 2.28) | 2.11 (1.56 to 2.65) | 1.91 (0.94 to 2.89) |
|  | 1990-2018 | 0.97 (0.82 to 1.13) | 0.71 (-0.45 to 1.87) | 0.05 (-0.04 to 0.14) | 0.11 (0.03 to 0.19) | -0.03 (-0.43 to 0.38) | 0.75 (0.41 to 1.09) | 1.90 (1.58 to 2.22) | 0.76 (0.19 to 1.33) |
| 40-59 | 1990-2005 | 0.55 (0.26 to 0.85) | 2.54 (0.38 to 4.74) | 0.25 (0.12 to 0.38) | 0.22 (-0.01 to 0.44) | -0.99 (-1.28 to -0.69) | 0.56 (0.13 to 1.00) | 0.62 (0.07 to 1.17) | -0.11 (-0.48 to 0.26) |
|  | 2005-2018 | 1.09 (0.80 to 1.38) | -1.39 (-3.17 to 0.42) | -0.13 (-0.34 to 0.07) | 0.03 (-0.01 to 0.07) | 0.60 (-0.75 to 1.97) | 1.03 (-0.09 to 2.16) | 1.89 (1.53 to 2.25) | 1.87 (0.94 to 2.82) |
|  | 1990-2018 | 0.85 (0.69 to 1.02) | 0.64 (-0.57 to 1.87) | 0.06 (-0.03 to 0.15) | 0.08 (-0.00 to 0.17) | -0.07 (-0.52 to 0.38) | 0.71 (0.39 to 1.04) | 1.36 (1.00 to 1.72) | 0.80 (0.27 to 1.33) |
| ≥60 | 1990-2005 | 0.92 (0.64 to 1.21) | 2.42 (0.31 to 4.58) | 0.18 (0.01 to 0.35) | 0.19 (-0.02 to 0.40) | -1.12 (-1.44 to -0.81) | 0.55 (0.12 to 0.99) | 1.58 (1.11 to 2.05) | -0.11 (-0.51 to 0.28) |
|  | 2005-2018 | 0.58 (0.35 to 0.81) | -1.40 (-3.40 to 0.65) | -0.09 (-0.35 to 0.17) | 0.03 (-0.01 to 0.08) | 0.76 (-0.61 to 2.14) | 0.99 (-0.12 to 2.12) | 0.81 (0.53 to 1.09) | 1.76 (0.88 to 2.64) |
|  | 1990-2018 | 0.81 (0.68 to 0.94) | 0.64 (-0.57 to 1.86) | 0.05 (-0.04 to 0.13) | 0.08 (0.00 to 0.15) | -0.08 (-0.57 to 0.41) | 0.70 (0.37 to 1.02) | 1.35 (1.12 to 1.57) | 0.76 (0.26 to 1.27) |
| **Education (years)** |  |  |  |  |  |  |  |  |  |
| 0-6 | 1990-2005 | 0.49 (0.16 to 0.83) | 2.03 (0.45 to 3.63) | 0.28 (0.17 to 0.40) | 0.23 (-0.06 to 0.52) | -1.21 (-1.43 to -0.98) | 0.48 (0.09 to 0.86) | 1.40 (0.56 to 2.25) | -0.17 (-0.45 to 0.11) |
|  | 2005-2018 | 1.81 (1.21 to 2.41) | -0.72 (-1.63 to 0.20) | 0.21 (-0.42 to 0.86) | 0.03 (-0.03 to 0.10) | 0.88 (-0.48 to 2.25) | 1.33 (0.11 to 2.56) | 2.88 (2.18 to 3.58) | 1.96 (1.05 to 2.88) |
|  | 1990-2018 | 1.14 (0.79 to 1.49) | 0.63 (-0.21 to 1.47) | 0.17 (0.01 to 0.33) | 0.09 (-0.01 to 0.19) | -0.05 (-0.58 to 0.48) | 0.77 (0.38 to 1.16) | 2.35 (1.87 to 2.84) | 0.80 (0.25 to 1.35) |
| >6-12 | 1990-2005 | 0.56 (0.21 to 0.91) | 2.43 (0.16 to 4.74) | 0.18 (0.04 to 0.33) | 0.23 (0.04 to 0.42) | -0.73 (-1.18 to -0.28) | 0.64 (0.18 to 1.11) | 0.58 (-0.17 to 1.34) | -0.40 (-0.96 to 0.16) |
|  | 2005-2018 | 1.35 (0.90 to 1.80) | -1.51 (-3.52 to 0.54) | 0.00 (-0.38 to 0.38) | 0.07 (0.03 to 0.12) | 0.64 (-0.36 to 1.65) | 1.08 (-0.01 to 2.18) | 2.70 (2.06 to 3.35) | 2.21 (1.07 to 3.37) |
|  | 1990-2018 | 1.03 (0.79 to 1.27) | 0.53 (-0.72 to 1.80) | 0.06 (-0.05 to 0.16) | 0.11 (0.04 to 0.19) | 0.04 (-0.34 to 0.43) | 0.80 (0.48 to 1.12) | 1.78 (1.21 to 2.36) | 0.81 (0.12 to 1.51) |
| >12 | 1990-2005 | 0.72 (0.42 to 1.03) | 2.73 (0.57 to 4.94) | 0.25 (0.11 to 0.39) | 0.07 (-0.15 to 0.30) | -0.80 (-1.16 to -0.45) | 0.12 (-0.37 to 0.61) | 0.83 (0.25 to 1.41) | -0.08 (-0.68 to 0.52) |
|  | 2005-2018 | 0.45 (0.25 to 0.65) | -1.52 (-3.43 to 0.43) | -0.05 (-0.20 to 0.10) | 0.17 (0.05 to 0.30) | 0.56 (-0.68 to 1.82) | 0.97 (-0.51 to 2.47) | 0.83 (0.62 to 1.03) | 1.02 (0.12 to 1.92) |
|  | 1990-2018 | 0.61 (0.49 to 0.72) | 0.72 (-0.56 to 2.02) | 0.11 (0.04 to 0.18) | 0.07 (-0.01 to 0.14) | -0.04 (-0.43 to 0.35) | 0.50 (0.05 to 0.94) | 0.95 (0.77 to 1.14) | 0.44 (0.05 to 0.83) |
| **Area of residence** |  |  |  |  |  |  |  |  |  |
| Rural | 1990-2005 | 0.75 (0.38 to 1.13) | 2.41 (0.36 to 4.50) | 0.37 (0.20 to 0.54) | 0.21 (-0.01 to 0.42) | -1.30 (-1.83 to -0.76) | 0.59 (0.15 to 1.02) | 1.29 (0.66 to 1.93) | -0.29 (-0.66 to 0.07) |
|  | 2005-2018 | 1.56 (1.11 to 2.02) | -1.36 (-3.31 to 0.64) | -0.13 (-0.47 to 0.21) | 0.11 (0.06 to 0.16) | 0.97 (-0.89 to 2.86) | 1.15 (0.04 to 2.28) | 2.77 (2.09 to 3.46) | 2.26 (1.16 to 3.37) |
|  | 1990-2018 | 1.21 (0.96 to 1.45) | 0.62 (-0.56 to 1.82) | 0.11 (-0.02 to 0.25) | 0.12 (0.04 to 0.19) | -0.05 (-0.66 to 0.57) | 0.79 (0.45 to 1.12) | 2.20 (1.76 to 2.64) | 0.86 (0.21 to 1.52) |
| Urban | 1990-2005 | 0.61 (0.38 to 0.84) | 2.52 (0.38 to 4.71) | 0.24 (0.11 to 0.37) | 0.22 (-0.01 to 0.46) | -0.56 (-0.92 to -0.19) | 0.52 (0.08 to 0.96) | 0.43 (-0.11 to 0.98) | 0.01 (-0.42 to 0.44) |
|  | 2005-2018 | 0.56 (0.34 to 0.77) | -1.33 (-3.04 to 0.41) | -0.09 (-0.28 to 0.10) | 0.03 (-0.01 to 0.07) | 0.40 (-0.57 to 1.38) | 1.07 (-0.09 to 2.24) | 0.87 (0.74 to 1.01) | 1.28 (0.55 to 2.02) |
|  | 1990-2018 | 0.63 (0.54 to 0.72) | 0.68 (-0.51 to 1.88) | 0.08 (-0.00 to 0.16) | 0.09 (-0.00 to 0.17) | 0.06 (-0.27 to 0.39) | 0.69 (0.35 to 1.04) | 0.75 (0.54 to 0.96) | 0.61 (0.23 to 0.99) |

CI=confidence interval; EAPC=estimated annual percentage change.

The standardized serving size used for this analysis was a cup (8oz). Total green or black tea intake, including caffeinated, decaffeinated, sweetened or unsweetened tea. This definition excludes herbal tea.

*In previous Global Dietary Database reports, the region central or eastern Europe and central Asia was referred to as the former Soviet Union, and southeast and east Asia was referred to as Asia.

**Supplementary Table 14. Equally weighted regional tea intakes (cup (8 oz) per week) in 1990, 2005, and 2018 and EAPC from 1990-2005, 2005-2018, and 1990-2018 in adults aged ≥20 years.**

|  | **Mean intake (95% UI)** | | | **EAPC (95% CI)** | | |
| --- | --- | --- | --- | --- | --- | --- |
|  | **1990** | **2005** | **2018** | **1990-2005** | **2005-2018** | **1990-2018** |
| Central and eastern Europe and central Asia* | 4.83 (3.76 to 6.24) | 5.50 (4.30 to 7.14) | 5.09 (4.04 to 6.49) | 0.93 (0.26 to 1.59) | -0.83 (-1.66 to 0.02) | 0.04 (-0.47 to 0.55) |
| High income countries | 5.07 (4.01 to 6.55) | 5.10 (4.03 to 6.61) | 5.41 (4.29 to 6.98) | 0.01 (-0.62 to 0.63) | 0.37 (-0.19 to 0.94) | 0.24 (-0.01 to 0.48) |
| Latin America and the Caribbean | 3.77 (2.91 to 4.97) | 4.10 (3.15 to 5.43) | 4.36 (3.34 to 5.80) | 0.54 (0.12 to 0.96) | 0.54 (0.25 to 0.83) | 0.58 (0.45 to 0.71) |
| Middle East and north Africa | 8.28 (6.31 to 11.15) | 7.74 (5.87 to 10.47) | 7.51 (5.74 to 10.13) | -0.36 (-0.77 to 0.06) | -0.41 (-1.39 to 0.58) | -0.23 (-0.51 to 0.05) |
| South Asia* | 5.40 (4.29 to 6.97) | 5.08 (4.18 to 6.25) | 6.27 (4.96 to 8.11) | -0.39 (-0.82 to 0.05) | 1.98 (0.23 to 3.76) | 0.78 (0.15 to 1.42) |
| Southeast and east Asia | 4.54 (3.66 to 5.73) | 5.37 (4.33 to 6.76) | 5.91 (4.71 to 7.54) | 1.08 (0.64 to 1.52) | 0.93 (-0.45 to 2.32) | 0.88 (0.53 to 1.22) |
| Sub-Saharan Africa | 4.37 (3.67 to 5.30) | 4.07 (3.47 to 4.86) | 5.29 (4.44 to 6.43) | -0.32 (-0.95 to 0.31) | 2.02 (1.18 to 2.85) | 0.89 (0.34 to 1.45) |

CI=confidence interval; EAPC=estimated annual percentage change; UI=uncertainty interval.

The standardized serving size used for this analysis was a cup (8oz). Total green or black tea intake, including caffeinated, decaffeinated, sweetened or unsweetened tea. This definition excludes herbal tea.

*In previous Global Dietary Database reports, the region central or eastern Europe and central Asia was referred to as the former Soviet Union, and southeast and east Asia was referred to as Asia.


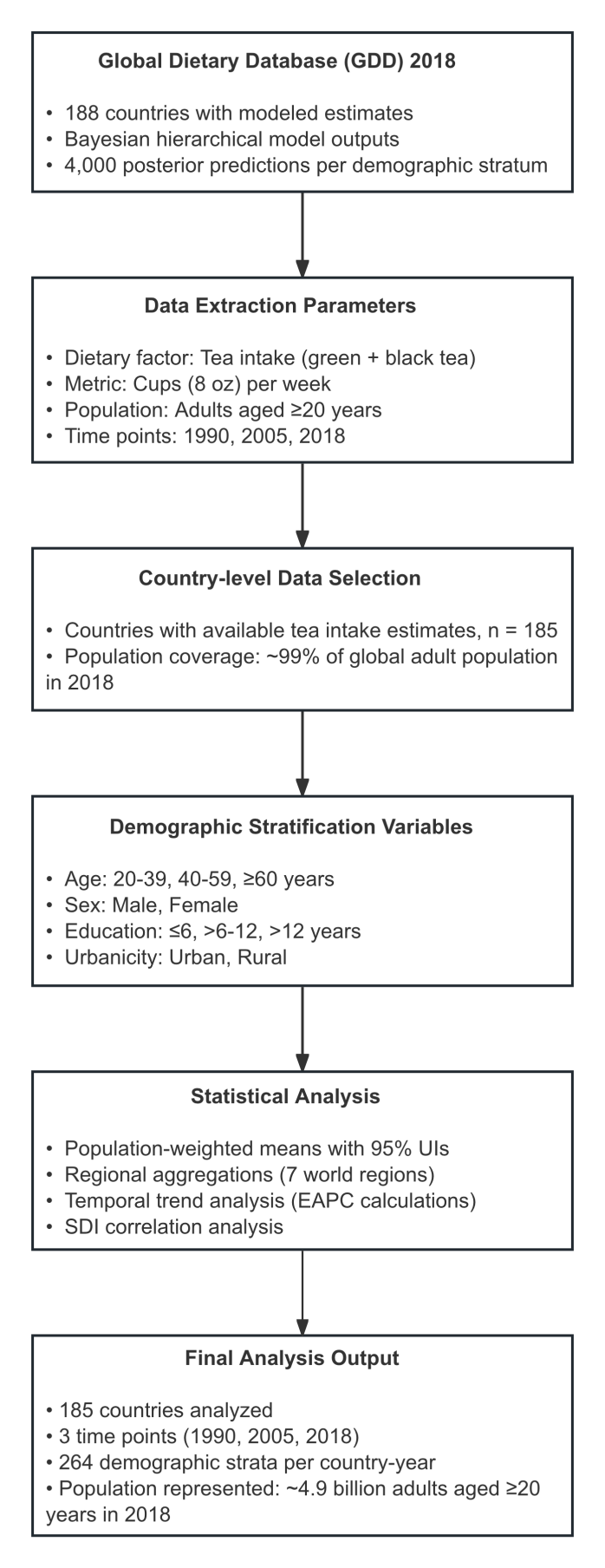


**Supplementary Figure 1. Flow chart of data extraction and analysis process from the Global Dietary Database 2018.** The diagram illustrates the systematic extraction of tea intake estimates and subsequent analytical approach. The GDD provided harmonized dietary intake estimates derived from 1,224 surveys using Bayesian hierarchical modeling. We extracted tea intake data for adults aged ≥20 years across 185 countries, stratified by demographic variables, for analysis of global patterns and temporal trends from 1990 to 2018. UI = uncertainty interval; EAPC = estimated annual percentage change; SDI = sociodemographic development index.


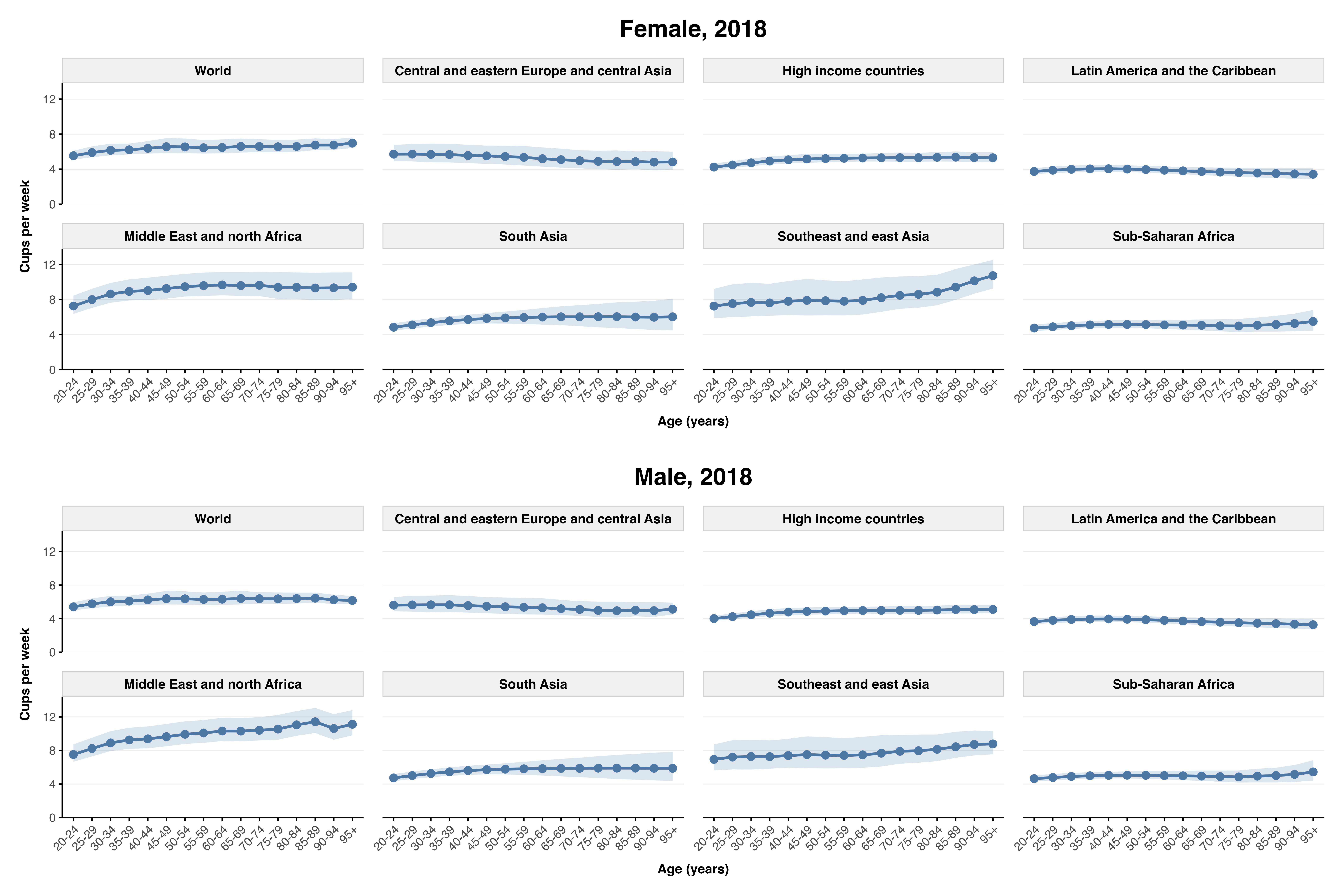


**Supplementary Figure 2. Global and regional intakes of tea (cup (8 oz) per week) by age among adults aged ≥20 years in females and males in 2018.** The standardized serving size used for this analysis was a cup (8oz). Total green or black tea intake, including caffeinated, decaffeinated, sweetened or unsweetened tea. This definition excludes herbal tea. The filled circles represent the mean tea intake (cup (8 oz) per week) and the shaded areas the 95% UIs. In previous Global Dietary Database reports, the region central and eastern Europe and central Asia was referred to as the former Soviet Union, and southeast and east Asia was referred to as Asia. UI=uncertainty interval.


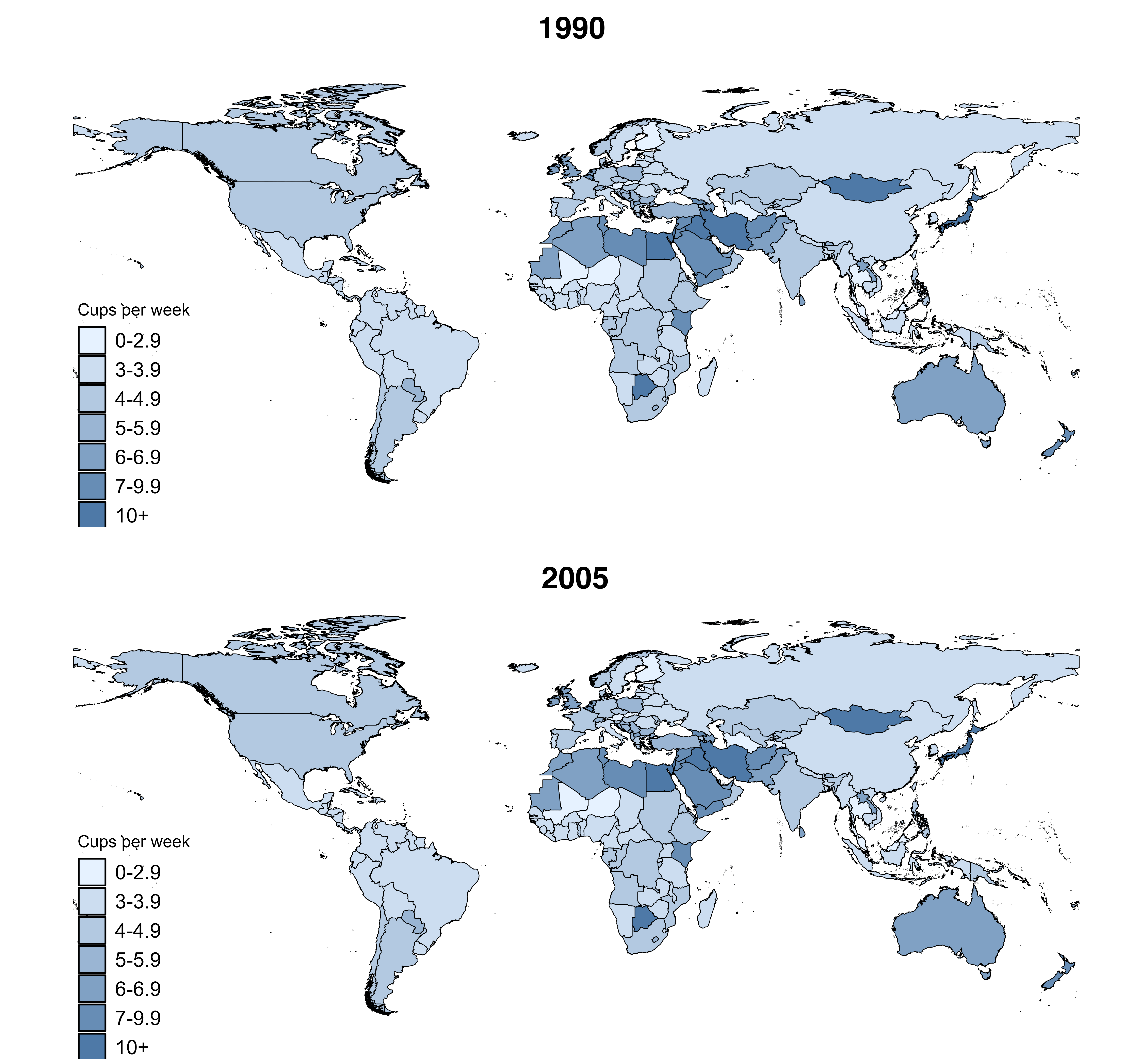


**Supplementary Figure 3. National mean intakes of tea (cup (8 oz) per week) in adults aged ≥20 years across 185 countries in 1990 and 2005.** The standardized serving size used for this analysis was a cup (8oz). Total green or black tea intake, including caffeinated, decaffeinated, sweetened or unsweetened tea. This definition excludes herbal tea.


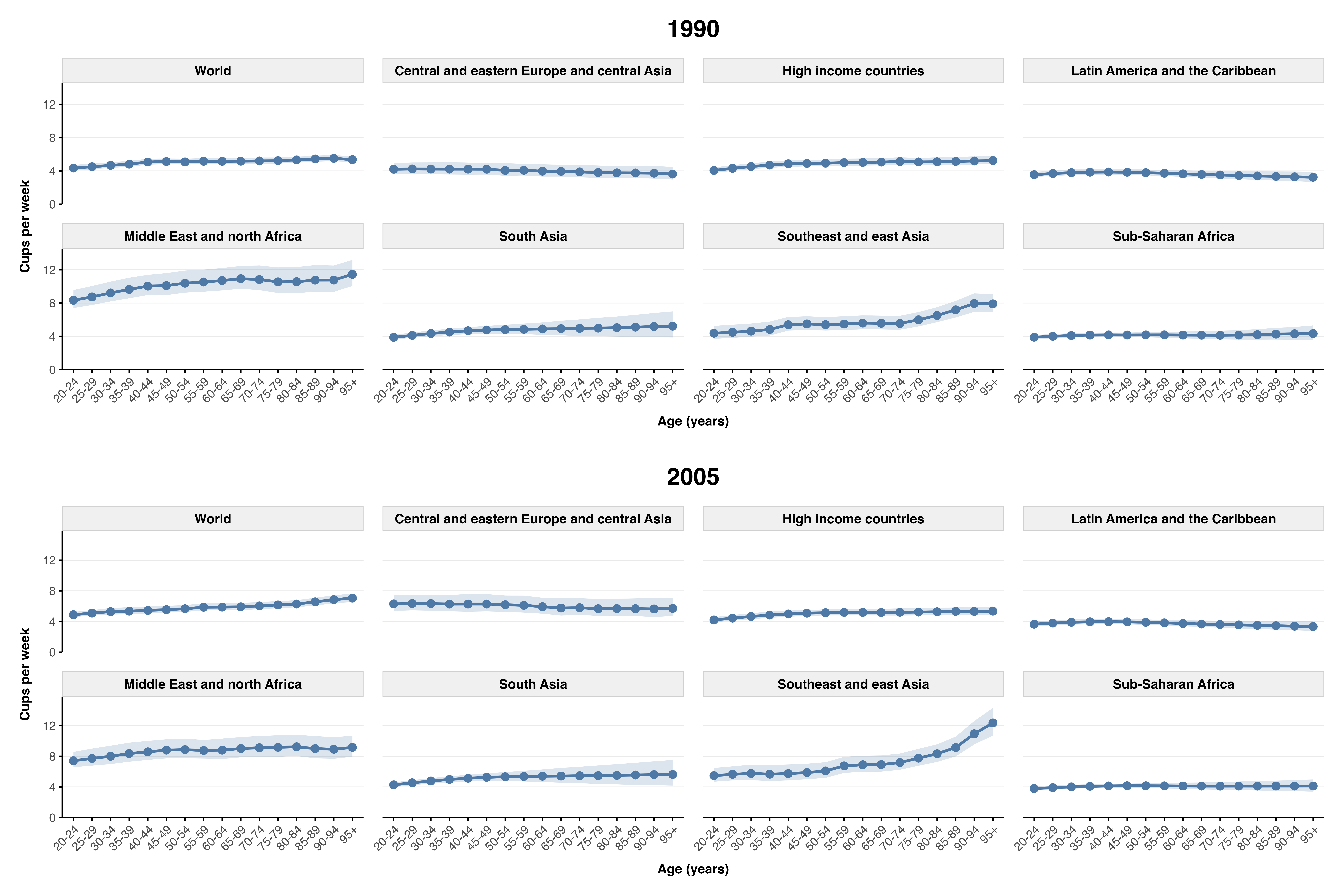


**Supplementary Figure 4. Global and regional intakes of tea (cup (8 oz) per week) by age in adults aged ≥20 years in 1990 and 2005.** The standardized serving size used for this analysis was a cup (8oz). Total green or black tea intake, including caffeinated, decaffeinated, sweetened or unsweetened tea. This definition excludes herbal tea. The filled circles represent the mean tea intake (cup (8 oz) per week) and the shaded areas the 95% UIs. In previous Global Dietary Database reports, the region central and eastern Europe and central Asia was referred to as the former Soviet Union, and southeast and east Asia was referred to as Asia. UI=uncertainty interval.


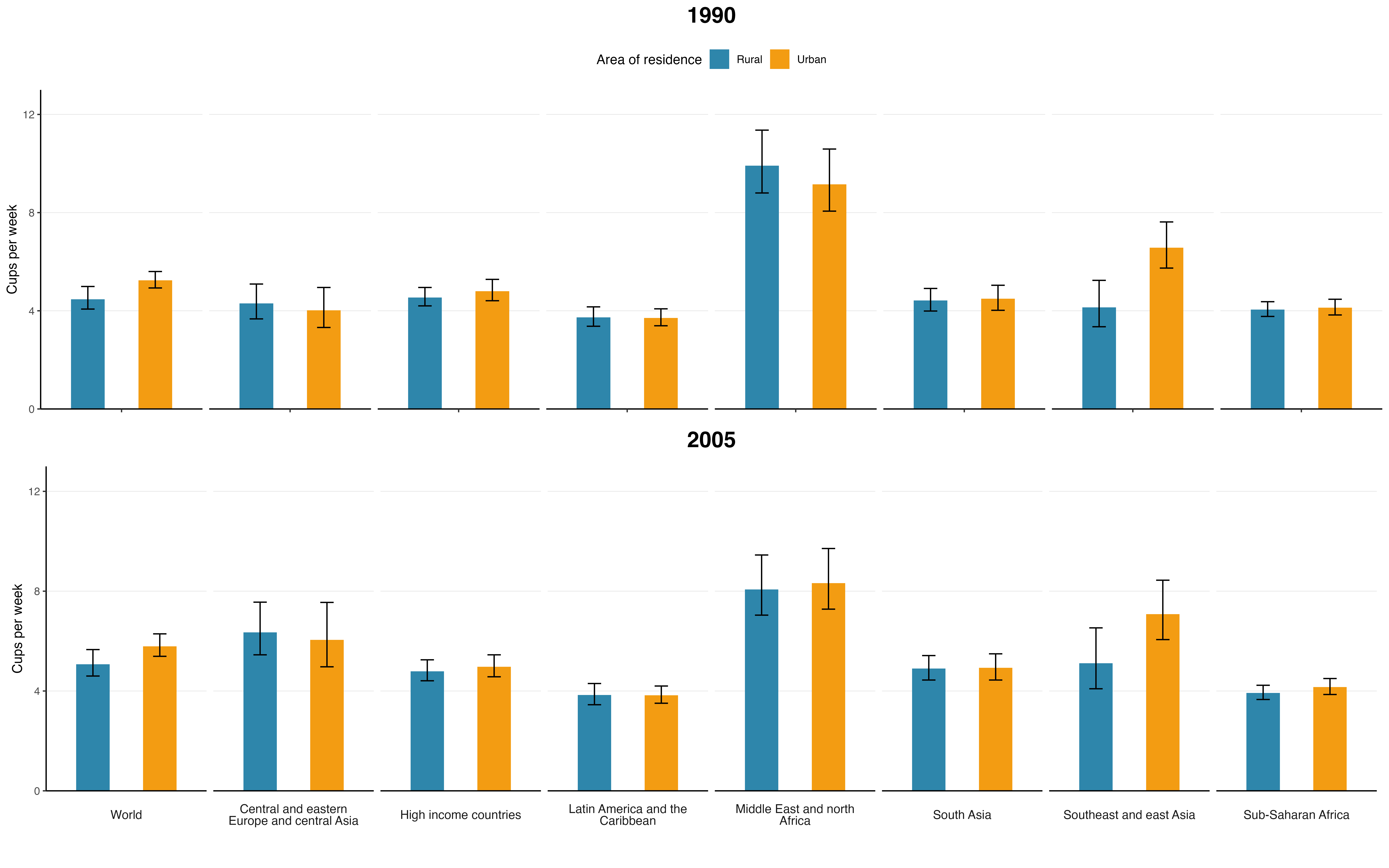


**Supplementary Figure 5. Global and regional mean tea intakes (cup (8 oz) per week) in adults aged ≥20 years by area of residence in 1990 and 2005.** Total green or black tea intake, including caffeinated, decaffeinated, sweetened or unsweetened tea. This definition excludes herbal tea. Error bars represent 95% UIs. In previous Global Dietary Database reports, the region central and eastern Europe and central Asia was referred to as the former Soviet Union, and southeast and east Asia was referred to as Asia. UI=uncertainty interval.


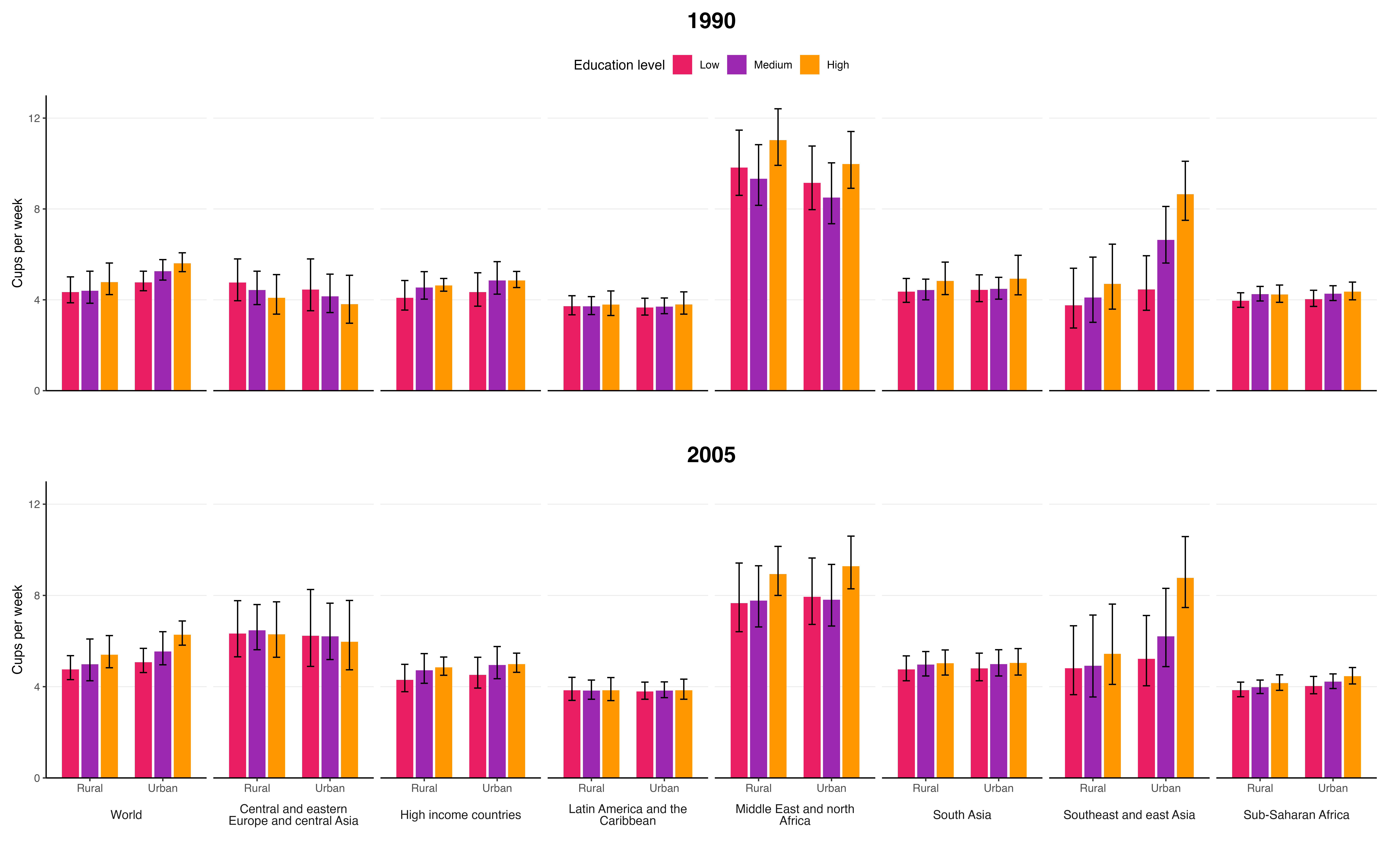


**Supplementary Figure 6. Global and regional mean tea intakes (cup (8 oz) per week) in adults aged ≥20 years by area of residence and education level in 1990 and 2005.** Total green or black tea intake, including caffeinated, decaffeinated, sweetened or unsweetened tea. This definition excludes herbal tea. Error bars represent 95% UIs. In previous Global Dietary Database reports, the region central and eastern Europe and central Asia was referred to as the former Soviet Union, and southeast and east Asia was referred to as Asia. UI=uncertainty interval.


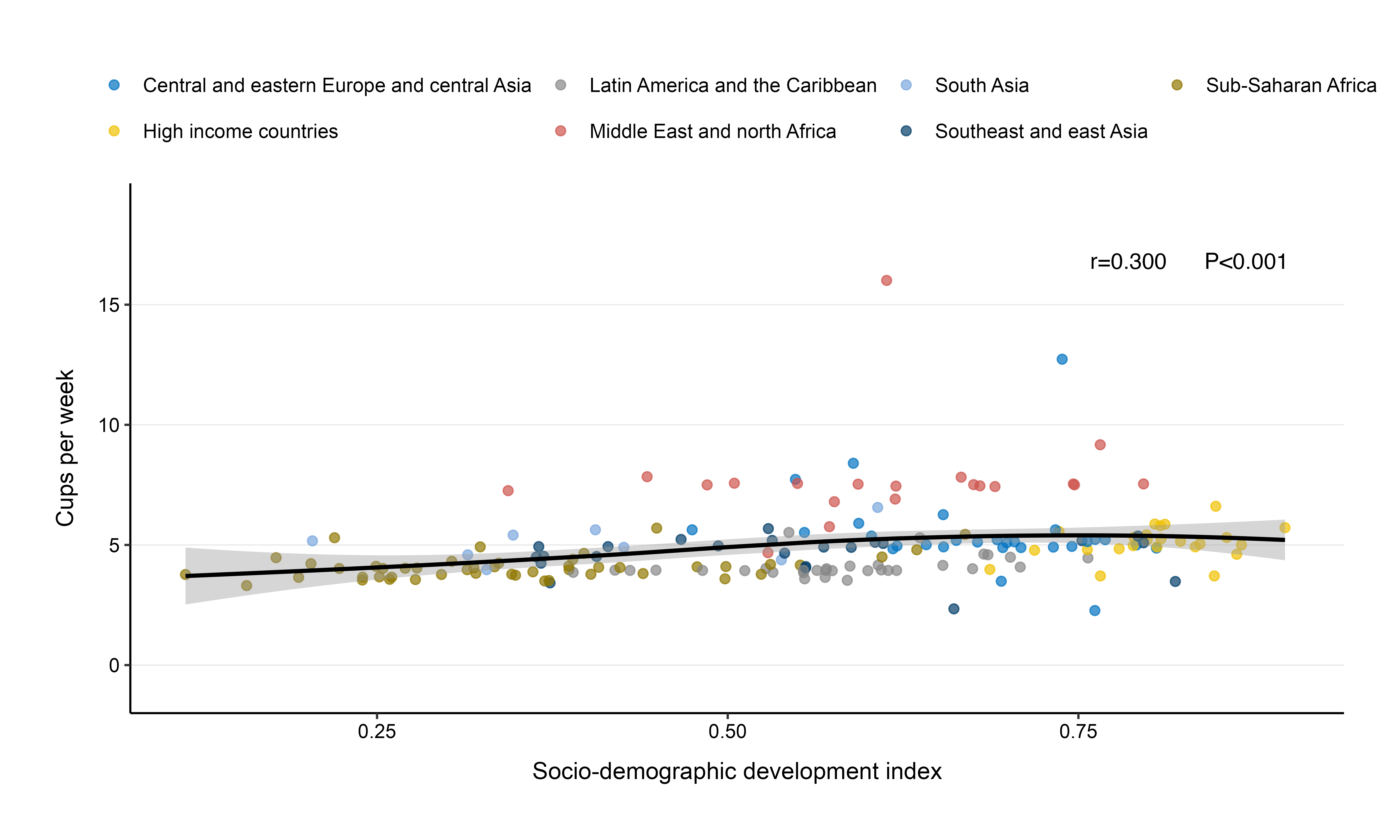


**Supplementary Figure 7. National correlation of tea intake (cup (8 oz) per week) in adults aged ≥20 years and socio-demographic development index by world region in 2005 for 185 countries.** Spearman correlation was assessed between SDI and tea intakes among a total of 185 countries were included in this analysis. The shaded areas the 95% CIs. Total green or black tea intake, including caffeinated, decaffeinated, sweetened or unsweetened tea. This definition excludes herbal tea. SDI was obtained from the Global Burden of Diseases study 2021. CI=confidence interval.
